# High-throughput toxicogenomic screening of chemicals in the environment using metabolically competent hepatic cell cultures

**DOI:** 10.1038/s41540-020-00166-2

**Published:** 2021-01-27

**Authors:** Jill A. Franzosa, Jessica A. Bonzo, John Jack, Nancy C. Baker, Parth Kothiya, Rafal P. Witek, Patrick Hurban, Stephen Siferd, Susan Hester, Imran Shah, Stephen S. Ferguson, Keith A. Houck, John F. Wambaugh

**Affiliations:** 1 Center for Computational Toxicology and Exposure, Office of Research and Development, U.S. EPA, Research Triangle Park, NC 27711 USA; 2 Cell Biology, Biosciences Division, Thermo Fisher Scientific, Frederick, MD 21703 USA; 3 Leidos, Research Triangle Park, NC 27711 USA; 4 Expression Analysis, Durham, NC 27713 USA; 5 Division of National Toxicology Program, National Institutes of Environmental Health Sciences of National Institutes of Health, Durham, NC 27709 USA

**Keywords:** Screening, Target identification, Genetic interaction

## Abstract

The ToxCast in vitro screening program has provided concentration-response bioactivity data across more than a thousand assay endpoints for thousands of chemicals found in our environment and commerce. However, most ToxCast screening assays have evaluated individual biological targets in cancer cell lines lacking integrated physiological functionality (such as receptor signaling, metabolism). We evaluated differentiated HepaRG^TM^ cells, a human liver-derived cell model understood to effectively model physiologically relevant hepatic signaling. Expression of 93 gene transcripts was measured by quantitative polymerase chain reaction using Fluidigm 96.96 dynamic arrays in response to 1060 chemicals tested in eight-point concentration-response. A Bayesian framework quantitatively modeled chemical-induced changes in gene expression via six transcription factors including: aryl hydrocarbon receptor, constitutive androstane receptor, pregnane X receptor, farnesoid X receptor, androgen receptor, and peroxisome proliferator-activated receptor alpha. For these chemicals the network model translates transcriptomic data into Bayesian inferences about molecular targets known to activate toxicological adverse outcome pathways. These data also provide new insights into the molecular signaling network of HepaRG^TM^ cell cultures.

## Introduction

Untested chemicals in commerce and the environment present a large and growing problem for public health risk assessment^1–4^: Which chemicals should be tested? Which should be of concern? Given this demand, numerous regulatory bodies worldwide have committed to evaluating and using new approach methodologies (NAMs) that are predictive of in vivo endpoints observed in traditional animal-based toxicity tests with the ultimate goals of increasing the efficiency of chemical hazard evaluation and reducing the reliance on animal testing^4^. Collins et al.^5^ write that the integration of in vitro high-throughput screening (HTS) into chemical decision-making may transform “toxicology from a predominantly observational science at the level of disease-specific models in vivo to a predominantly predictive science focused on broad inclusion of target-specific, mechanism-based, quantitatively relevant biological observations in vitro”.^5^ Over the last 10 years massive amounts of data have been generated using primarily in vitro-based HTS^5–8^. Early skeptics argued about the utility and relevancy of the data to traditional apical endpoints; however, subsequent data streams, tools, and methods which incorporated toxicokinetics and exposure science strengthened the case for the value of HTS data leading to the hallmark replacement of the in vivo rat uterotrophic assay by a combination of in vitro assays as part of the U.S. Endocrine Disruptor Screening Program^9^. However, endocrine signaling is relatively one of the better-covered biological pathways for existing HTS methods^10–12^. For HTS methods to succeed, they must cover a sufficient portion of biological space to allow the identification of all relevant in vivo effects (that is, toxicity pathways) in vitro^13^.

Two factors have been identified to increase the predictive capacity of the repertoire of existing HTS data produced by programs like U.S. EPA’s ToxCast (Toxicity Forecaster)^7^ and the U.S. Federal Tox21 consortium^14^: (1) using more physiologically relevant cell culture systems^15^ and (2) increasing coverage in biological response space. This study is an attempt to address both factors combining highly differentiated in vitro liver models with low density array transcriptomics.

The lack of physiologically relevant cell systems, in particular ones modeling liver effects on recognition and biotransformation of xenobiotic chemicals, has been criticized in many in vitro-based toxicity testing strategies^16–20^. The insufficiency or lack of metabolic competence in these in vitro systems can lead to potential mischaracterization of chemical hazard through both false positive (if the chemical is detoxified in vivo), as well as false negative (if the chemical is bioactivated in vivo) results. The widespread acknowledgment of this issue has prompted calls from the Organisation for Economic Co-operation and Development (OECD) and others for improved in vitro methods that provide liver functionality^21–24^.

Previous efforts within the ToxCast program used human primary hepatocytes which express the full suite of enzymes and transporters involved in hepatic metabolism^25^. The variability observed in the cells from different donors paired with the finite availability of human liver cells from an individual donor hampers the feasibility of using primary human hepatocytes to meet the functional and reproducibility needs of large in vitro screening efforts^25–27^ Although immortalized hepatoma-derived cell lines, such as HepG2, have met the needs of the ToxCast and Tox21 screening efforts by providing limitless supply and less phenotypic variability, these cells do not have a full repertoire of xenobiotic sensing receptors, metabolizing enzymes and transporters, deeming them potentially less effective for extrapolation to in vivo end-points, particularly for compounds known to be bioactivated or detoxified metabolically^26^. Here we used the HepaRG^TM^ cell culture model, derived from a human liver tumor^28^, which recapitulates numerous hallmarks of hepatocyte functionality in a model enabling year-over-year screening within a consistent genetic background^26,29–31^.

To expand the biological coverage, transcriptomics analysis was pursued^32^. Since toxicity is often accompanied by measurable changes at the transcriptional level, capturing the perturbations in gene expression elicited by chemical exposure at a cellular or tissue level provides a rich data source to interrogate pathway-based effects of adversity^33–35^. HTS was initially developed for drug discovery and typically used initial screening at single concentrations^5,36^. Adapting the technology to toxicology required concentration-response testing to minimize false negative results and therefore posed a bioinformatic challenge (many more data points per chemical) that has been largely overcome^34,37–40^. Transcriptomics is very attractive to increase coverage of biological response space, for any one concentration-response experiment, numerous transcription outputs may be examined to both screen more widely and deduce interactions caused by chemical perturbations^19,32,37,41–44^. Managing the expansion of data posed by concentration-response transcriptomics on thousands of chemicals is a new bioinformatics challenge^32^.

Here we have used the principles of the adverse outcome pathway (AOP) framework^45^ in an integrated approach to testing and assessment (IATA) to evaluate existing data (for example, nuclear receptor activation) to selectively gain valuable information on downstream key events to strengthen our predictions of pathway-level perturbations that may result in adverse phenotypes in whole tissues^35^. The gene transcription responses examined here do not directly demonstrate receptor activation, but do allow for inference of activation, potentially informing AOPs. Each AOP begins with a “molecular initiating event”, in this case receptor activation. We assume that each receptor is “switch-like”; activating a receptor sets the same transcriptional machinery in motion regardless of the chemical that causes the activation. Thus, here the receptor-mediated pattern of transcriptional activation/deactivation is assumed to be consistent across all chemical activators of a receptor. The patterns from multiple, simultaneously activated receptors are assumed to be additive or subtractive—we have neglected higher order interactions. Based on these assumptions we can characterize receptor-mediated, chemical-induced perturbations by examining 93 transcripts for 10 reference chemicals. We used a Bayesian framework first to integrate peer-reviewed literature data and new transcriptomic data collected for reference chemicals.

By determining the transcriptional patterns for reference receptor activators, we can then screen new chemicals by comparing their concentration response mRNA expression data to the reference patterns to estimate both potency and likely target receptor(s). Here we tested the 1060 chemicals from the ToxCast library^46^ in eight-point concentration response. We used the reference patterns of upregulation and downregulation across the assayed transcripts as transcriptional signatures. We searched the screening library transcriptional concentration-response data for these signatures using Bayesian analysis. We assume that chemicals vary with respect to potency for each receptor; that is, a given chemical will activate different receptors (if at all) in various patterns as concentration increases. We draw inferences about possible molecular initiating events (that is, upstream receptor activity) and potency across the chemical screening library. Many of the chemicals screened here lack in vivo testing data; this new assay provides assessment of how likely these chemicals and/or their metabolites are to perturb a variety of toxicity pathways and hepatic functions.

## Results

We collected transcriptomic data for a library of chemicals from commerce and the environment in order to screen for potential biological interactions. The 93 transcripts assayed by these experiments were selected with specific emphasis on Phase I and Phase II metabolizing enzymes, transporters, and receptor signals known to be modulated by environmental chemicals. In addition, cell-cycle progression and morphogenesis genes potentially related to mechanisms of cancer were selected. All tested transcripts and rationale for inclusion are listed in Supplementary Table 1. The concentration-response data for each transcript provides two ToxCast assay ‘endpoints’ as each curve was fit separately in up and downregulation mode. The concentration-response relationships considered included both a monotonic Hill function and a gain-loss model, but any loss of signal at high concentration was interpreted as being due to cytoxicity^38^. The lactate dehydrogenase (LDH) assay provides one additional assay endpoint. To distinguish from other ToxCast assays, we denote this set of assay endpoints as “LTEA” for the two contractors Life Technologies and Expression Analysis who performed the assays. After the first reference plate of chemicals was tested, the results for the 93 transcripts were manually investigated for performance and five probes were identified as having too many non-detects. Four of the five (for transcripts KLK3, MMP1, SLC10A1, and SLC22A6) were replaced with probes for different transcripts (MIR122, NFE2L2, PDK4, and XBP1). The original probe for CYP24A1 (TaqMan assay Hs00167999_m1) was replaced by probe Hs00989017_m1, also for CYP24A1, since previous research has demonstrated the presence of that transcript in HepaRG cultures^29^.

Across the 1060 chemicals, 1037 chemicals (98%) had at least one systematic relationship between concentration and transcriptional response. However, if all relationships with curve-fit warning flags^11^ are omitted (a very conservative assumption with respect to true activity), only 718 (68%) of chemicals had a clear systematic response. Among the 718 chemicals, the median number of responding transcripts was 6, with a maximum of 90 (for the chemical mancozeb). The most commonly occurring responses were upregulation of CYP1A1 (360 chemicals), upregulation of CYP2B6 (352), and downregulation of CYP2E1 (323). Sixty-five percent of the chemical-transcript pairs demonstrated an upregulation relationship while 35% demonstrated a downward relationship.

**Fig. 1:**
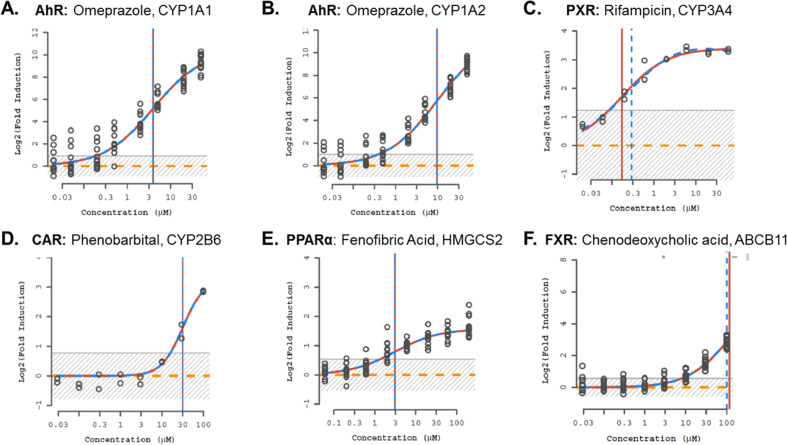
Dose-response curves for reference chemicals and transcriptionally regulated genes. Log2 (Fold Induction) response profiles of (**A**) CYP1A1, (**B**) CYP1A2 upon exposure to AhR positive control, omeprazole; (**C**) CYP3A4 with rifampicin treatment; (**D**) CYP2B6 in response to CAR-inducing, phenobarbital exposure; (**E**) HMGCS2 after treatment PPARα positive control, fenofibric acid and (**F**) ABCB11 expression with chenodeoxycholic acid treatment. Three dose-response relationships are indicated in each plot, the first (no response) is a horizontal long-dashed line, while the Hill function (short-dashed line) and gain-loss (solid line) response models change with the points. The vertical lines indicate the estimate 50% activation concentration (AC50) for the two response models. The gray shaded region indicates estimated background.

We used reference chemicals and transcriptomic data to elucidate the gene regulation network. Figure 1 demonstrates that canonical response genes downstream of receptor targets were identified as active when probed with reference compounds. Omeprazole was active against CYP1A2 and CYP1A1 (corresponding to AhR^25,47^); fenofibric acid was active against HMGCS2 (PPARα^25^); rifampicin against CYP3A4 (PXR^25,48^); phenobarbital against CYP2B6 (CAR^25^); and chenodeoxycholic acid was active against ABCB11 (FXR^25,49^). All data are available at ftp://newftp.epa.gov/COMPTOX/CCTE_Publication_Data/CCED_Publication_Data/Wambaugh/ToxCast_LTEA, in files LTEA_Level2_20191119.zip (raw, unnormalized data) and LTEA_Level5_20191119.zip (results of concentration-response curve-fitting).

### A metabolically competent ToxCast assay for transcription response

Since the goal of this approach was to study transcriptional activity, an “induction medium” (0.5% dimethyl sulfoxide or DMSO) was used to intentionally lower the basal gene expression of DMSO-induced drug metabolizing enzymes to better reflect a Zone-2 like state of hepatocyte differentiation, which retains both baseline and inducible drug metabolizing activities in response to chemical exposures. This may reduce the rate of xenobiotic metabolism observed with high (for example, 2%) DMSO concentrations that have been used elsewhere with HepaRG^TM^ cultures^50,51^.

**Fig. 2:**
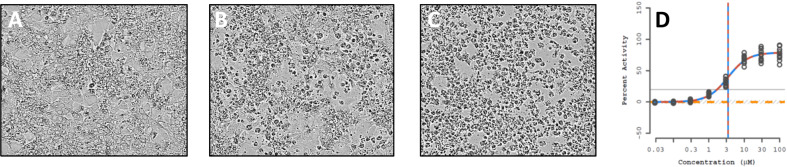
Visual assessment of aflatoxin B1 (AFL) cytotoxicity in cells. Images of cells treated for 48 h with (**A**) 0.5% DMSO vehicle control, (**B**) 3.16 μM AFL (~EC10) or (**C**) 100 μM AFL. (**D**) LDH assay dose-response curve for AFL treated cells.

The cell morphology of HepaRG^TM^ cultures treated with vehicle (0.5% DMSO) confirmed healthy, well-differentiated cell cultures composed of the two distinct cell morphologies typical of HepaRG^TM^ cell cultures. Treatment with the metabolic positive control aflatoxin B1, a mycotoxicant, resulted in visually apparent cell death (Fig. 2). Since the cytotoxicity of aflatoxin B1 is known to be metabolically-dependent^52^, the observed cytotoxicity demonstrated HepaRG^TM^ metabolic activity was consistent throughout the in vitro screening. The concentration-dependency of toxicity was evident with fewer dead cells present with a lower (3.16 μM) concentration of aflatoxin B1 (Fig. 2B) compared to the highest concentration tested (100 μM, Fig. 2C). Thus, cell morphology images support the LDH assay data suggesting sufficient metabolic competence of the culture model consistent with what has been observed elsewhere^50^. As shown in Fig. 2D, aflatoxin B1 treatment resulted in a concentration-related increase in cytotoxicity as measured by LDH release consistent with a metabolically competent in vitro liver model. Aflatoxin B1 was included on the six reference plates tested throughout the screening, and in all these reference plates the HepaRG^TM^ cultures showed aflatoxin B1-induced cytotoxicity in a concentration-related manner. All image data are also available at ftp://newftp.epa.gov/COMPTOX/CCTE_Publication_Data/CCED_Publication_Data/Wambaugh/ToxCast_LTEA.

A second line of evidence for metabolic competence is the induction of CYP3A4 mRNA expression by omeprazole. Omeprazole is a reference AhR agonist which typically does not induce genes such as CYP3A4^48^. However, omeprazole is also known to be extensively metabolized in humans to multiple metabolites, including sulfone metabolites, that have been shown to induce CYP3A4 with cultures of primary human hepatocytes^53^. In this study, omeprazole produced a robust (4.2-fold) induction of CYP3A4 after 48 h in response to omeprazole exposures, which is consistent with observations in other metabolically-competent systems^54^.

**Fig. 3:**
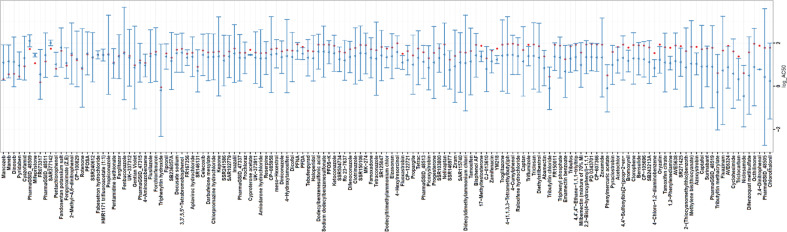
Comparison of cytotoxic activity. Using cells with xenobiotic metabolism capacity may permit identification of compound toxicity resulting from a chemical metabolite. **A** The log AC50 values (dot) for the compounds positive in the HepaRG^TM^ cytotoxicity assay, as determined by measure of lactate dehydrogenase (LDH) release, were compared with a range of cytotoxicity measurements made in other cell lines and primary cells (whiskers). Cytotoxicity measurements for the other cell types used a range of techniques, primarily ATP level determinations, but did not include LDH release While assay-specific sensitivities may influence results, overall, few compounds showed enhanced cytotoxicity in the HepaRG^TM^ cells.

Analysis of the LDH assay concentration response data provided an additional ToxCast assay endpoint characterizing cytotoxicity with increasing test chemical concentration. In Fig. 3, the log_10_ AC_50_ values for the compounds positive in the HepaRG^TM^ cytotoxicity assay were compared with a range of ToxCast cytotoxicity measurements made in other cell lines and primary cells. Cytotoxicity measurements for the other cell types used a range of techniques, primarily ATP level determinations, but did not include LDH release^55^. Overall, few compounds showed enhanced cytotoxicity in the HepaRG^TM^ culture for the concentrations and duration of exposure examined relative to the ranges of ToxCast cytotoxicity observed to date. Further evaluation of these findings in context with information related to chemical specific bioactivation or detoxification and varied culture media composition is warranted.

### Inferring molecular initiating events

**Fig. 4:**
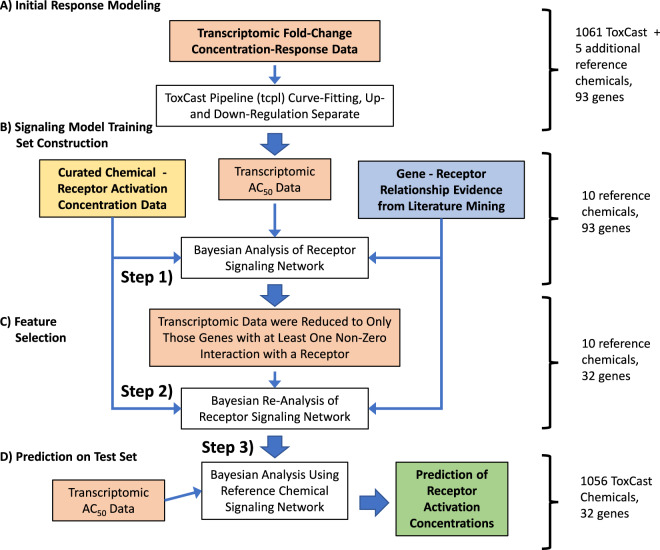
Overview of analysis. Initial curve fitting was performed to identify concentrations causing fifty percent activity (AC50), if any. The signaling network model was then trained in three steps (see Table 1). Transcriptomic, gene-receptor literature data, and chemical-receptor potency data are shaded differently.

By identifying patterns of upregulation and downregulation that occur when a receptor is activated, our probabilistic model for gene regulation detangled multiple receptor activations from any noise, and identified potencies (chemical concentrations) associated with those activations. Molecular initiating events (that is, receptor activation) were inferred in a three step Bayesian analysis described in detail in the “Methods” section and summarized in Fig. 4 and Table 1.

**Table 1:** A Bayesian analysis combines prior information with new data to generate a posterior distribution reflecting the likely outcomes based on both the prior and the new data.

Bayesian Analysis	Description	Data	Prior	Posterior
Step One	Univariate (one receptor at a time) analysis of reference chemicals	Full reference chemical concentration-response data for all reference chemicals and only those transcripts where a change was observed.	Literature associations between reference chemicals and transcripts under investigation. Texting mining of MeSH term co-occurrence for receptors and transcripts.	Estimates of strength of interaction for every receptor and all transcripts where the reference chemicals displayed activity.
Step Two	Multivariate analysis of reference chemicals	Full reference chemical concentration-response data for all reference chemicals and only those transcripts where there was a 50% or greater chance of interaction in Step One.	Literature associations between reference chemicals and transcripts under investigation. Texting mining of MeSH term co-occurrence for receptors and transcripts.	Estimates of strength of interaction for every receptor and every transcript identified as likely to be associated with a receptor in Step One.
Step Three	Multivariate analysis of test chemicals	Full concentration response data for all test chemicals for the same transcripts as Step Two.	The posterior from Step Two: a correlated, multivariate normal distribution of receptor-transcript interactions.	Estimates of the probability and potency of receptor activation for all test chemicals.

### Prior information on reference chemical potencies

**Fig. 5:**
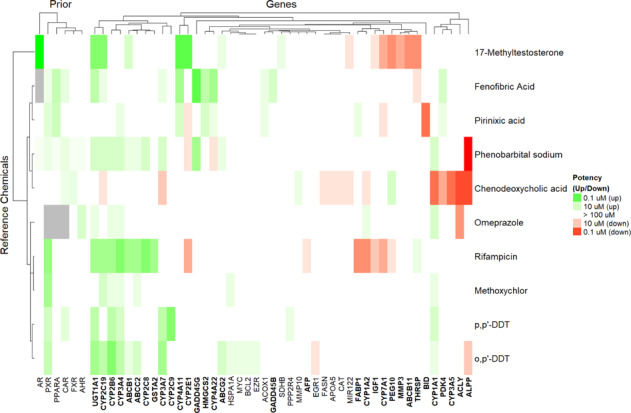
Transcriptional behavior of reference chemicals. At the left-hand side the literature-curated receptor potencies are indicated, while at the right-hand side the heatmap shows the potency (darker indicates more potent) and direction (color) of the 45 transcripts found to be systematically altered by increasing concentration of the reference chemicals. The 32 transcripts found to have at least a 50% chance of non-zero interactions in the multivariate analysis are indicated in bold. Chemicals and transcripts are clustered hierarchically such that those showing more similar behavior are closer together. The heatmap was created with R package ComplexHeatmap^103^ using hierarchical clustering with complete linkage and Euclidean distance.

Four receptor-activating reference chemicals were included on the reference plates that were repeatedly tested throughout the screening process. In vitro screening literature were curated to determine known potencies for the various reference chemicals across six receptors (Supplementary Table 2). Information was not available for all chemical-receptor combinations, and many chemicals showed some potency for multiple receptors (left-hand side of Fig. 5). Phenobarbital was run at a high concentration (500 µM max) on the reference plates, as well as part of the ToxCast screening library (100 µM max). To clarify receptor activation profiles, six additional reference chemicals with known agonism were also selected from among the screening library. As is shown in Fig. 5, literature data indicate that our screening library contained only one reference compound for three of the receptors (FXR, AhR, and AR are, respectively, activated by chenodeoxycholic acid, omeprazole, and methyl testosterone). Both chemicals that activate PPARα (fenofibric acid and pirinixic acid) also activate additional receptors. Four chemicals activate CAR (phenobarbital sodium, chenodeoxycholic acid, fenofibric acid, and p,p’-DDT), while seven chemicals activate PXR (phenobarbital sodium, fenofibric acid, pirinixic acid, p,p’-DDT, o,p’-DDT, rifampicin, and methoxychlor). Data for omeprazole are the most incomplete, while phenobarbital sodium is the least potent of any of the reference chemicals considered.

### Receptor-gene literature

Separately from the reference chemicals, automated literature searches provided independent evidence of interactions between a given receptor and gene expression. A histogram of the number of literature occurrences associating one of the six receptors and one of the 93 transcripts is shown on the right hand-side of Supplementary Fig. 1. Most genes and receptors that co-occur do so in the MeSH terms for fewer than ten peer-reviewed journal articles, while a handful co-occur hundreds of times. The occurrence of up and downregulation relationships are roughly evenly split, while more enzyme induction relationships are observed than repression (right-hand side of Supplementary Fig. 1). All the literature data were used as prior evidence for a regulatory interaction (up or down) between a receptor and a transcript.

### Reference chemical data

One chemical (aflatoxin B1) was used to test for consistent metabolic competency throughout the testing. In addition to inclusion at a single concentration (*n* = 3 wells) on each test plate, aflatoxin was one of five chemicals included in concentration-response on each of the reference plates (Supplementary Fig. 2) interspersed throughout the testing process. The reference plates also included four of the reference receptor activator chemicals. Three of the reference receptor activator chemicals (omeprazole, fenofibric acid, chenodeoxycholic acid) only appeared on the reference plates, producing 12 replicates of the data for analysis. Neglecting outliers (a few extreme cycle threshold (Ct) values less than −10) for these three reference chemicals the median standard deviation of Ct across all endpoints was 0.62. The three least varying endpoints were TFGA, TIMP1, and SLC10A1, while the three most variable endpoints were cytotoxicity, MIR122 and MMP10. A fifth chemical (phenobarbital) was included on the reference plates (max concentration 500 µM), tested as part of the ToxCast library (max concentration 100 µM) and included at a single concentration (1 mM, *n* = 3 wells) on every test plate. This resulted in a total of 678 replicates of data. For phenobarbital the median standard deviation was 0.65, the three most reproducible endpoints were FASN, TGFB1, and SLC10A1, while the most variable were cytotoxicity, MMP10, and MMP1. Six additional reference activator chemicals were selected from among the test library and only tested in duplicate.

Collectively, the reference chemicals helped to identify the transcriptional responses most likely representative of a receptor’s activation. The transcriptomic data for the ten reference activator chemicals were analyzed twice. The 93 transcripts were manually selected based upon expert opinion of the authors. Therefore, as a feature selection step, all 93 transcripts were first analyzed to detect any switch-like behavior (upregulation or downregulation) at the inferred activation concentration for each reference chemical and receptor. Though the model assumes that different reference chemicals will activate receptors at different concentrations, the subsequent receptor-mediated pattern of transcriptional activation/deactivation was assumed to be consistent between all chemical activators of a receptor (that is, the activity results from the operation of the same biochemical machinery independent of the presence of the activating chemical). Across the ten reference chemicals, only 45 transcripts responded to increasing chemical concentration in a systematic manner (Hill function or gain-loss, that is, a “hit”) for any receptor. While the other transcripts are also likely markers of important cellular perturbations, they were set aside for the initial analysis of the activation of the six receptors. The model used for analyzing the expression data are not deterministic, rather there is always a transcript-specific probability (${\mathrm{thresh}}_i$) that a transcript remains at a basal expression level even if upregulatory or downregulatory signals are received. Analysis of the heavily duplicated reference chemicals allowed estimation of the responsiveness of each transcript to signaling.

The right-hand side of Fig. 5 displays the potencies and upregulatory or downregulatory behavior for the 45 differentially expressed transcripts. Both chemicals and genes are clustered according to similar behavior. We observe that 17-methyltestosterone is the most distinct chemical, downregulating a series of transcripts that are largely unaffected by the other chemicals, except perhaps for rifampicin. A group of genes at the far left are all downregulated by chenodeoxycholic acid, indicating a potential signature for FXR agonists. Although the two PPARα agonists are clustered together, a distinct pattern for PPARα activation is less obvious, perhaps reflecting a lack of coverage of PPARα-regulated genes by the 93 transcripts. The PXR activators are all clustered together, but the much less potent and more CAR-selective phenobarbital exposure is somewhat separated from the PXR cluster.

**Fig. 6:**
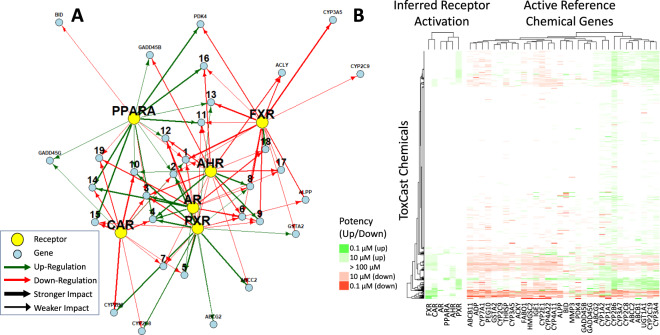
Inferred signaling network and receptor activity. The network at the left (panel **A**) represents the direction, magnitude of interaction, and cross-talk for the six receptors in HepaRG^TM^ cultures as determined using the entire chemical library as a set of test perturbations. Genes regulated by three or more receptors are labeled numerically, as described in Table 2. The left-hand side of the heatmap (panel **B**) indicates the relative potency inferred for the six receptors, while the right-hand side of Panel **B** presents the observed transcriptional response transcripts identified as part of the reference chemical signatures. The heatmap was created (with a function provided in the section “R Code” of the Supplementary Materials) using hierarchical clustering with complete linkage and Euclidean distance.

For computational efficiency the first step was conducted for each receptor individually. Despite showing systematic response to increasing concentration of at least one reference activator chemicals, regulation of 13 of the 45 transcripts was found to be unlikely to be associated with receptor activation (ACOX1, APOA5, BCL2, CAT, EGR1, EZR, FASN, HSPA1A, MIR122, MMP10, MYC, PPP2R4, and SDHB). A second analysis refined the signaling network by jointly estimating interactions between the receptors and the 32 remaining transcripts. This analysis provides a receptor activity inference model that was used to analyze the non-reference chemicals in the ToxCast chemical library. This analysis of the reference chemicals and all receptors simultaneously was conducted including only those 32 transcripts that were significant in the first analysis. This analysis identified patterns of transcriptional activity corresponding to each of the six different molecular initiating events. The inferred signaling network for HepaRG^TM^ cells is depicted in Fig. 6A. Substantial cross-talk was observed, so individual network diagrams for each receptor are provided in Supplementary Fig. 3.

Crosstalk between receptors is a key observation of our study. Only three of the 32 transcripts (BID, CYP2C9, and CYP3A5) appeared to be regulated by a single receptor. Those transcripts that were found to be regulated by more than two receptors are numbered in Fig. 6A, and their identities are given in Table 2. PPARα and FXR appear to be more distinct from the other four receptors. AR and AhR are distinctly closer to PXR than CAR. Proximity between receptors may be related to the unequal numbers of transcripts corresponding to each receptor. The largest observed effects are upregulation of CYP1A1 by AhR, upregulation of CYP3A4 by PXR, and more surprisingly, downregulation of IFG1 by AR. Interestingly, CAR and PXR are considered to be closely related xenobiotic sensing receptors^56,57^, and CYP2B6 is considered to be upregulated by both^58^. We found that while PXR strongly upregulated CYP2B6, CAR downregulated it. This could be a result of the selected reference chemicals—of three reference CAR activators two of the three (rifampicin and phenobarbital) activated both CAR and PXR (see Fig. 5 and Supplementary Fig. 3). Both of those chemicals were observed to induce CYP2B6. As shown in Fig. 5, only one reference chemical, chenodeoxycholic acid, is believed to act on CAR but not also activate PXR. In the absence of PXR activity, chenodeoxycholic acid downregulated CYP2B6. Chenodeoxycholic acid was tested in duplicate on our six reference plates. So, as shown in Fig. 6a and Table 2, our analysis inferred that CAR downregulates CYP2B6. However, chenodeoxycholic acid concurrently activates FXR, which might interfere with CYP2B6 activation by CAR. Other possibilities include that HepaRG^TM^ CAR-CYP2B6 signaling deviates from other cells or that other literature evidence of upregulation of CYP2B6 by CAR may have been confounded by PXR activation.

**Table 2:** The list of genes used to identify receptor activity based on analysis of reference chemicals.

Gene	CYP7A1	CYP2C19	CYP2E1	UGT1A1	ABCB1	ABCB11	AFP	CYP1A1	CYP1A2	CYP3A4	FABP1	IGF1	CYP3A7	CYP4A11	CYP4A22	HMGCS2	MMP3	PEG10	THRSP	ABCC2	ABCG2	ACLY	ALPP	CYP2B6	CYP2C8	GADD45B	GADD45G	GSTA2	PDK4	BID	CYP2C9	CYP3A5
Fig. 6 Label	1	2	3	4	5	6	7	8	9	10	11	12	13	14	15	16	17	18	19													
AHR	−		−−	++	−	−−	−	++	++	+	−	−-				−−	−−	−		−		−−				−−						
AR	−	++	++	++	+	−−	−	−	−			−−		++			−	−−	−−		+											
CAR	+	−	−−	−−	−		−			−−				−−	−−				−−					−−	−		+					
FXR	−−	−				−		−−	−−		−		−−			−	−	++				−−	−−					−	−−		−	−−
PPARA	−	+	−	++						+	++	+	+	++	++	++			−							+	+		++	−		
PXR	−	++	−−	++	++	−	−	+	−	++	−	−	+		−					++	+		−	++	++			+				

“+” indicates upregulation, “−” indicates downregulation. “++” and “−−” indicate above median receptor-gene strength of interaction, while “+” and “−” indicate below median interaction strength. Genes with more than two receptor interactions are labeled numerically in Fig. 6.

The transcripts in Table 2 are ordered from highest “degree” (number of regulating receptors) to lowest. CYP7A1 was found to be regulated by all six receptors; this gene is associated with regulation of cholesterol and bile acid homeostasis^59,60^ and its promiscuity among the receptors might therefore be related to biliary clearance of compounds. Strength and direction (up/downregulation are also indicated in Table 2. “++” and “−−” indicate regulation with a strength estimated to be greater than the median interaction, while “+” and “−” indicate below median interaction strength. UGT1A1 is strongly regulated by five of the six receptors (FXR excepted). UGT1A1 is involved in synthesis of bilirubin^61^, again pointing to receptor activation being directly linked to biliary clearance.

The amount of crosstalk that was observed strongly supports the need to use a pattern of regulation^58^ to identify receptor activation (that is, all transcripts indicated by a row in Table 2) rather than individual transcripts.

### ToxCast chemical data

We used the patterns of transcriptional activity resulting from receptor activation, as identified from the reference chemicals, to assess the probability of those events occurring as a function of concentrations for the 1053 test chemicals from the ToxCast screening library^46^. Figure 6B shows the transcriptional response observed for the 32 transcripts that comprise the receptor activity patterns. Most chemicals do not show notable activity. Correspondingly, when the receptor activity inference model is used, as on the left-hand side of the heatmap in Fig. 6B, no receptor activity was inferred for 43% of the chemicals and only 37% have any activity inferred below 100 µM. However, receptor activation was inferred for some chemicals, with many having multiple activities inferred: below 100 µM (the highest test concentration) PXR is the most common (18%), followed by CAR (17%), FXR (12%), AhR (11%), PPARα (3.8%), and AR (0.98%). All data and results are available in Supplementary Table 3, Supplementary Table 4, Supplementary Table 5, and Supplementary Table 6.

## Discussion

HTS is a new approach methodology that may be used to protect public health from unintended chemical effects^4^. This study attempts to improve the predictive capacity of HTS by both increasing coverage in biological response space and using more biologically relevant cell culture systems. Because of the difficulties in obtaining pure, testable samples of most metabolites^46^, to date HTS has focused on parent chemicals, regardless of whether those chemicals are metabolically transformed, activated, and/or deactivated in vivo. The initial phase of the ToxCast project incorporated metabolically competent primary human hepatocytes to profile the impact on 14 transcripts for 309 chemicals^25^. These Rotroff et al. data have proved useful as a component of predictive models for in vivo effects^62–66^. However, the limited supply and pronounced variability of human primary hepatocytes pose serious statistical issues as part of a screening panel for larger libraries of chemicals. Here, we have examined the human liver derived HepaRG^TM^ cell culture as a more stable, year-over-year available, and reproducible cell model conferring xenobiotic recognition and liver receptor signaling functionality in concert with metabolic competence reflective of inducible Zone-2 hepatic metabolism to predict adverse outcome pathways.

We present data characterizing perturbations on sentinel targets of cellular signaling pathways for 1060 chemicals, many of which occur in commerce and the environment. We further analyzed these data to identify patterns of transcription^58^ that are indicative of six different molecular initiating events and assess the probability of those events occurring as a function of concentrations for all the chemicals. This paper provides a novel Bayesian approach for integrating prior knowledge (results from other in vitro assays and literature mining) and new data (HepaRG^TM^ transcriptomic data) to predict putative molecular initiating events (receptor activation) for chemicals. Once the model is trained on reference chemicals, it can be used to predict (that is, infer) the targets and potency values for new chemicals. The combination of identifying a transcriptional signature and activation concentration are key to disentangling the complicated network of receptor crosstalk we observed (Fig. 6A).

If each transcript is a note, then each receptor plays a collection of notes—a chord. As concentration increases, each chemical varies in the progression of chords it plays by activating various receptors. By first identifying the chords corresponding to each of six receptors, we have begun to hear the songs of the ToxCast library. To identify MIEs we used a framework that allowed new transcriptomics data to be combined with gene-receptor relationships identified with literature mining. This resulted in predicted probabilities of interaction for the 1060 chemical library and each of the six receptors. Only 30% of the chemical library showed greater than 50% probability of interaction with any receptor below 100 µM, but many of those compounds potentially are activators for multiple receptors. We have observed that the proportion of active chemicals in the library is lower in this study than in previous ToxCast HTS data sets. It could be that the metabolic competence of the HepaRG^TM^ cell culture attenuates activity overall. Furthermore, it is possible that by analyzing the 32 genes and six receptors using a mechanistic, albeit simple, network model we increased the signal-to-noise ratio of the assay; that is, the receptor inferences are more statistically specific and less likely to identify a false positive. However, this specificity might have been offset by a large degree of crosstalk that was inferred between the six receptors. Cytoxicity at higher concentrations is known to cause a “burst” of signaling^55^ that in this analysis was set aside as noise, but future analyses could attempt to identify specific signatures of cytotoxicity (that is, cytotoxicity as a pseudo-receptor). Perhaps additional receptors, transcripts, and/or additional reference compounds could disambiguate these linkages, but by virtue of having performed a Bayesian analysis we know that this crosstalk is a plausible explanation for the prior literature information, this data set, and signaling model we have analyzed.

The MIE inference analysis allows this set of experiments to be interpreted as an IATA-like approach^67^. IATA provides practical, science-based approaches for chemical hazard characterization using an integrated analysis of all existing information combined with new data from novel assay strategies. Of course, while the homogeneity of the HepaRG^TM^ cell line allows for reproducibility, it eliminates the ability to assess human variability such as sensitivity populations^27^ and sex differences^68^.

Within this study a primary focus was to establish high quality HepaRG^TM^ cultures appropriately differentiated to provide metabolic competence and model liver enzyme induction. This was confirmed through cell morphology observations, baseline gene expression levels, and functional responses to prototype human hepatic receptor activators as described previously^69^. Additional lines of evidence such as aflatoxin B1 cytotoxicity, which is known to be dependent on CYP3A metabolism, and CYP3A4 induction in response to omeprazole further confirmed that the differentiation state of the HepaRG^TM^ cultures used here that was analogous to primary human hepatocyte cultures.

HepaRG^TM^ cell-cultures were selected because they overcome the challenges posed by de-differentiated HepG2 and primary human hepatocyte culture models. The hepatoma-derived HepG2 cell-line lacks native expression levels and functionality for various nuclear receptor signaling pathways, as has been reported and reviewed previously^52^. Primary human hepatocytes are a viable option, and initial ToxCast assays using that model were published in 2010^25^. However, high donor-to-donor variability and a finite supply of primary liver cells from a given donor preparation impair large chemical screening studies and year-over-year comparisons within a consistent genetic background. Pooling primary hepatocytes has recently become an option for short-term drug metabolism studies, but these technologies have not achieved sufficient quality in adherent culture configurations to model physiologically-relevant hepatocyte receptor signaling. HepaRG^TM^ have been previously found to model numerous hallmarks of hepatocyte functionality that includes CYP450 drug metabolism, Phase 2 metabolism, biliary network formation, active uptake and efflux transporter function, and nuclear receptor signaling functionality that includes proficiency to express and translocate the nuclear receptor CAR^69^.

A recent transcriptomics based analysis using global gene expression profiling showed that HepaRG^TM^ cultures outperformed human induced pluripotent stem cells (iPCSs) and other humans hepatoma cell lines (huH-7, HepG2, and HepG2/C3A) in their relation to human primary cells expression of hepatotoxicity related genes, drug metabolizing enzymes and transporters^27^. Similar to the results of Gerets et al.^26^ that used reference CYP450 inducers to deem HepaRG^TM^ cultures suitable for screening chemicals, our results showed evidence for metabolic capacity, albeit likely not at physiological rates.

Since this screen used an upper testing concentration of 100 µM, and the cell cultures were only exposed for 48 h, we may have been unable to achieve sufficient levels of metabolites to fully characterize the effects of metabolic activation/detoxification. However, the primary focus of this study focused on modeling chemical-induced effects on hepatic receptor pathways reflected in hepatic Zone-2 hepatocytes. Given the kinetics of enzyme induction vary between transcripts^51^, the 48 h time point used here may have also impacted responses for some transcripts. For example, omeprazole is very fast to induce CYP1A1 (6 h^25^), slower to induce CYP1A2 (24 h^70^), and even slower to induce CYP3A4 to near-maximal levels by 48 h^71^. As shown in Fig. 2 we observe induction by omeprazole of all three transcripts. Previous studies^50,69,72^ have demonstrated that 48 h exposures were effective to capture both rapidly responsive transcripts and slower responsive genes. Our intention was to allow additional time in response to chemical exposures to enable some formation of metabolites (for example, omeprazole, aflatoxin B1) and evaluate the combined effects of parent chemical and metabolites produced within this model system. It is certainly true that some transient responses related to stress responses (for example, oxidative stress) may be underrepresented using a single, later time point.

Metabolic transformation is thought to play a significant role in chemical induced-liver toxicity. This can result in both potentiation and attenuation of liver injury. In this study, the assay medium used was, by design, established to model Zone-2 hepatocyte metabolism to allow induction of major cytochromes P450 enzymes^73^. Therefore, the metabolic activity of the cell culture, while clearly present, was likely ~10% of human liver and suspensions of primary human hepatocytes comprised of both Zone-2 and Zone-3 hepatocytes from human liver^69^. This may have limited our ability to identify substantial differences in cytotoxic potency and efficacy between these metabolically competent assays (that is, LDH leakage) and the range of cytotoxicities reported in historical ToxCast assays (for example, ATP depletion) lacking metabolic capacity. It is further possible that we do not observe significant cytotoxicity due to effective induction of Phase I and II metabolism activities preventing their toxic action. Since metabolites are often structurally like parent chemicals, the success of QSARs and machine learning for predicting biological effect from structure perhaps suggests a reduced role for extensive metabolism for these chemical classes^74–76^. Finally, it is important to emphasize that liver injury and toxicity are far more complex than simply cellular death. Slower developing responses that manifest at the gene expression level after 48 h exposures are far better indicators of the path to liver injury often requiring weeks of exposure to manifest pathological changes (for example, steatosis, hepatocellular injury, fibrosis). Cytotoxicity responses for environmental exposures are unlikely to be more than a point of context to interpret more toxicologically-informative assay responses, but are likely more akin to LD50 determinations in guideline rodent toxicology studies.

An initial 93 transcripts were selected by expert opinion of the authors as being likely to inform hepatic xenobiotic signaling and pathways of toxicity. However, our inference model of receptor activation made use of only 32 of the transcripts. We anticipate that many more of the measured transcripts will be useful for other applications, such as the development of more accurate predictions of in vivo effects to screen for chemicals with potential public health effects. It is possible that using a wider range of transcripts and more sophisticated analysis of signaling networks may better inform prediction of toxic outcomes beyond identifying molecular initiating events. However, we acknowledge that in vitro cell cultures may lack compensatory abilities that are expected to be present in vivo^77^. Hussain et al.^78^ has shown that high-content imaging of HepaRG^TM^ cell cultures can predict human in vivo hepatic toxicity for drugs and other chemicals; however, Gerets et al.^26^ found that the frank cytotoxicity within HepaRG^TM^ cultures was less sensitive than primary human hepatocytes for detecting known hepatotoxicants. Gerets et al.^26^ did demonstrate that HepaRG^TM^ were more sensitive than the HepG2 carcinoma cell-line.

We did not observe activity for most chemicals. Since the ToxCast library represents chemicals that may occur in the environment, and though some are pesticides and pharmaceuticals included as points of reference, one might hope that most of these chemicals do not have significant bioactivity at sub-cytotoxic concentrations. As part of ToxCast program the chemicals screened here have also been screened in other relevant assays. While we did not observe as frequent activity as other ToxCast screens, we believe that the synthesis of multiple transcript activities into signatures that must be consistently observed reduces the likelihood of false positives. The ToxCast Factorial assay uses modified HepG2 cells to identify chemical perturbations of many transcription factors, including CAR, PXR, PPARα, FXR, and AR^79^. For AR, the assays agreed on eight chemicals and the Bayesian transcriptomics model assigned an average probability of 75% for AR interaction to those chemicals. The Bayesian transcriptomics model further identified 29 chemicals not found with the ToxCast Factorial assay, but the average probability for those chemicals was only 8.5%. The transcriptomics model assigned 0% probability to 14 chemicals identified as AR regulators by the ToxCast Factorial assay. For PPARα, the transcriptomic model identified 28 chemicals that also indicated PPARα activity with ToxCast Factorial, with an average probability of 61%. The transcriptomics model further identified 45 chemicals not identified by the ToxCast Factorial assay that had an average probability of 32%, while the Factorial assay found 52 chemicals not identified with transcriptomics. For PXR the transcriptomics model agreed with the Factorial assay on 131 chemicals (mean probability 77%), identified an additional 91 chemicals with a mean probability of 71%, and assigned 0% probability to 187 chemicals identified with the Factorial assay. There was even less agreement for FXR, where the two assays agreed on 15 chemicals as potential agonists (mean probability 73%), the transcriptomics model identified an additional 130 with a mean probability of 70%, and 40 chemicals identified by the Factorial assay were assigned 0%. The assays were most divergent for CAR, in which the Factorial assay agreed on only four chemicals (mean probability 1.3%), the transcriptomics identified an additional 330 chemicals (mean probability 44%), and the transcriptomics assigned 0% probability to 15 chemicals identified by the Factorial assay as perturbing CAR.

The data and analysis presented here can be used to refine existing predictions of molecular responses initiated by chemicals in the environment. In Judson et al.^11^ and Kleinstreuer et al.^12^ ToxCast assays were integrated into a predictive model for AR agonism. Of the chemicals in both studies, 68 were identified as potential AR agonists by Kleinstreuer et al., albeit in some cases weakly. Of those 68, the Bayesian analysis of the pattern of transcriptomic activities estimated nine chemicals to have some probability (mean 67.5%) of AR activation at less than 100 µM. Of the 848 chemicals not predicted to be AR agonists by Kleinstreuer et al., only 28 were estimated here to have any chance (mean 8.5%) to cause AR activation. In addition, 59 chemicals identified as AR agonists by Kleinstreuer et al. were assigned 0% probability of AR activation by the transcriptomics model. We hypothesize that in this study the synthesis of multiple transcript activities into signatures that must be consistently observed to infer activity decreases the number of potential false positives.

Transcriptomics provides a high-throughput approach to more comprehensively cover toxicity pathways in a high-throughput format and is being extensively evaluated by the U.S. EPA^15,32^. While the initial ToxCast assay suite has been able to provide more than a thousand assay endpoints, the overall coverage of pathways (and therefore potential toxic effects) has been limited^32^. Sentinel gene sets (for example, the L1000^80^) and whole-genome arrays with >23,000 transcripts should allow much greater insight into the biological effects of chemicals in the environment. In the interim, we present here an analysis of 93 genes limited to a single time point. Nevertheless, this represents an advancement for the field. Previously, in Rotroff et al.^25^, the gene expression changes induced by the activity of five receptors were studied in 14 genes for 309 chemicals from the ToxCast library as tested in eight-point concentration response for two human donors. That experiment produced roughly 70,000 data points for analysis. This work has yielded roughly 1.5 million data points. As whole genome arrays become amenable to screening mode use, even a single chemical might generate a third of a million data points with the same eight concentration, two replicate study design. Thus, bioinformatic challenges will continue to be at the forefront screening-mode transcriptomics^32^.

There are multiple points in an analysis at which Bayesian ideas may be incorporated. Both this analysis and the more typical *Bayesian network models*^81^ use prior information and probabilistic models to describe inference of genetic regulation. However, here we have determined the solution using a fully *Bayesian hierarchical approach* in which MCMC is used to estimate the full multivariate posterior distributions of parameters such as the receptor-gene interactions strengths^82^. This stands in contrast to Bayesian network models which use alternatives to MCMC that approximately identify the most likely networks^81,83^. Bayesian network models have successfully analyzed networks of tens of thousands of genes^84^, however, even those models can only approximate large-scale networks^85,86^. In part by incorporating the occurrence of gene-receptor pairs in the peer-reviewed scientific literature as prior information of a possible interaction, the fully Bayesian method used here was able to handle the combinatorics of teasing out quantitative genetic regulatory network interactions for specific receptors. This method is anticipated to scale in computation time roughly linearly with the number of genes and number of observations.

The method described here achieves full Bayesian posterior estimates by enumerating all the possible individual states of the receptors analyzed using a Boolean notation (from 000000 for all receptors inactive to 111111 for all active). Thus, we only need work with $2^6 = 64$ possible states, and all gene profiles possible with our model can be easily calculated using matrix multiplication of a matrix of *N* genes × six receptor weights and a six × 64 matrix mapping the 64 possible states back to individual receptor states. Using parallel MCMC on a relatively modern, multicore machine (at least 16 2.3 GHz processors) the analysis of the 32 gene, 1053 chemical data set takes roughly one week. Therefore, a whole genome analysis using this method might take years without further parallelization or increases in processor speeds. However, the receptor inference for non-reference chemical portion of the analysis (Step 3 in the “Methods” section and Fig. 4) is embarrassingly parallel. Therefore, computer systems with large numbers of parallel cores (for example, graphical processing units) might be able to greatly reduce the running time of the analysis. Further, improvements in computing speed might eventually make the analysis of whole genome microarrays feasible with this approach. The same is unfortunately not true for increasing the number of receptors (or other upstream factors) being inferred from the gene expression data. Although a moderate number of additional receptors might be included, the fact that the size of the analysis (as implemented) grows exponentially with the number of receptors precludes this analysis method for large numbers of receptors in a single model.

There remain thousands of untested chemicals in commerce and the environment. Transcriptomics with metabolically-competent in vitro models presents an opportunity for more thorough, accurate screening of these chemicals for prioritizing public health research. The methods described here use a cell line with relevant physiological response to xenobiotic chemicals recognition and regulation of metabolic enzymes in a concentration-response manner to identify putative MIEs for receptors as a steppingstone toward more quantitative AOP-based toxicological research.

## Methods

### Chemical library

The chemical inventories used in this study were the ToxCast Phase I and Phase II libraries; the “phases” here indicate order of testing by the ToxCast program and are unrelated to phases of metabolism^46^. There were 1060 unique test chemicals, to which one cytotoxicity reference compound and three receptor activator reference compounds were added. The total chemical list included ten chemicals designated as reference activators (17-Methyltestosterone, chenodeoxycholic acid, dichlorodiphenyltrichloroethane, fenofibric acid, methoxychlor, o,p'-DDT, omeprazole, phenobarbital, pirinixic acid, and rifampicin), as well as a cytotoxicity reference chemical (aflatoxin B1). The ToxCast Phase I library is primarily conventional pesticide active compounds, whereas ToxCast Phase II is more diverse environmental chemicals. The full list of chemicals is provided as supplementary material (see Supplementary Table 7). Chemical samples were commercially procured, diluted in 100% DMSO to a stock concentration of 20 mM and plated by Evotec (South San Francisco, CA). Analytical QC for the Phase I chemical inventory was performed using high-throughput liquid and/or gas chromatography mass spectrometry to determine sample purity, parent mass, and sample stability in DMSO over time (https://www.epa.gov/chemical-research/toxcast-chemicals). Similar methods were applied to analyzing the Phase II library in association with the Tox21 project and are publicly available at https://tripod.nih.gov/tox21/samples.

### Cell culture

Cell culture and cytotoxicity assays were conducted at Thermo Fisher Scientific’s Custom Services facility (Madison, WI). A single lot of human HepaRG^TM^ cells (Thermo Fisher Scientific, Frederick, MD) was used for the cell culture experiments. 96-well format collagen type I coated plates of human HepaRG^TM^ cells were prepared using established methods. Briefly, cryopreserved HepaRG^TM^ cells were thawed, plated at a density of approximately 100,000 cells/well in HepaRG^TM^ Thaw, Plate, and General Purpose media (HPRG770, Thermo Fisher Scientific), and incubated for 48 h. Test chemical plate layout is shown by Supplementary Fig. 4.

0.5% DMSO was selected as vehicle control to balance the requisite need of DMSO in HepaRG^TM^ cultures to maintain differentiation (for example, baseline metabolism, proportions of hepatocytes vs. cholangiocytes), while enabling further hepatic receptor pathway activation (for example, PXR and CAR) in response to chemical exposures^69^.

### Chemical exposure

Forty-eight hours after plating the culture medium was changed to HepaRG^TM^ Serum-Free Induction Media (HPRG750 Thermo Fisher Scientific). Cells were exposed with each test chemical in duplicate. Plates were returned to the CO_2_ incubators and maintained for 48 h until harvest.

Prior to exposing cells, chemical plates were thawed to room temperature and 200× concentrations of each treatment group were prepared. Eight-point concentration-response curves were prepared for each test compound with the highest exposure at 100 µM. The subsequent seven exposures were half-log dilutions, each with a final 0.5% DMSO concentration. For the positive control only, a separate preparation of phenobarbital was made such that the maximum concentration was 500 µM at 0.5% DMSO. As shown by Supplementary Fig. 4, each test plate contained induction positive controls (1 mM phenobarbital in water, *n* = 3 wells), cytotoxicity positive controls (100 mM aflatoxin B1, *n* = 3), vehicle controls (0.5% DMSO, *n* = 4), and total lysis controls (also 0.5% DMSO, *n* = 2).

A “reference plate” with two replicates each, of four of the 10 positive control inducers (phenobarbital, omeprazole, fenofibric acid, and chenodeoxycholic acid), was tested a total of six times across the screening process (Supplementary Fig. 2). The reference plate included a fifth chemical, aflatoxin B1, that was included to demonstrate metabolism-mediated bioactivity and related cytotoxicity. The reference plate also contained 0.5% DMSO vehicle control wells (*n* = 4) and total lysis control wells (*n* = 2). Cell morphology images were acquired for each well/plate with an Essen IncuCyte^TM^ FLR automated phase-contrast microscope located inside a tissue culture incubator. Six 96-well culture plates were loaded into the instrument and imaged for an elapsed time (~24 min). The IncuCyte^TM^ software was used for image capturing and export of images in JPEG format.

For cytotoxicity assessments, 50 µL of spent culture media from each plate was transferred into black-wall plates for the LDH assay. The balance of spent culture media (50 µL) was transferred to fresh polypropylene plates and sealed/stored at −80 °C. The cells were treated with 75 µL of RLT buffer (Qiagen) and frozen at −80 °C until shipped to Quintiles/Expression Analysis (Durham, North Carolina) for gene expression analysis.

### Cytotoxicity assay

The CytoTox-ONE^TM^ Homogeneous Membrane Integrity Assay (Promega, Madison WI) was used to measure the LDH leakage activity as a measure of membrane integrity and cytotoxicity in the cells. For this assay, 50 µL of spent culture media was removed from each well/plate and transferred to black-walled 96-well assay plates. Triton X (0.1%, 100 µL) was added to the two total lysis control wells on each plate (thereby releasing the maximum amount of LDH into the supernatant) and briefly mixed by pipetting to generate total lysis controls for each plate. The presence of LDH was measured by addition of the resazurin, which is converted to the fluorescent chemical resorufin in a reaction coupled to LDH enzymatic activity. Fluorescent emission was detected using a Safire^2^ microplate reader (Tecan).

### Cytotoxicity data analysis

LDH assay results generated by Life Technologies (Madison, WI) and analyzed with R (v3.6.1)^87^ using the ToxCast pipeline package R (tcpl) for curve fitting^38^. Data were converted from raw relative fluorescence units (RFU) to percent cytotoxicity and normalized to the vehicle and total lysis controls on each plate, as follows^88^:

1 $${\mathrm{Percent}}\;{\mathrm{cytotoxicity}} = 100 \times \frac{{{\mathrm{Experimental}} - {\mathrm{Culture}}\;{\mathrm{Medium}}\;{\mathrm{Background}}}}{{{\mathrm{Maximum}}\;{\mathrm{LDH}}\;{\mathrm{Release}} - {\mathrm{Culture}}\;{\mathrm{Medium}}\;{\mathrm{Background}}}}$$

The percent cytotoxicity normalized to vehicle control (DMSO) were the response values (rval) analyzed using the same three models and criteria described below for the transcriptomics data analysis. Based on visual inspection of the cell morphology images for the positive control, aflatoxin-B1 cytotoxicity was determined to be at 12% of the observed total lysis reading. Based upon the relatively high (for ToxCast) signal to noise ratio of this assay, a criterion of exceeding 10 times the baseline median average deviation (BMAD) was used to determine a “hit”. BMAD is defined as the median average deviation of all normalized response values at the lowest two tested concentrations. Supplementary File LTEA_Inucyte_Images.zip is comprised of 20,493 images totaling more than 15 gigabytes, as such these data are only available via File Transfer Protocol (FTP): ftp://newftp.epa.gov/COMPTOX/CCTE_Publication_Data/CCED_Publication_Data/Wambaugh/ToxCast_LTEA as file LTEA_Inucyte_Images.zip. The LDH data are available in Supplementary Table 8.

### Transcriptomics assay

Total RNA was isolated from the cell suspension in 75 µL of Buffer RLT (Qiagen, Germantown, MD 20874) by Quintiles/Expression Analysis (Durham, NC) using a custom isolation procedure. Total RNA from each plate well was transferred to a preamplification plate. Fluidigm’s 96.96 Dynamic Array^TM^ (Fluidigm, Amsterdam, the Netherlands) technology was used for gene expression analyses by quantitative reverse transcription polymerase chain reaction (qRT-PCR, performed by Quintiles/Expression Analysis, Durham, NC)^89^. Standard TaqMan^TM^ Assays (Applied Biosystems/ThermoFisher Scientific, Foster City, CA) were used to assess the expression of 93 genes selected based on their selectivity and sensitivity to important hepatic receptors known to be modulated by environmental chemicals; importance in human hepatocyte functionality; and hepatotoxicity. Three “housekeeping” endogenous control genes were also included and used for normalization: actin β (ACTB), Glyceraldehyde 3-phosphate dehydrogenase (GAPDH) and RNA Polymerase II Subunit A (POLR2A). qRT-PCR was conducted according to the manufacturer’s protocol. The transcripts tested and TaqMan^TM^ probes used are further described in Supplementary Table 1 and Supplementary Table 9.

### Transcriptomics data analysis

Gene expression data from the Fluidigm qRT-PCR arrays was analyzed in R. Prior to processing through the tcpl package, each qRT-PCR primer set was annotated as an individual assay endpoint (aeid) for analyses. For each plate, well types were designated for test compound wells (*t*), positive controls (*c*), (that is phenobarbital) and neutral controls (*n*, DMSO). Fold-change in the number of amplification cycles needed to pass the background threshold (Ct) for 96 transcripts to (ftp://newftp.epa.gov/COMPTOX/CCTE_Publication_Data/CCED_Publication_Data/Wambaugh/ToxCast_LTEA, file LTEA_Level2_20191119.zip) were normalized to the geometric mean of three housekeeping genes (ACTB, GAPDH, POLR2A) to generate ΔCt values (cval). Prior to calculating the response values (rval), or ΔΔCt, for each transcript (*n* = 96) per well, the baseline value (bval), the plate-wise median of the neutral control wells, was generated for each plate (the normalization process is described in detail in the Supplementary Methods (“DeltaCT Calculations”). The bval was subtracted from the cval to yield the rval or log2 Fold Change per transcript.

Curve fitting was then performed using the R package tcpl. The concentration-response based rval data for each chemical-gene combination were fit to three models: constant, a Hill, and gain-loss model (the non-monotonic product of two Hill functions with a shared top) on a per chemical per transcript basis. Each model fit was compared using an Akaike Information Criterion (AIC) value and the model with the lowest AIC was selected as the winning model. Curve parameters from the winning model such as the half-maximal activity value (50% activity concentration; AC_50_; modl_ga) and the modeled top of the curve (modl_tp) were used to quantify potency and efficacy, respectively. The chemical was identified as a “hit” (that is, significantly change in gene expression) by exceeding either three times the BMAD (typical of ToxCast assays) or a 1.2-fold-change cut-off at any concentration. Curve fits are available from the CompTox Chemicals Dashboard: https://comptox.epa.gov/dashboard.

### Receptor-gene literature mining

Using literature co-occurrence is an established methodology for identifying information about genes^90,91^. We used literature mining to identify which of the 93 target genes have known relationships to members of the set of nuclear and other receptors. Literature mining was performed on our database of MeSH annotations extracted from articles in PubMed^92^. The database was queried for the MeSH terms identifying the genes and receptors of interest (run June 2017). To identify this initial set of gene search terms, the MeSH term or supplemental concept term that reflected the gene most closely was chosen using the MeSH browser (https://meshb.nlm.nih.gov) and searching on either the gene symbol or gene/protein name. The database query using the MeSH search terms returned PubMed identifiers for each article in which a gene of interest was annotated with a receptor. We counted the number of times each gene and receptor co-occurred in the MeSH terms for an article (Supplementary Table 10 and Supplementary Table 11). These article co-occurrence counts were assumed to be a function of the same receptor-gene interaction network that generated the gene expression data and were used as input into the Bayesian analysis below. This approach assumed that receptors behave the same in different cell types and tissues.

### Model for receptor-regulated gene expression

We assumed a model in which activation of a receptor causes the up or downregulation of some genes to be more likely, while leaving other genes unchanged. The ten reference activator chemicals provided a training set indicating which genes change expression level when a specific receptor is activated. Genes that were not found to be regulated by any of the six receptors were omitted from the analysis. A stochastic framework was used such that activation of a receptor only made changes in expression level more or less likely; since the model is not deterministic, there is always the possibility of gene expression remaining unchanged despite signaling from receptors. Patterns of gene expression changes were evaluated for each test chemical and tested concentration for consistency with the patterns observed for known activators of six different receptors.

This statistical model uses gene expression data across all concentrations simultaneously. At a given concentration, the receptor state of a chemical is described by a Boolean vector with a 0/1 for each receptor, with 1 indicating activation. For each receptor and chemical combination, there was a specific concentration above which the receptor was activated; here the AC_50_(receptor, chemical) from the curve fits was used as a surrogate for this potency. For *Z* chemicals, the AC_50_ matrix contains *Z* × 6 potencies. For each chemical-receptor pair the receptor state is always zero (inactive) below the AC_50_ and is always 1 (active) above the AC_50_. When the AC_50_ is above the highest measured concentration, this is equivalent to the chemical not activating the receptor in the tested range. By interpreting the state of the six receptors as a binary number (six ones and zeros) a unique integer could be calculated to represent the states for all receptors for the each chemical and concentration tested, $S\left( {{\mathrm{chemical}}_j,{\mathrm{concentration}}_k} \right) \in \left[ {0,2^6 - 1} \right]$, this is *Z* × 1 matrix that is a function of chemical concentration *C*. Since there were six binary receptors, there were only 2^6^ = 64 possible values of *S* across all the observations. While it would be possible to model a repressed state for the receptors, the number of states would be 3^6^ = 729. Further, we did not have a reference plate of antagonist chemicals for training the model.

The state for each of the receptors can impact the state (up/down/unchanged) for the transcripts (*N*, initially 93). The impact of activating a receptor is assumed to be the same regardless of the chemical causing the activation. For each receptor, unbounded weights for each of the *N* transcripts (a vector of *N* weights) were estimated; these weights could be positive (causing upregulation) or negative (downregulation). A Horseshoe prior^93^ constrained the estimated weights to be sparse (mostly zero, such that most receptors do not affect the expression of most genes). The weights for all genes and all receptors composed a *N* × 6 matrix, **M**. **M** is independent of the test chemicals and describes the relationship between genes and receptors for the cells.

For faster computation a 6 × 64 matrix **B** was constructed to contain all permutations of receptor activation (that is, all 64 possible values of *S* ranging from 0,0,0,0,0,0 to 1,1,1,1,1,1). The matrix **M** was multiplied by **B** to produce a *N* × 64 matrix, **W**. The probability matrix **P** for gene expression was then calculated by indexing into **W**, **P**_*j,k*_ = **W**(*S*(*j,k*)) where *j* indicates chemical and *k* indicates concentration. **P** was separated into **P**^***up***^ = **P**_***ij***_ when **P**_*ij*_ > 0.0 otherwise and **P**^*down*^ = |**P**_***ij***_|when **P**_***ij***_ < 0 and 0 otherwise such that **P** = **P**^*up*^−**P**^*down*^. Probability of activation was calculated according to a Dirichlet distribution parameterized with a three-element vector (basal, upregulated, downregulated) where the values for each of the three categories indicated the un-normalized weight for the probability of each state:

2 $${\mathrm{Observation}}\left( {{\mathrm{gene}}_i,{\mathrm{chemical}}_j,{\mathrm{concentration}}_k} \right)\sim {\mathrm{Prob}}_{i,j,k} = {\mathrm{Dirichlet}}\left( {\theta _i,P_{{\mathrm{up}},i,j,k},P_{{\mathrm{down}},i,j,k}} \right)$$

**θ**_*i*_ represents a gene-specific activation threshold or “stiffness” indicating how much more or less likely that gene is to respond to receptor signaling.

### Basis for parameter prior probability distributions

Bayesian analysis combines information from new data with probability distributions representing the “prior” information that can be either assumed or drawn from previous studies^94^. Here there were two priors used, one on chemical-receptor interactions for the ten reference activator chemicals (Supplementary Table 12) and a second prior for receptor-gene interactions reflecting the scientific literature. In steps one and two of the Bayesian analysis a Horseshoe prior was used for Gene-Receptor interactions which acted to make most interaction weights small (no interaction) unless the data indicated otherwise.

### Chemical-receptor priors

Six receptors were examined in this study (AR, AhR, PXR, CAR, PPARα, FXR). A combination of ToxCast and Tox21 assay results were used to characterize receptor activity (Supplementary Table 12). Ten activator reference chemicals were identified, each having known activity for one or more of the six receptors under study.

Because chemical partitioning is not measured in HTS in vitro assays and actual cell exposure is unknown^95,96^, literature data on potency information was discarded and a chemical that was a positive control for a receptor was simply assumed to be active at some tested concentration (potency information is available in Supplementary Table 12). A receptor-chemical pair with no observed activity was modeled as if activity might still occur, but at a concentration higher than tested. If there was no receptor-specific assay data available for a chemical, all concentrations modeled (all tested plus one concentration above the maximum tested) were considered equally likely.

### Receptor-gene priors

Output from literature mining was used as a second set of prior data in analysis steps one and two (see Supplementary Table 10 and Supplementary Table 11). Each occurrence of an abstract that mentioned both a receptor and gene under study was treated as weak evidence of a non-zero interaction between that receptor and gene. Statistically, each abstract was assumed to be an independent observation that the interaction between a receptor *i* and gene *j* was non-zero. A censored distribution was used such that the absolute value of the weight, |*M*_*i,j*_| was greater than a threshold, µ, given normally distributed measurement error. A single threshold value µ and standard deviation were estimated across all abstracts.

### Procedure for Bayesian analysis

JAGS v4.2 (http://mcmc-jags.sourceforge.net/)^97^ was used to perform Markov Chain Monte Carlo^98,99^ because of its ability to sample from the discrete probability distributions necessitated by our simplification of the concentration-response data (basal, up, down). JAGS was automated through R using the package runjags^100^.

The transcription data were fit in positive and negative modes (increase or decrease of gene expression over control). These data were discretized in two ways: first, only three states–basal, upregulated, or downregulated were considered. Second, if the data for a chemical-gene pair were found to be well-described by concentration-response function (a “hit”), then the observations were recorded as basal for all tested concentrations below the AC_50_, and as either “up” or “down” for all concentrations above the AC_50_. Chemicals where no systematic response was observed were treated as “basal” for all tested concentrations.

Due to the computational complexity of the problem, Bayesian analysis proceeded in three steps (Fig. 4). First, the 93 genes were reduced to the most informative subset based on analyzing one receptor at a time using the reference chemical transcriptional data and the literature mining evidence. Second, the model was retrained on the all six receptors jointly, using the reference chemicals and literature mining evidence on the reduced subset of genes. Third, the model was applied to all test chemicals using the refined signaling network learned from reference chemicals. At each step, multiple chains were run using runjags^100^ until each chain had burned in and passed the Heidelberger and Welch test^101^ for autocorrelation and the ensemble of chains had passed the Gelman–Rubin test^102^ for mixing. The three steps were:

1. Feature selection. This step uses the reference chemical-receptor and receptor-gene priors described above. Gene expression data were analyzed for all genes but for only the reference chemicals. Expression is analyzed as a function of each receptor separately (univariate). The 93 assayed genes are subdivided to only those *N*_*i*_*’* genes that showed activity (up or down) for some concentration for at least one of the chemicals indicated to have activity for receptor *i*. In each univariate analysis a single pattern of receptor-mediated transcriptional activity was identified. Each reference chemical for the receptor *i* is assumed to switch on that activity at a chemical-specific concentration, *AC*_50_*(receptor, chemical)*, that was inferred from these data. Six receptor-gene matrices **M** were estimated. For a transcript to be included in the second step, there had to be at least 50% confidence that the receptor-gene weight *M*_*ij*_ was non-zero for at least one receptor. In this way, a co-regulated pattern of activity among *N*″_*i*_ ≤ *N*′_*i*_ transcripts was separated from random spikes in the data and the activity of other receptors.
2. Gene-receptor interaction estimate. Determined simultaneously (multivariate) for all six receptors. Again, this step used the reference chemical-receptor and receptor-gene priors described above. Only the *N*′ = U_*i*_*N*″_*i*_ transcripts that were univariately correlated with activation of a receptor for any reference chemical were included (that is, 95% credible interval did not include zero). All reference chemicals were analyzed jointly for activity induced by all six receptors, estimating a *N*″ × 6 matrix **M’** for receptor-gene interactions. All entries in **M’** that were close to zero (95% credible interval included zero) were set to zero in subsequent analysis.
3. New prior probability distribution for the non-zero entries of M’. A multivariate normal distribution, which allowed for correlation between parameters, was fit to the chains from step 2 for the non-zero entries of **M** and the activation weights for the different genes. In this way, the correlated, non-zero posterior estimates of **M’** from the second step became the prior for the third step. In the third step the model is built from the analysis of the reference chemical dataset was then used to analyze the remaining 1056 ToxCast chemicals. Both the values for **M’** and the activation concentrations for all six receptors for each chemical was estimated. A uniform prior was used on each tested concentration.

### Reporting summary

Further information on research design is available in the Nature Research Reporting Summary linked to this article.

## Supplementary information

Supplementary Material

Supplementary Tables

Reporting Summary

## Data Availability

Three large datasets generated during and/or analyzed during this study are freely, and publicly available from the U.S. Environmental Protection Agency at: ftp://newftp.epa.gov/COMPTOX/CCTE_Publication_Data/CCED_Publication_Data/Wambaugh/ToxCast_LTEA. These three data sets are files LTEA_Inucyte_Images.zip (images of each cell culture), LTEA_Level2_20191119.zip (the raw, unnormalized data), and LTEA_Level5_20191119.zip (results of concentration-response curve-fitting). All other data used this study are included in the Supplementary Tables.

## Code availability

The Bayesian analysis R scripts are available in the section “R Code” of the Supplementary Materials and the JAGS model files are available in the section “JAGS Code”. All other R work was performed using R packages available from the Comprehensive R Archive Network. Curve fitting using the “tcpl” package is described in detail in the Supplementary Methods section “DeltaCT Calculations”.
