# Supplementary material for: High-throughput toxicogenomic screening of chemicals in the environment using metabolically competent hepatic cell cultures
Source: NPJ Syst Biol Appl. 2021 Jan 27;7:7. doi: 10.1038/s41540-020-00166-2 (PMC7840683; doi:10.1038/s41540-020-00166-2)
Supplement: Supplementary file 1 — Supplementary Material [file 41540_2020_166_MOESM1_ESM.pdf]

Supplementary Materials for High-Throughput Toxicogenomic Screening of Chemicals in the Environment Using Metabolically Competent Hepatic Cell Cultures

Authors:
Jill A. Franzosa[1], Jessica A. Bonzo[2], John Jack[1], Nancy C. Baker[1], Parth Kothiya[1], Rafal P. Witek[2], Patrick Hurban[3], Stephen Siferd[3], Susan Hester[1], Imran Shah[1], Stephen S. Ferguson[4], Keith A. Houck[1], John F. Wambaugh[1,*]

[1]Center for Computational Toxicology and Exposure, Office of Research and Development, U.S. EPA, Research Triangle Park, NC 27711
[2]Cell Biology, Biosciences Division, Thermo Fisher Scientific
[3]Expression Analysis
[4]Division of National Toxicology Program, National Institutes of Environmental Health Sciences of National Institutes of Health, Durham, NC, USA, 27709.

*Corresponding Author:
109 T.W Alexander Dr., NC 27711, USA
Wambaugh.john@epa.gov
Phone: (919) 541-7641; fax: (919) 541-1194

Contents

Supplementary Figures 4
Supplementary Methods 8
DeltaCT Calculations (LTEA Data Analysis: Level 2-3 Normalization) 8
R Code for Bayesian Analysis 10
heparg-ref-step1-analysis-072017.R 10
heparg-ref-step2-analysis-112917.R 12
heparg-tox-analysis-042318.r 17
load-hepdata-121117.R 24
analyze-step1-112917.R 31
build-posterior-network-043018.r 34
create.model-042018.r 35
create-NR-prior-tables-033116.R 39
create-refchem-figure-052218.R 41
do.JAGS-090617.R 43
get-NRs-and-RefChems-070715.R 46
heatmap.3.r 47
myclust.R 57
plot.data-062915.R 58
plot_NR_graph_posterior-070615.R 60
plot-NR-AC50-prior-112415.R 62
plot-NR-inferrence-043018.r 63
toxcast["NOCAS_47351","PREFERRED.NAME"] <- "SSR 240612" 63
posterior-analysis-043018.r 65
prepare-JAGS-run-012916.R 68
read-literature-prior-072017.R 69
ToxCast-NRInferrence-figure-052218.R 70
JAGS Code for Bayesian Analysis 73
HepaRG-Ref-Step1-Jul17-2017-07-20.jags 73
HepaRG-Ref-Step2-Nov2017-2017-11-30.jags 75
HepaRG-ToxCast-Apr2018All-2018-04-23.jags 77

Other Supplementary Files:

SupplementaryTables.xlsx: A Microsoft Excel workbook containing many tables (“sheets”) referred to in the main text.

The following data sets are too large to provide as part of the publication are publicly available at ftp://newftp.epa.gov/COMPTOX/CCTE_Publication_Data/CCED_Publication_Data/Wambaugh/ToxCast_LTEA:

LTEA_Inucyte_Images.zip: The images of each well containing a HepaRG™ cell culture (15.8 GB)

LTEA_Level2_20191119.zip: LDH and transcriptional concentration-response fold-change data (37.9 MB)

LTEA_Level5_20191119.zip: The ToxCast curve fits of the transcriptomic and LDH data (30.5 MB)

All ToxCast data and curve fits may also be viewed at https://comptox.epa.gov/dashboard/

Supplementary Figures

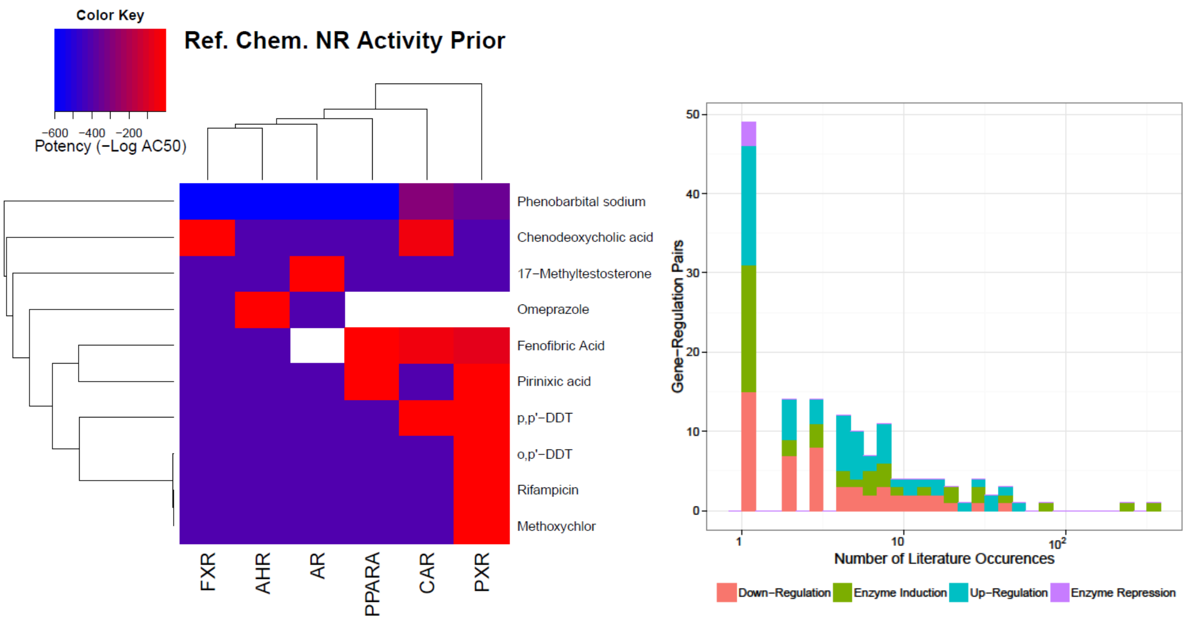

Supplementary Figure 1: A Bayesian model was used to infer receptor activation from a combination of the new transcriptional data and prior information: (at left) Curation of in vitro data identified receptor 50% activation concentrations (potencies) for the ten reference chemicals. Rows (chemicals) and columns (receptors) are hierarchically clustered according to Euclidean distance using complete linkage such that more similar rows and columns are near each other. Color in the heatmap indicates the potency, with white indicating instances where no data were available. (at right) Co-occuring mentions of receptors and transcripts were curated from the published literature. The histogram shows that most occurrences were of a single instance of a receptor and transcript being mentioned, while in some cases there were several hundred occurrences. The heatmap was created with R package ComplexHeatmap [97] using hierarchical clustering with complete linkage and an Euclidean distance.

Reference Plate

	1	2	3	4	5	6	7	8	9	10	11	12
A	PB.1	PB.1	OMP. 1	OMP. 1	FF.1	FF.1	CDCA. 1	CDCA. 1	AFL.1	AFL.1	DMSO	DMSO
B	PB.2	PB.2	OMP. 2	OMP. 2	FF.2	FF.2	CDCA. 2	CDCA. 2	AFL.2	AFL.2	DMSO	DMSO
C	PB.3	PB.3	OMP. 3	OMP. 3	FF.3	FF.3	CDCA. 3	CDCA. 3	AFL.3	AFL.3		Total lysate
D	PB.4	PB.4	OMP. 4	OMP. 4	FF.4	FF.4	CDCA. 4	CDCA. 4	AFL.4	AFL.4		Total lysate
E	PB.5	PB.5	OMP. 5	OMP. 5	FF.5	FF.5	CDCA. 5	CDCA. 5	AFL.5	AFL.5		EA
F	PB.6	PB.6	OMP. 6	OMP. 6	FF.6	FF.6	CDCA. 6	CDCA. 6	AFL.6	AFL.6		EA
G	PB.7	PB.7	OMP. 7	OMP. 7	FF.7	FF.7	CDCA. 7	CDCA. 7	AFL.7	AFL.7		EA
H	PB.8	PB.8	OMP. 8	OMP. 8	FF.8	FF.8	CDCA. 8	CDCA. 8	AFL.8	AFL.8		EA

No cells

CAR/PXR PB: Phenobarbital
AhR OMP: Omeprazole
PPARα FF: Fenofibric Acid
FXR CDCA: Chenodeoxycholic Acid
Toxicity AFL: Aflatoxin B1

DMSO: Vehicle control
Total Lysate: LDH assay control
EA: Empty wells for EA to add universal human reference RNA

Supplementary Figure 2: Seven reference chemical plates were interspersed throughout the experimental process. Reference chemical plates contained both reference chemicals for metabolic activity (aflatoxin b1) and reference receptor activators.

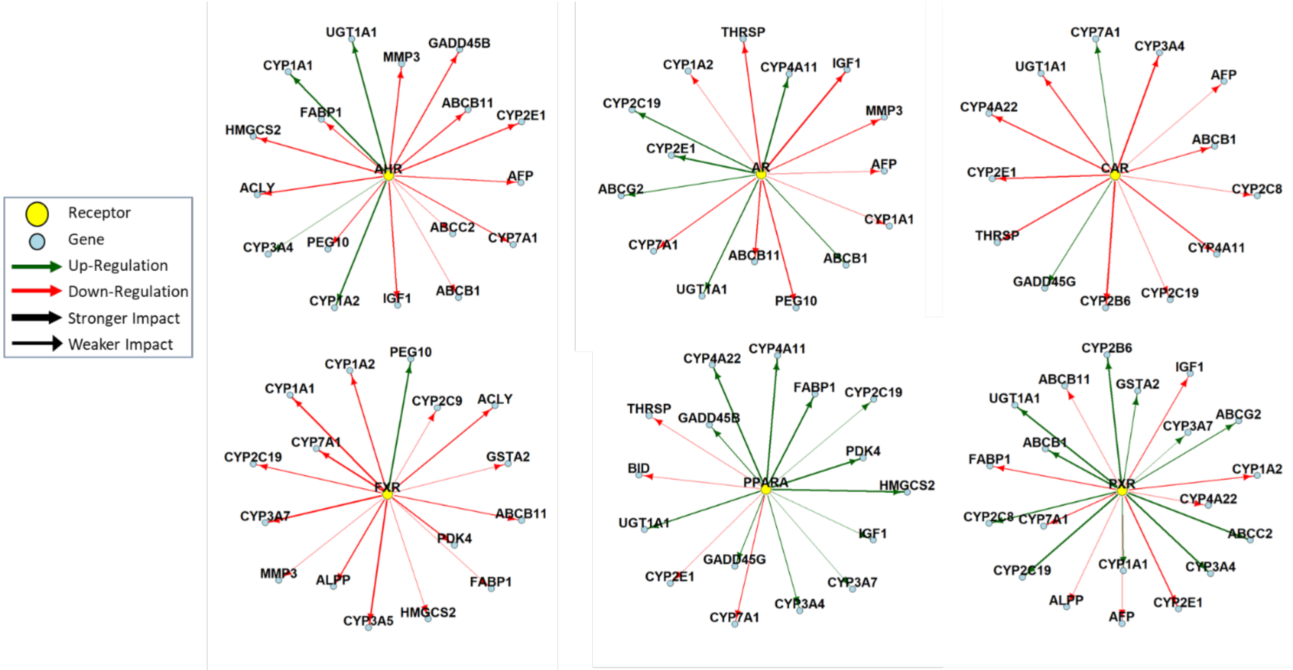

Supplementary Figure 3: Each network represents the direction and magnitude of interaction for the six receptors in HepaRG™ cultures as determined using the entire chemical library as a set of test perturbations.

Test Compound Plate

	1	2	3	4	5	6	7	8	9	10	11	12
A	PB.1	1.1	2.1	3.1	4.1	5.1	1.1	2.1	3.1	4.1	5.1	DMSO
B	PB.2	1.2	2.2	3.2	4.2	5.2	1.2	2.2	3.2	4.2	5.2	DMSO
C	PB.3	1.3	2.3	3.3	4.3	5.3	1.3	2.3	3.3	4.3	5.3	Total lysate
D	AFL.1	1.4	2.4	3.4	4.4	5.4	1.4	2.4	3.4	4.4	5.4	Total lysate
E	AFL.2	1.5	2.5	3.5	4.5	5.5	1.5	2.5	3.5	4.5	5.5	EA
F	AFL.3	1.6	2.6	3.6	4.6	5.6	1.6	2.6	3.6	4.6	5.6	EA
G	DMSO	1.7	2.7	3.7	4.7	5.7	1.7	2.7	3.7	4.7	5.7	EA
H	DMSO	1.8	2.8	3.8	4.8	5.8	1.8	2.8	3.8	4.8	5.8	EA

No cells

1.1, 2.1, 3.1, ..., 5.8 ToxCast compound N, dilution 1, 2, 3,...
PB Phenobarbital
DMSO Vehicle control
Total Lysate LDH assay control
AFL Aflatoxin B1
EA Empty wells for EA: one no template control, one no enzyme control, and two for universal human reference RNA

Supplementary Figure 4: Each test chemical plate contained duplicate eight-point dilutions of five ToxCast compounds.

Supplementary Methods

DeltaCT Calculations (LTEA Data Analysis: Level 2-3 Normalization)

We should have unique Ref Genes values for each well. All this data is in level2. In level3 we have the all the same ref.gene.mean values for an individual chemical treatment. The reference gene expression should be approximately the same per treatment and concentration, yet, there could be slight variations that we want to capture.

Here, perhaps we just want to take the mean of the housekeeping gene assays based on the sample_tech_rep_id (individual well). Example for TX011582_1:

1) Ref_GENE_Mean (TX011582_1) = Mean (Raw_ct_value(TX011582_1(ltea_act);Raw_ct_value _TX011582_1(ltea_gadh) ;Raw_ct_value (TX011582_1(ltea_polr2a))]
Calculating the delta Ct is the next step. This would involve subtracting the Ref_Gene_Mean for an sample_tech_rep_id (individual well) from every all of the assays for that sample_tech_rep_id

2) delta Ct (LTEA_abcb1) = Raw_ct_value(TX011582_1,LTEA_abcb1) – Ref_GENE_Mean (TX011582_1) –equation 1
We should also have unique DMSO means for each assay and plate combination in Level 3. The question here is did DMSO affect the expression of that specific gene (assay) on that plate? The data is all there in the plate level, LEVEL2 files. In Level 3 we have the same DMSO mean listed for all the plates and assays.

Here's an example equation for calculating the dmso_mean for a specific assay and plate:

1) delta_ct _DMSO$_{\text{(plateX, well X\#, LTEA_cyp3a4)}}$ =Raw_ct_value DMSO$_{\text{(plateX, well X\#, cyp3a4)}}$ – [Mean(Raw_ct_value DMSO$_{\text{(plateX, well X\#,,act)}}$;Raw_ct_value DMSO$_{\text{(plate1, well X\#,gadh)}}$;Raw_ct_value DMSO$_{\text{(plate1, well X\#,polr2a)}}$)]
*Will have four of the values (for each of the 4 DMSO wells) for each assay on an individual plate. The mean of these will be equation 2). Parth has them nicely labeled as DMSO_1 -4 on each sheet.

In the example above, sample_tech_rep_id can be substituted for wellX#

2) delta_ct_DMSO_mean$_{\text{(plateX,assay_cyp3a4)}}$ = Mean (delta_ct _DMSO_1$_{\text{(plateX, well X\#, assay_cyp3a4),}}$ delta_ct _DMSO_2$_{\text{(plateX, well X\#, assay_cyp3a4),}}$ delta_ct _DMSO_3$_{\text{(plateX, well X\#, assay_cyp3a4),}}$ delta_ct _DMSO_4$_{\text{(plateX, well X\#, assay_cyp3a4)}}$)

The DMSO is only labeled in the sample_rep_id column. Could this possibly be attributing to its processing from level 2 to 3? Would we want to include it in the chemical name, also?

Other descriptive data already in Level3 file should be included left

Sample_tech_rep_id	assay_name	Raw_Ct_value	House_keep_Mean_Ct	delta_Ct_value	delta_Ct_DMSO_Mean	delta_delta_Ct_value	Fold Change
TX#######_1	LTEA_assay	From Raw Data Files	= Mean of the three housekeeping gene assays (actb, gapdh, [illegible]	= (Raw_Ct_value) - (House_keep_Mean_Ct) [illegible]	See Below	= (delta_Ct_value) - (delta_Ct_DMSO_Mean)	= 2^-(delta_delta_Ct_val [illegible]

1. Calculate the delta_ct_DMSO_# for each of the 4 DMSO wells on a plate delta_Ct_DMSO_#= (Raw_Ct_value(DMSO_1,plateX,assay)) - (House_keep_Mean_Ct(DMSO_1,plateX)) – see above *Consider making a DMSO_PLATE_WELL ID similar to the sample_tech_rep_id for the DMSOs
2. Take the Mean of all **4** DMSOs well for that <u>specific assay</u> **delta_Ct_DMSO_Mean = Mean(delta_Ct_DMSO_1; delta_Ct_DMSO_2; delta_Ct_DMSO_3; delta_Ct_DMSO_4)**

*These will be gene/assay specific

Do we want to incorporate the intermediate values calculated into the Level3

R Code for Bayesian Analysis

heparg-ref-step1-analysis-072017.R

```
library(runjags)
library(gdata)
library(parallel)
library(gplots)
library(ggplot2)
library(scales)
library(data.table)

# Get rid of anything in the workspace:
rm(list=ls())

##########################################################################################
# CONFIGURATION VARIABLES ###############################################################

source("create.model-072017.R")

#Set the filename stub:
FILENAME <- "HepaRG-Ref-Step1-Jul17"

# The number of Markov Chains used:
NUM.CHAINS <- 6

#Set the name of the BUGS file:
RJAGS.MODEL.FILE <- paste(FILENAME,"-",Sys.Date(),".jags",sep="")

# For the initial analysis we look at all genes with reference chem activity:
RESTRICT.TO.SIGNIFICANT.GENES <- FALSE

# Name of the ToxCast Curve fit file
HEPARG.table <-"LTEA_Level5&6_20150922.csv"

# Literature activities of the reference chemicals:
NR.CHEM.PRIOR.TABLE <- "NR-prior-ToxCast Assay Data-071315.xlsx"

# We repeat the observaions from the reference plates in order to weight them
# appropriately (more observations should mean more weight)
NUM.REFERENCE.PLATES <- 7

#Are we analyzing the reference chems or the ToxCast library
REFERENCE.CHEMS <- TRUE

# Literature NR-Gene intractions:
```

```
LITERATURE.NR.GENE.INTERACTIONS<- "LitConnectionsForPriors_v3.xlsm"

# END CONFIGURATION VARIABLES ######################################################
######################################################################################

# Now read in the chemical-specific NR interaction prior information (Jill/John):
source("get-NRs-and-RefChems-070715.R")

# Create the hepdata table with the simplified LTEA data:
source("load-hepdata-072417.R")

repdata <- subset(hepdata,casn%in%c("474-25-9","57-30-7","73590-58-6","42017-89-0"))
for (i in 1:(NUM.REFERENCE.PLATES-1)) hepdata <- rbind(hepdata,repdata)

# We use these tables as arguments to the function that builds the JAGS model:
source("create-NR-prior-tables-032616.R")

# Read table of literature NR-gene interactions with direction (Nancy Baker):
source("read-literature-prior-072017.R")

# Create the BUGS file:
create.ref.model(RJAGS.MODEL.FILE,1)

save.image(paste(FILENAME,"-",Sys.Date(),"-preJAGS.RData",sep=""))

source("do.JAGS-090617.R")
cl1 <- makeCluster(Num.NR,outfile=paste(FILENAME,"-clusterlog",Sys.Date(),".txt",sep=""))
clusterExport(cl1,list("FILENAME","NUM.CHAINS"))
clusterEvalQ(cl=cl1,setwd("/Users/jwambaug/Desktop/heparg"))
clusterEvalQ(cl=cl1,source("/Users/jwambaug/Desktop/heparg/prepare-JAGS-run-012916.R"))
outlist <- clusterApply(cl1, NRs, do.JAGS)
save.image(paste(FILENAME,"-",Sys.Date(),".RData",sep=""))
clusterEvalQ(cl =cl1,stopCluster(cl2))
stopCluster(cl1)

# Get rid of anything in the workspace:
rm(list=ls())
```

heparg-ref-step2-analysis-112917.R

```
library(runjags)
library(gdata)
library(parallel)
library(gplots)
library(devtools)
library(ggplot2)
library(scales)

# Get rid of anything in the workspace:
rm(list=ls())

##########################################################################################
# CONFIGURATION VARIABLES ##############################################################

source("create.model-072017.R")

#Set the filename stub:
FILENAME <- "HepaRG-Ref-Step2-Nov2017"

# The number of Markov Chains used:
NUM.CHAINS <- 6

#Set the name of the BUGS file:
RJAGS.MODEL.FILE <- paste(FILENAME,"-",Sys.Date(),".jags",sep="")

# For the initial analysis we look at all genes with reference chem activity:
RESTRICT.TO.SIGNIFICANT.GENES <- TRUE

#Significant genes from step 1 analysis:
SIGNIF.GENES <- "HepaRG-Ref-Step1-Jul17-signifgenes-2017-11-29.RData"

# Name of the ToxCast Curve fit file
HEPARG.table <-"LTEA_Level5&6_20150922.csv"

# Literature activities of the reference chemicals:
NR.CHEM.PRIOR.TABLE <- "NR-prior-ToxCast Assay Data-071315.xlsx"

# We repeat the observaions from the reference plates in order to weight them
# appropriately (more observations should mean more weight)
NUM.REFERENCE.PLATES <- 7

#Are we analyzing the reference chems or the ToxCast library
REFERENCE.CHEMS <- TRUE

# Literature NR-Gene intractions:
LITERATURE.NR.GENE.INTERACTIONS<- "LitConnectionsForPriors_v3.xlsm"
```

```
# END CONFIGURATION VARIABLES ################################################
##################################################################################

if (file.exists(paste(FILENAME,"-PREJAGS-",Sys.Date(),".RData",sep="")))
{
  load(paste(FILENAME,"-PREJAGS-",Sys.Date(),".RData",sep=""))
} else {
  NR.genes <- NULL

  # Now read in the chemical-specific NR interaction prior information (Jill/John):
  source("get-NRs-and-RefChems-070715.R")

  # Create the hepdata table with the simplified LTEA data:
  source("load-hepdata-072417.R")

  repdata <- subset(hepdata,casn%in%c("474-25-9","57-30-7","73590-58-6","42017-89-0"))
  for (i in 1:(NUM.REFERENCE.PLATES-1)) hepdata <- rbind(hepdata,repdata)

  # We use these tables as arguments to the function that builds the JAGS model:
  source("create-NR-prior-tables-033116.R")

  # Read table of literature NR-gene interactions with direction (Nancy Baker):
  source("read-literature-prior-072017.R")

  # Create the BUGS file:
  create.ref.model(RJAGS.MODEL.FILE,Num.NR)


  # We need a function tuo turn NR states into binary numbers:
  to.binary <- function(x,bits=5)
  {
    tmp <- paste(sapply(strsplit(paste(rev(intToBits(x))),""),`[[`,2),collapse="")
    return(substr(tmp,nchar(tmp)-bits+1,nchar(tmp)))
  }

  # And we use this to build a table of which NR's are off (0) or on (1) for each state:
  NR.cat.boolean <- NULL
  for(i in 0:(2^Num.NR-1)) NR.cat.boolean <-
cbind(NR.cat.boolean,as.numeric(strsplit(to.binary(i,Num.NR),"")[[1]]))

  # Create a small cluster with a core for each chain in the analysis:
  cl2 <- makeCluster(NUM.CHAINS)

  inits <- list()
  for (chain in 1:NUM.CHAINS)
  {
    NR.Gene.Network <- matrix(NA,nrow=Num.Genes,ncol=Num.NR)
    rownames(NR.Gene.Network) <- NR.genes
```

```
  colnames(NR.Gene.Network) <- NRs
  for (this.gene in NR.genes)
  {
   for (this.NR in NRs)
   {
      if (!is.na(step1.NR.weight.med[this.NR,this.gene])) NR.Gene.Network[this.gene,this.NR] <- runif(1,
step1.NR.weight.low[this.NR,this.gene], step1.NR.weight.high[this.NR,this.gene])
      else NR.Gene.Network[this.gene,this.NR] <- 0
   }
   NR.AC50.index <- NR.Chem.Prior.Mean
   NR.AC50.index[] <- NA
  }
  for (this.NR in NRs)
  {
   for (this.chem in Chem.Names)
   {
    if (length(which(NR.Chem.Prior[this.chem,,this.NR]==1))==1)
    {
     NR.AC50.index[this.chem,this.NR] <- which(NR.Chem.Prior[this.chem,,this.NR]==1)
    } else {
     NR.AC50.index[this.chem,this.NR] <- sample(which(NR.Chem.Prior[this.chem,,this.NR]==1),1)
    }
   }
  }
  inits[[paste("inits",chain,sep="")]] <- list(
   .RNG.seed=as.numeric(paste(rep(chain,6),sep="",collapse="")),
   .RNG.name="base::Super-Duper",
   Lit.Obs=rep(0.2,Num.Lit.Obs),
   log.Lit.Obs.Precision=runif(1,-6,-3),
   NR.Gene.Network=NR.Gene.Network,
   NR.AC50.index=matrix(NR.AC50.index,nrow=Num.Chems,ncol=Num.NR),
   P2min=runif(Num.Genes,0.1,0.2),
   P3min=runif(Num.Genes,0.1,0.2),
   Lit.Threshold=0.1)
 }

 jags.data <- list('Obs.State' = hepdata[,"state"],
                   'Num.Obs' = Num.Obs,
                   'Num.Genes' = Num.Genes,
                   'Num.Concs' = Num.Concs,
                   'Num.Chems' = Num.Chems,
                   'Num.NR' = Num.NR,
                   'NR.Chem.Prior' = NR.Chem.Prior,
                   'NR.cat.boolean' = NR.cat.boolean,
                   'Concs.Tested' = Concs.Tested,
                   'Chem.Possible.AC50s' = Chem.Possible.AC50s,
                   'Obs.Chem' = hepdata[,"chem.id"],
                   'Obs.Conc' = hepdata[,"conc.id"],
```

```
                'Obs.Gene' = hepdata[,"gene.id"],
                'Num.Lit.Obs' = Num.Lit.Obs,
                'Lit.Obs'= rep(NA,Num.Lit.Obs),
                'is.Signif.Lit.Obs' = rep(1,Num.Lit.Obs),
                'Lit.Obs.Gene'= NR.gene.prior.final[,"gene.id"],
                'Lit.Obs.NR'= NR.gene.prior.final[,"NR.id"]
                )

  save(inits,jags.data,RJAGS.MODEL.FILE,NUM.CHAINS,NR.genes,Chem.Names,file=paste(FILENAME,"-
PREJAGS-",Sys.Date(),".RData",sep=""))
}

cat(paste("\n\nBeginning a Bayesian run with ",Num.Obs," observations of gene expression for
",Num.Chems," chemicals.\nInferring an interaction matrix of ",Num.Genes," genes driven by ",Num.NR,"
nuclear receptors.\nPrior information on NR-gene interactions taken from ",Num.Lit.Obs," journal
articles.\n\n\n",sep=""))

coda.out <- autorun.jags(RJAGS.MODEL.FILE,
              n.chains = NUM.CHAINS,
              method="parallel", method.options=list(cl=cl2),
              summarise=T,
              inits = inits,
              max.time="2d",
              startsample=4000,
              adapt=5000,
              startburnin=10000,
              thin.sample=T,
              psrf.target = 1.1,
              data = jags.data,
              monitor = c('NR.Gene.Network'))


save(coda.out,NR.genes,Chem.Names,Ref.chems,Num.NR,NRs,SIGNIF.GENES,file=paste(FILENAME,"-
",Sys.Date(),".RData",sep=""))

   coda.out<-extend.jags(coda.out,
              sample=4000,
              adapt=5000,
              thin=10,
              add.monitor = c('NR.AC50',
                         'NR.AC50.index',
                         'P2min',
                         'P3min',
                         'Lit.Obs.Precision',
                         'Lit.Threshold'))

   save(coda.out,NR.genes,Chem.Names,Ref.chems,Num.NR,NRs,SIGNIF.GENES,file=paste(FILENAME,"-
",Sys.Date(),"-2.RData",sep=""))
```

```
  stopCluster(cl2)

# Get rid of anything in the workspace:
rm(list=ls())
```

heparg-tox-analysis-042318.r

```
library(runjags)
library(gdata)
library(parallel)
library(gplots)
library(devtools)
library(ggplot2)
library(scales)

# Read the results of the reference chem analysis:
load("HepaRG-Ref-Step2-Nov2017-2017-12-10-2.RData")

# Get rid of anything but the MCMC output and the reference chemicals:
rm(list=ls()[!(ls()%in%c("Ref.chems","coda.out","Num.NR","NRs","NR.genes"))])
# Kludge because I forgot to to save some of these:
Ref.chems  <-  c("474-25-9","57-30-7",  "13292-46-1",  "789-02-6",  "50892-23-4",    "58-18-4","50-29-3",
"42017-89-0", "72-43-5", "73590-58-6")
NRs <- c("AHR","AR","CAR","FXR","PPARA","PXR")
Num.NR <- 6

##########################################################################################
# CONFIGURATION VARIABLES ###############################################################

source("create.model-042018.R")

JAGS.PATH <- "/usr/local/bin/jags"

#Random number generator seed:
SEED <- 111111

#Set the filename stub:
FILENAME <- "HepaRG-ToxCast-Apr2018All"

# The number of Markov Chains used:
NUM.CHAINS <- 5

# How many subsets do we divide the data into:
DIVIDE.DATA <- 1

#Gens found to big significant in step 1 analysis:
SIGNIF.GENES<-"HepaRG-Ref-Step1-Jul17-signifgenes-2017-11-29.RData"

# Graph with only the significant (above threshold) genes:
GRAPH.FROM.INITIAL.ANALYSIS        <-        "HepaRG-Ref-Step2-Nov2017-NRgraph-Threshold1-Dropped-
Posterior-Graph-2017-12-11.RData"
RESTRICT.TO.SIGNIFICANT.GENES <- TRUE

RJAGS.MODEL.FILE <- paste(FILENAME,"-",Sys.Date(),".jags",sep="")
```

```
# Name of the ToxCast Curve fit file
HEPARG.table <-"LTEA_Level5&6_20150922.csv"

#Are we analyzing the reference chems or the ToxCast library
REFERENCE.CHEMS <- FALSE

# END CONFIGURATION VARIABLES ####################################################
####################################################################################


if (file.exists(paste(FILENAME,"-PREJAGS.RData",sep="")))
{
  load(paste(FILENAME,"-PREJAGS.RData",sep=""))
} else {
  # Read the output from the JAGS analysis of the reference chemicals to use a multivariate
  # normal prior:
  out.mcmc <- as.matrix(coda.out$mcmc[[1]])
  for (i in 2:length(coda.out$mcmc)) out.mcmc <- rbind(out.mcmc,as.matrix(coda.out$mcmc[[i]]))
  cn <- colnames(out.mcmc)
  # This is big so lets not keep it around:
  rm(coda.out)
  # Keep only the regulator model paramters:
  out.mcmc <- out.mcmc[,regexpr("AC50",cn)==-1&regexpr("Lit",cn)==-1]
  cn <- colnames(out.mcmc)
  P.include.vars <- cn[regexpr("min",cn)!=-1]
  P.multi.mu <- apply(out.mcmc[,P.include.vars],2,mean)
  P.multi.omega <- solve(cov(out.mcmc[,P.include.vars]))

  NR.include.vars <- cn[regexpr("min",cn)==-1]
  NR.Gene.Network.mu <- apply(out.mcmc[,NR.include.vars],2,mean)
  NR.include.vars <- names(NR.Gene.Network.mu[abs(NR.Gene.Network.mu)>0.5])
  NR.Gene.Network.mu <- apply(out.mcmc[,NR.include.vars],2,mean)
  temp <-out.mcmc[,NR.include.vars]-NR.Gene.Network.mu
  for (i in 1:length(NR.Gene.Network.mu)) temp[,i] <- temp[,i]/NR.Gene.Network.mu[i]
  NR.Gene.Network.omega <- solve(cov(temp))

  # Create the hepdata table with the simplified LTEA data:
  source("load-hepdata-121117.R")
  create.toxcast.model(RJAGS.MODEL.FILE,Num.NR)

  Chem.Possible.AC50s <- sort(unique(as.vector(Concs.Tested[!is.na(Concs.Tested)])))
  Chem.Possible.AC50s        <-        sort(c(Chem.Possible.AC50s,min(Chem.Possible.AC50s,na.rm=T)-
0.5,max(Chem.Possible.AC50s,na.rm=T)+0.5))
  NR.Chem.Prior <- matrix(0,nrow=Num.Chems,ncol=length(Chem.Possible.AC50s))

  NR.Chem.Prior[,1] <- 1
  NR.Chem.Prior[,dim(NR.Chem.Prior)[2]] <- 1
```

```
  rownames(NR.Chem.Prior) <- Chem.Names
  colnames(NR.Chem.Prior) <- Chem.Possible.AC50s


  # Only sample among the tested concentrations for each chemical:
  for (this.chem in Chem.Names)
    NR.Chem.Prior[this.chem,as.character(Concs.Tested[this.chem,!is.na(Concs.Tested[this.chem,])])] <- 1

  # We need a function tuo turn NR states into binary numbers:
  to.binary <- function(x,bits=5)
  {
    tmp <- paste(sapply(strsplit(paste(rev(intToBits(x))),""),`[[`,2),collapse="")
    return(substr(tmp,nchar(tmp)-bits+1,nchar(tmp)))
  }

  # And we use this to build a table of which NR's are off (0) or on (1) for each state:
  NR.cat.boolean <- NULL
  for(i in 0:(2^Num.NR-1)) NR.cat.boolean <-
cbind(NR.cat.boolean,as.numeric(strsplit(to.binary(i,Num.NR),"")[[1]]))

  save.image(file=paste(FILENAME,"-PREJAGS.RData",sep=""))
}

# Create a small cluster with a core for each chain in the analysis:
cl2 <- makeCluster(NUM.CHAINS)

set.seed(SEED)
which.subset <- sample(1:DIVIDE.DATA,Num.Chems,replace=T)
names(which.subset) <- Chem.Names
coda.out <- list()

#Any Nr weights that aren't being estimated are set to zero:
    non.zero.weights <-
names(NR.Gene.Network.mu)[regexpr("NR.Gene",names(NR.Gene.Network.mu))!=-1]
    non.zero.gene <- NULL
    non.zero.NR <- NULL
    NR.Gene.Network.data <- matrix(0,nrow=Num.Genes,ncol=Num.NR)
    for (i in 1:length(non.zero.weights))
    {
        temp.text <- strsplit(non.zero.weights[i],"\\[")[[1]][2]
        temp.text <- strsplit(temp.text,",")
        this.gene <- as.numeric(temp.text[[1]][1])
        this.NR <- as.numeric(strsplit(temp.text[[1]][2],"\\]")[[1]][1])
        non.zero.gene[i] <- this.gene
        non.zero.NR[i] <- this.NR
        NR.Gene.Network.data[this.gene,this.NR] <- NA
    }
    rownames(NR.Gene.Network.data) <- NR.genes
```

```
  colnames(NR.Gene.Network.data) <- NRs

for (this.subset in 1:DIVIDE.DATA)
{
# Subset down to roughyl a tenth of the chemicals (subsetted at random):
 these.chems <- Chem.Names[which.subset==this.subset]
# Calculate the number of chems in this subset:
 this.num.chems <- length(these.chems)
# Only look at the hepdata for these chems:
 this.hepdata.subset <- subset(hepdata,casn %in% these.chems)
# Need to reassign the chem id's:
 this.hepdata.subset$chem.id <- sapply(this.hepdata.subset$casn,function(x) which(x==these.chems))
# Need to reflect the size of the data subset:
 this.num.obs <- dim(this.hepdata.subset)[1]

 indices <- sample(1:dim(out.mcmc)[1],NUM.CHAINS)
 inits <- list()
# We need to set an initial value for each AC50
# The sample has trouble finding the posterior given ~6000 AC50s, so we need to
# try to get close:
 NR.AC50.index0 <- matrix(NA,this.num.chems,Num.NR,dimnames=list(these.chems,NRs))
 for (this.chem in these.chems)
 {
  NR.AC50.index0[this.chem,1:Num.NR] <- max(which(NR.Chem.Prior[this.chem,]==1),na.rm=T)
 }
 for (chain in 1:NUM.CHAINS)
 {
  NR.Gene.Network <- matrix(NA,nrow=Num.Genes,ncol=Num.NR)
  P2min <- rep(NA,Num.Genes)
  names(P2min) <- NR.genes
  P3min <- P2min
  rownames(NR.Gene.Network) <- NR.genes
  colnames(NR.Gene.Network) <- NRs
  for (this.gene in NR.genes)
  {
   P2min[this.gene]<-out.mcmc[indices[chain],paste("P2min[",which(NR.genes==this.gene),"]",sep="")]
   P3min[this.gene]<-out.mcmc[indices[chain],paste("P3min[",which(NR.genes==this.gene),"]",sep="")]
   for (this.NR in NRs)
    if (is.na(NR.Gene.Network.data[this.gene,this.NR]))
    {
     NR.Gene.Network[this.gene,this.NR]                                    <-
out.mcmc[indices[chain],paste("NR.Gene.Network[",which(NR.genes==this.gene),",",which(NRs==this.N
R),"]",sep="")]
    }
  }
  NR.Network <- rep(NA,length(non.zero.gene)+2*Num.Genes)
  for (i in 1:Num.Genes)
  {
```

```
    NR.Network[i] <- P2min[i]
    NR.Network[Num.Genes+i] <- P3min[i]
   }
   for (i in 1:length(non.zero.gene))
   {
    NR.Network[2*Num.Genes+i] <- NR.Gene.Network[non.zero.gene[i],non.zero.NR[i]]
   }
   P.multi <- P.multi.mu
   NR.Network.0 <- rep(0,length(non.zero.gene))
   NR.AC50.index <- NR.AC50.index0
   for (this.NR in NRs)
   {
# For each NR, pick only the genes that are relevant
    these.genes <- NR.genes[abs(NR.Gene.Network[,this.NR])>0.5]
# Subset the data to those chemicals that showed activity in genes relevant to NR:
    this.hepdata <-subset(this.hepdata.subset,gene%in%these.genes&state!=1)
# For each chem with activity, chose on of the ten lowest conc's with activity:
    for (this.chem in unique(this.hepdata$casn))
    {
     possible <- subset(this.hepdata,casn==this.chem)$conc.id
     if (length(possible) >10) possible <- sort(possible)[1:10]
     NR.AC50.index[this.chem,this.NR]                                          <-
which(Chem.Possible.AC50s==Concs.Tested[this.chem,sample(possible,1)])
    }
   }
   inits[[paste("inits",chain,sep="")]] <- list(
    .RNG.seed=as.numeric(paste(rep(chain,6),sep="",collapse="")),
    .RNG.name="base::Super-Duper",
    NR.Network.0=NR.Network.0,
    P.multi=P.multi,
    NR.AC50.index=matrix(NR.AC50.index,nrow=this.num.chems,ncol=Num.NR)
    )
 }

 jags.data <- list('Obs.State' = this.hepdata.subset[,"state"],
                 'Num.Obs' = this.num.obs,
                 'Num.Genes' = Num.Genes,
                 'Num.Concs' = Num.Concs[these.chems],
                 'Num.Chems' = this.num.chems,
                 'NR.Chem.Prior' = NR.Chem.Prior[these.chems,],
                 'Chem.Possible.AC50s' = Chem.Possible.AC50s,
                 'Num.NR' = Num.NR,
                 'NR.cat.boolean' = NR.cat.boolean,
                 'Concs.Tested' = as.matrix(Concs.Tested[these.chems,],num.rows=this.num.chems),
                 'Obs.Chem' = this.hepdata.subset[,"chem.id"],
                 'Obs.Conc' = this.hepdata.subset[,"conc.id"],
                 'Obs.Gene' = this.hepdata.subset[,"gene.id"],
                 'P.multi.mu'=P.multi.mu,
```

```
                    'P.multi.omega'=P.multi.omega,
                    'NR.network.prior.mu' = NR.Gene.Network.mu,
                    'NR.network.prior.omega' = NR.Gene.Network.omega,
                    'NR.Gene.Network'= NR.Gene.Network.data,
                    'non.zero.gene' = non.zero.gene,
                    'non.zero.NR' = non.zero.NR,
                    'Num.non.zero.weights' = length(non.zero.gene)
                    )


save(inits,which.subset,these.chems,this.subset,jags.data,RJAGS.MODEL.FILE,NUM.CHAINS,NR.genes,Ch
em.Names,file=paste(FILENAME,"-PREJAGS-",this.subset,"-",Sys.Date(),".RData",sep=""))

  cat(paste("\n\nBeginning a Bayesian run with ",jags.data$Num.Obs," observations of gene expression
for ",jags.data$Num.Chems," chemicals.\nInferring an interaction matrix of ",jags.data$Num.Genes,"
genes driven by ",jags.data$Num.NR," nuclear receptors.\n\n\n",sep=""))

  coda.out[[this.subset]] <- autorun.jags(RJAGS.MODEL.FILE,
              n.chains = NUM.CHAINS,
              method="parallel",
              method.options=list(cl=cl2),
 #              summarise=T,
              inits = inits,
#               max.time="4h",
              startsample=4000,
              adapt=4000,
              startburnin=10000,
 #              thin.sample=T,
              psrf.target = 1.2,
              data = jags.data,
              jags = JAGS.PATH,
              monitor = c('NR.Network'))

save(coda.out,NR.genes,Chem.Names,Num.Chems,Num.Genes,Num.NR,NRs,FILENAME,file=paste(FILEN
AME,"-",Sys.Date(),"-1.RData",sep=""))

  coda.out [[this.subset]] <-extend.jags(coda.out[[this.subset]],
                          add.monitor = c('NR.AC50.index'))


save(coda.out,NR.genes,Chem.Names,Num.Chems,Num.Genes,Num.NR,NRs,FILENAME,file=paste(FILEN
AME,"-",Sys.Date(),"-2.RData",sep=""))
}

stopCluster(cl2)

# Get rid of anything in the workspace:
rm(list=ls())
```

load-hepdata-121117.R

```
library(data.table)
library(tcpl)
library(reshape2)

if (file.exists(paste(FILENAME,"-hepdata.RData",sep="")))
{
  load(paste(FILENAME,"-hepdata.RData",sep=""))
} else {

cat("Retrieving chemical concentrations from invitroDB...\n")
###_____READ from in vitro DB
tcplConf(drvr = "MySQL",
         user = "_dataminer",
         pass = "pass",
         host = "134.67.216.183",
         db = "invitrodb"
#          int = TRUE
         )

tcplLoadAsid()
aeids <- tcplLoadAeid(fld = "asid", val = 9)$aeid
dat <- tcplPrepOtpt(tcplLoadData(lvl=4L, fld = "aeid", val = aeids))
###_____

HepaRG.all <- data.table(dat)
###_____

# The reference plate chemicals don't have casn, pull that from the chem prior table"
HepaRG.all[HepaRG.all$spid=="Omeprazole","casn"] <- "73590-58-6"
HepaRG.all[HepaRG.all$spid=="Rifampicin","casn"] <- "13292-46-1"
HepaRG.all[HepaRG.all$spid=="Fenofibric Acid","casn"] <- "42017-89-0"
HepaRG.all[HepaRG.all$spid=="Aflatoxin B1","casn"] <- "1162-65-8"
HepaRG.all[HepaRG.all$spid=="Chenodeoxycholic","casn"] <- "474-25-9"
HepaRG.all[HepaRG.all$spid=="Chenodeoxycholic Acid","casn"] <- "474-25-9"
HepaRG.all[HepaRG.all$spid=="Phenobarbital","casn"] <- "57-30-7"

# The reference plate chemicals don't have chnm, pull that from the chem prior table"
HepaRG.all[HepaRG.all$spid=="Omeprazole","chnm"] <- "Omeprazole"
HepaRG.all[HepaRG.all$spid=="Rifampicin","chnm"] <- "Rifampicin"
HepaRG.all[HepaRG.all$spid=="Fenofibric Acid","chnm"] <- "Fenofibric Acid"
HepaRG.all[HepaRG.all$spid=="Aflatoxin B1","chnm"] <- "Aflatoxin B1"
HepaRG.all[HepaRG.all$spid=="Chenodeoxycholic","chnm"] <- "Chenodeoxycholic acid"
HepaRG.all[HepaRG.all$spid=="Chenodeoxycholic Acid","chnm"] <- "Chenodeoxycholic acid"
HepaRG.all[HepaRG.all$spid=="Phenobarbital","chnm"] <- "Phenobarbital sodium"

###_____READ THE Lvl5&6 File (these are the hits only)
```

```
#HepaRG <-
read.csv("L:/Lab/NCCT_ToxCast/HepaRG/tcpl_analysis/LTEA_tcpl_1.0_June/LTEA_Level5&6_20150602.csv",header=T,sep=",",stringsAsFactors=F,quote="\"")

cat(paste("Retrieving curve fits from",HEPARG.table,"...\n"))
HepaRG <-read.csv(HEPARG.table,header=T,sep=",",stringsAsFactors=F,quote="\"")

#Get rid of ToxCast phenobarb for now:
#HepaRG.all <- subset(HepaRG.all,spid!="TX006463")
#HepaRG <- subset(HepaRG,spid!="TX006463")
#HepaRG.all <- subset(HepaRG.all,spid!="TP0000981H07")
#HepaRG <- subset(HepaRG,spid!="TP0000981H07")




# Extract the gene names from the assay name:
HepaRG$gene <- lapply(strsplit(HepaRG$aenm,"_"),function(x) x[3])

# The reference plate chemicals don't have casn, pull that from the chem prior table"
HepaRG[HepaRG$spid=="Omeprazole","casn"] <- "73590-58-6"
HepaRG[HepaRG$spid=="Rifampicin","casn"] <- "13292-46-1"
HepaRG[HepaRG$spid=="Fenofibric Acid","casn"] <- "42017-89-0"
HepaRG[HepaRG$spid=="Aflatoxin B1","casn"] <- "1162-65-8"
HepaRG[HepaRG$spid=="Chenodeoxycholic","casn"] <- "474-25-9"
HepaRG[HepaRG$spid=="Chenodeoxycholic Acid","casn"] <- "474-25-9"
HepaRG[HepaRG$spid=="Phenobarbital","casn"] <- "57-30-7"

# The reference plate chemicals don't have chnm, pull that from the chem prior table"
HepaRG[HepaRG$spid=="Omeprazole","chnm"] <- "Omeprazole"
HepaRG[HepaRG$spid=="Rifampicin","chnm"] <- "Rifampicin"
HepaRG[HepaRG$spid=="Fenofibric Acid","chnm"] <- "Fenofibric Acid"
HepaRG[HepaRG$spid=="Aflatoxin B1","chnm"] <- "Aflatoxin B1"
HepaRG[HepaRG$spid=="Chenodeoxycholic","chnm"] <- "Chenodeoxycholic acid"
HepaRG[HepaRG$spid=="Chenodeoxycholic Acid","chnm"] <- "Chenodeoxycholic acid"
HepaRG[HepaRG$spid=="Phenobarbital","chnm"] <- "Phenobarbital sodium"


if (any(is.na(HepaRG$casn))) stop("Problem with casn in HepaRG data")

# Similarly, the reference plate chemicals don't have a chnm:
HepaRG[is.na(HepaRG$chnm),"chnm"] <- HepaRG[is.na(HepaRG$chnm),"spid"]

# This is a big file, so lets get rid of any genes that don't have ref chem activity:
# We want to get rid of any genes that don't show activity for the reference chemicals:
NR.genes <- sort(unlist(unique(subset(HepaRG,casn%in%Ref.chems&hitc==1)$gene)))
NR.genes <- NR.genes[NR.genes!="LDH"] #For another day -- add "cytotox receptor" with strong prior on CR-LDH connection
```

```
if (RESTRICT.TO.SIGNIFICANT.GENES)
{
  load(SIGNIF.GENES)
  NR.genes <- NR.genes[NR.genes%in%signif.genes]
}
Num.Genes <- length(NR.genes)

HepaRG <- subset(HepaRG, gene %in% NR.genes)

# Now let's subest the data down to only the reference chemicals or the ToxCast library:
if (REFERENCE.CHEMS)
{
  hepdata <- subset(HepaRG,casn %in% Ref.chems)
} else {
  hepdata <- subset(HepaRG,!(casn %in% Ref.chems))
}

# Further subsetting to make sure that we have only the hits:
hepdata <-subset(hepdata,hitc==1)
hepdata<- subset(hepdata,regexpr("Hit-call potentially confounded by overfitting",hepdata$flag)==-1|is.na(hepdata$flag))
hepdata<- subset(hepdata,regexpr("Borderline active",hepdata$flag)==-1|is.na(hepdata$flag))
hepdata<- subset(hepdata,regexpr("Only one conc above baseline, active",hepdata$flag)==-1|is.na(hepdata$flag))

# Now pull only the columns that we will use:
hepdata <- hepdata[,c('spid','casn','hill_ga','aeid','aenm','gene')]
# A state of "3" means down-regulation:
hepdata$state <- 3
# Set the upregulation assays to state "2":
hepdata[regexpr("_up",hepdata$aenm)!=-1,"state"] <- 2

# Now we store the remaining chemicals in a vector:
Chem.Names <- unique(hepdata[,"casn"])
# And count the number of chemicals:
Num.Chems <- length(Chem.Names)
# It's useful to have the names instead of the CAS numbers as well:
Chem.Full.Names <- Chem.Names
for (i in 1:length(Chem.Names))
  Chem.Full.Names[i] <- HepaRG[HepaRG$casn==Chem.Names[i],"chnm"][1]

# Make a list of the concentrations tested for each chemical (eventually want
# a matrix but we don't know how big of a matrix yet):
Concs.Tested <- NULL
# The table hep.data.conc stores a row for each observation.
# We initialize the table to have an observation of 1 (unchanged) for every gene
# and every concentration:
cat("Building hep.data.conc.list...\n")
```

```
hep.data.conc.list <- NULL
row.count <- 0
for (this.cas in Chem.Names)
{
 if (!REFERENCE.CHEMS) if (!(which(Chem.Names==this.cas)%%round(length(Chem.Names)/100)))
cat(paste(round(which(Chem.Names==this.cas)/length(Chem.Names)*100,1),"%\n",sep=""))
 for (this.gene in NR.genes)
 {
  # HepaRG.all contains a full list of the LTEA data pulled in from the
  # invitroDB. It provides concentration information, and may contain assays that
  # are "hits" in the DB but we have thrown out. We want to create a set of
  # observations for every time the assay was run.
  # In invitro db the up and down modes ar etreated as two different asays,
  # but for our observations we lump them together.
  assays <-
c(paste("LTEA_HepaRG_",this.gene,"_dn",sep=""),paste("LTEA_HepaRG_",this.gene,"_up",sep=""))
  this.all.samples.data <- as.data.frame(subset(HepaRG.all,casn==this.cas &
aenm%in%assays))[,c("spid","aenm","nconc","logc_min","logc_max","hill_ga")]

  # If the assay wasn't run, don't generate data for it:
  if (dim(this.all.samples.data)[1]>0)
  {
   if (dim(this.all.samples.data)[1]>1)
   {
    keep.these.rows <- rep(T,dim(this.all.samples.data)[1])
    for (this.row in seq(1,length(keep.these.rows),2))
    {
    # If there is no activity we keep an arbitrary assay (the first of the two):
     if (all(is.na(this.all.samples.data[this.row:(this.row+1),"hill_ga"]))) keep.these.rows[this.row]<- F
     else
     {
      if (is.na(this.all.samples.data[this.row,"hill_ga"])) keep.these.rows[this.row] <- F
      else if (is.na(this.all.samples.data[this.row+1,"hill_ga"])) keep.these.rows[this.row+1] <- F
      else {
     # We don't do non-monotonic responses here (assuming cytotoxicity is the cause),
     # so just take the lower of the two activities (if there is more than one):
       if (this.all.samples.data[this.row,"hill_ga"] ==
min(this.all.samples.data[this.row:(this.row+1),"hill_ga"],na.rm=T)) keep.these.rows[this.row+1] <- F
       else keep.these.rows[this.row] <- F
      }
     }
    }
    # Keep just the rows we want:
    this.all.samples.data <- this.all.samples.data[keep.these.rows,]
   }
   # Hepdata contains the scrubbed list of hits from Jill, with certain hits
   # removed because of flags. I read this in from a CSV file.
   # This will be empty if there is no hit for this chemical-gene pair:
```

```
   this.hit.data <- subset(hepdata,casn==this.cas & gene==this.gene)

   # Loop over repeated assays:
   for (this.row in 1:dim(this.all.samples.data)[1])
   {
    num.conc <- this.all.samples.data[this.row,"nconc"]
    min.conc <- this.all.samples.data[this.row,"logc_min"]
    max.conc <- this.all.samples.data[this.row,"logc_max"]
    ac50 <- this.all.samples.data[this.row,"hill_ga"]

    row.count <- row.count+num.conc
    these.rows <- data.frame(
            casn=rep(this.cas,num.conc),
            gene=this.gene,
            state=1,
            conc=seq(min.conc,max.conc,(max.conc-min.conc)/(num.conc-1)),
            stringsAsFactors=F)

 # If the data isn't in hit data presume a no hit (all 1's)
    if (ac50 %in% this.hit.data[,"hill_ga"])
    {
     this.state <- this.hit.data[this.hit.data[,"hill_ga"]==ac50,"state"]
     these.rows[these.rows$conc > ac50,"state"] <- this.state
    }
    these.rows$conc <- round(these.rows$conc,1)
    hep.data.conc.list[[length(hep.data.conc.list)+1]] <- these.rows
    Concs.Tested[[this.cas]] <- sort(unique(c(Concs.Tested[[this.cas]],these.rows$conc)))
   }
  }
 }
}

# Further subsetting to make sure we have concentration data:
hepdata <-subset(hepdata,casn%in%names(Concs.Tested))
# Now we store the remaining chemicals in a vector:
Chem.Names <- unique(hepdata[,"casn"])
# And count the number of chemicals:
Num.Chems <- length(Chem.Names)
# It's useful to have the names instead of the CAS numbers as well:
Chem.Full.Names <- Chem.Names
for (i in 1:length(Chem.Names))
 Chem.Full.Names[i] <- HepaRG[HepaRG$casn==Chem.Names[i],"chnm"][1]

cat("Converting hep.data.conc.list into dataframe...\n")
hep.data.conc <- data.frame(
          casn=rep("temp",row.count),
          gene=rep("temp",row.count),
          state=rep(1,row.count),
```

```
        conc=rep(-99,row.count),stringsAsFactors=F)
this.row <- 1
this.index <- 1
while (this.index <= length(hep.data.conc.list))
{
  if (!REFERENCE.CHEMS) if (!(this.index%%round(length(hep.data.conc.list)/100))) cat(paste(round(this.index/length(hep.data.conc.list)*100,1),"%\n",sep=""))
  data.points <- dim(hep.data.conc.list[[this.index]])[1]
  hep.data.conc[this.row:(this.row+data.points-1),] <- hep.data.conc.list[[this.index]]
  this.row <- this.row+data.points
  this.index <- this.index+1
}

# We overwrite the temporary (hits only) hepdata table with the table containing
# data for each concentration:

hepdata <- hep.data.conc

source("plot.data-062915.R")
plot.data(hepdata,NR.genes,Chem.Names,Chem.Full.Names,file.stub=paste(FILENAME,"-Simplified-LTEA-Data-",sep=""))

# For each observation we need id's to tell JAGS what gene, chemical, and concentration the observation refers to:
hepdata$gene.id <- sapply(hepdata$gene,function(x) which(x==NR.genes))
hepdata$chem.id <- sapply(hepdata$casn,function(x) which(x==Chem.Names))

# Now we know how many observations in total we have:
Num.Obs <- dim(hepdata)[1]

# We want to be able to identify the concentration for each observation:
max.concs <- max(unlist(lapply(Concs.Tested,length)))
Concs.matrix <- matrix(NA,nrow=Num.Chems,ncol=max.concs)
Num.Concs <- rep(0,Num.Chems)
rownames(Concs.matrix) <- Chem.Names
names(Num.Concs) <- Chem.Names
for (this.cas in Chem.Names)
{
  Num.Concs[this.cas] <- length(Concs.Tested[[this.cas]])
  Concs.matrix[this.cas,1:Num.Concs[this.cas]]<-Concs.Tested[[this.cas]]
  hepdata[hepdata$casn==this.cas,"conc.id"] <- sapply(hepdata[hepdata$casn==this.cas,"conc"],function(x) which(x==Concs.Tested[[this.cas]]))
}

Concs.Tested <- Concs.matrix

rm("dat","Concs.matrix","hep.data.conc","hep.data.conc.list","HepaRG","HepaRG.all","HEPARG.table")
```

```
  #save(hepdata,max.conc,file=paste(FILENAME,"-simplifieddata-",Sys.Date(),".RData",sep=""))
  save.image(file=paste(FILENAME,"-hepdata.RData",sep=""))
}
```

analyze-step1-112917.R

```
rm(list=ls())

load("HepaRG-Ref-Step1-Jul17-2017-11-17.RData")
#1:
load("HepaRG-Ref-Step1-Jul17-AHR-2017-08-25.RData")
outlist[[1]] <- list(genelist=genelist,coda.out=coda.out)
#2:
load("HepaRG-Ref-Step1-Jul17-AR-2017-08-25.RData")
outlist[[2]] <- list(genelist=genelist,coda.out=coda.out)
#3:
#load("HepaRG-Ref-Step1-Jul17-CAR-2017-11-17.RData")
#4:
load("HepaRG-Ref-Step1-Jul17-FXR-2017-08-25.RData")
outlist[[4]] <- list(genelist=genelist,coda.out=coda.out)
#5:
load("HepaRG-Ref-Step1-Jul17-PPARA-2017-08-25.RData")
outlist[[5]] <- list(genelist=genelist,coda.out=coda.out)
#6:
load("HepaRG-Ref-Step1-Jul17-PXR-2017-08-26.RData")
outlist[[6]] <- list(genelist=genelist,coda.out=coda.out)




signif.genes <- NULL
step1.NR.weight.low <- NULL
for (i in 1:length(NRs))
{
  genelist <- outlist[[i]][[1]]
  step1.NR.weight.low <- rbind(step1.NR.weight.low,NA)
  M <-dim(step1.NR.weight.low)[1]
  rownames(step1.NR.weight.low)[M] <- NRs[i]
  new.genes <-genelist[!(genelist %in% colnames(step1.NR.weight.low))]
  L <- length(new.genes)
  if (L>0)
  {
    for (j in 1:length(new.genes))
    {
      step1.NR.weight.low <- cbind(step1.NR.weight.low,NA)
      colnames(step1.NR.weight.low)[dim(step1.NR.weight.low)[2]] <- new.genes[j]
    }
  }
}
step1.NR.weight.high <- step1.NR.weight.low
step1.NR.weight.med <- step1.NR.weight.low
```

```
for (i in 1:length(NRs))
{
  out.mcmc <- as.matrix(outlist[[i]][[2]]$mcmc[[1]])
  for (j in 2:length(outlist[[i]][[2]]$mcmc)) out.mcmc <- 
rbind(out.mcmc,as.matrix(outlist[[i]][[2]]$mcmc[[j]]))
# means <- apply(out.mcmc[,regexpr("NR.Gene.Net",colnames(out.mcmc))!=-1 
&regexpr(",2]",colnames(out.mcmc))==-1],2,mean)
  low <- apply(out.mcmc[,regexpr("NR.Gene.Net",colnames(out.mcmc))!=-1 
&regexpr(",2]",colnames(out.mcmc))==-1],2,function(x) quantile(x,probs=0.25))
  med <- apply(out.mcmc[,regexpr("NR.Gene.Net",colnames(out.mcmc))!=-1 
&regexpr(",2]",colnames(out.mcmc))==-1],2,function(x) quantile(x,probs=0.5))
  high <- apply(out.mcmc[,regexpr("NR.Gene.Net",colnames(out.mcmc))!=-1 
&regexpr(",2]",colnames(out.mcmc))==-1],2,function(x) quantile(x,probs=0.75))

  #gene.weights <- means[regexpr("NR.Gene.Net",names(means))!=-1 &regexpr(",2]",names(means))==-
1]
  genelist <- outlist[[i]][[1]]
  for (j in 1:length(genelist))
  {
    step1.NR.weight.low[NRs[i],genelist[j]] <- low[j]
    step1.NR.weight.med[NRs[i],genelist[j]] <- med[j]
    step1.NR.weight.high[NRs[i],genelist[j]] <- high[j]
  }
#  names(gene.weights) <- genelist

  #out.mcmc[,regexpr("sum",colnames(out.mcmc))!=-1][,1:10]
  #out.mcmc[,regexpr("PW",colnames(out.mcmc))!=-1][,1:10]
  #out.mcmc[,regexpr("P2\\[1,",colnames(out.mcmc))!=-1]
  #out.mcmc[,regexpr("P3\\[1,",colnames(out.mcmc))!=-1]
  #out.mcmc[,regexpr("NR.Gene",colnames(out.mcmc))!=-1&regexpr(",2]",colnames(out.mcmc))!=-1]
}


#signif.genes <- sort(colnames(NR.weight.low)[apply(NR.weight.med,2,function(x) 
any(abs(x)>0.2,na.rm=T))])
#signif.genes <- sort(colnames(step1.NR.weight.med))[apply(t(apply(step1.NR.weight.med,1,function(x) 
x/max(abs(x),na.rm=T)))>0.1,2,function(x) any(x,na.rm=T))]

# 95% confident non-zero:
signif.genes <- sort(colnames(step1.NR.weight.high)[apply(step1.NR.weight.high<0 
&step1.NR.weight.low<0,2,function(x) any(x,na.rm=T))])
signif.genes <- sort(unique(c(signif.genes,
  colnames(step1.NR.weight.high)[apply(step1.NR.weight.high>0   &step1.NR.weight.low>0,2,function(x) 
any(x,na.rm=T))])))
```

```
step1.NR.weight.low <- NR.weight.low[,signif.genes]
step1.NR.weight.med <- NR.weight.med[,signif.genes]
step1.NR.weight.high <- NR.weight.high[,signif.genes]


save(signif.genes,step1.NR.weight.low,step1.NR.weight.med,step1.NR.weight.high,file=paste(FILENAME
,"-signifgenes-",Sys.Date(),".RData",sep=""))
```

build-posterior-network-043018.r

```
library(igraph)

Network.Up <- matrix(0,nrow=Num.Genes,ncol=Num.NR)
rownames(Network.Up) <- NR.genes
colnames(Network.Up) <- NRs
Network.Down <- Network.Up
for (i in 1:length(jags.data$non.zero.gene))
{
  this.gene <- jags.data$non.zero.gene[i]
  this.nr <- jags.data$non.zero.NR[i]
  this.weight <- median(out.mcmc[,paste("NR.Network[",i,"]",sep="")])
  if (this.weight > 0)
  {
    Network.Up[this.gene,this.nr] <- this.weight
  } else {
    Network.Down[this.gene,this.nr] <- -this.weight
  }
}
```

create.model-042018.r

```
create.ref.model <- function(filename,Num.NR)
{
  cat("#------------------------------------------------------------------------------
# Name:      ",filename,"
# Description: A JAGS model for drawing inference of Nuclear Receptor (NR)
#            activation from gene expression data
#
# Author:     John Wambaugh, based on code by Robert Pearce
#
# Created:    ",date(),"
#------------------------------------------------------------------------------

model {

  for (i in 1:Num.Chems) {
    for (j in 1:Num.NR) {
      NR.AC50.index[i,j] ~ dcat(NR.Chem.Prior[i,,j])
      NR.AC50[i,j] <- Chem.Possible.AC50s[i,NR.AC50.index[i,j]]
    }
  }

  for (i in 1:Num.Chems) {
    for (j in 1:Num.Concs[i]) {
    NR.state[i,j] <- 1",file=filename,sep="")
  for (i in 1:Num.NR) cat(" + ",2^(Num.NR-i),"*step(Concs.Tested[i,j]-NR.AC50[i,",as.integer(i),"])",file=filename,append=T,sep="")
cat("\n    }
  }

  # From mclark:
  # Half-cauchy as in Gelman 2006
  # If scale parameter in cauchy is 5, precision of z = 1/5^2 = 0.04
  # sigma int
  #   sd_int <- zInt/sqrt(chSqInt) # prior for sigma; cauchy = normal/sqrt(chi^2)
  #   zInt ~ dnorm(0, .04)I(0,)
  #   chSqInt ~ dgamma(0.5, 0.5)   # chi^2 with 1 d.f.
  # Carvalho Horseshoe prior as coded by Allen Riddell:
  tau ~ dt(0, 1, 1)T(0,)

  for (i in 1:Num.Genes) {
    for (j in 1:Num.NR) {
      lambda[i,j] ~ dt(0, 1, 1)T(0,)
      NR.Gene.Network[i,j] ~ dnorm(0,lambda[i,j] * tau)T(-50,50)
    }
  }

  Lit.Threshold ~ dunif(0,50)
```

```
 log.Lit.Obs.Precision ~ dunif(-7,1)
 Lit.Obs.Precision <- 10^log.Lit.Obs.Precision
 for (i in 1:Num.Lit.Obs) {
  Lit.Obs[i] ~ dnorm(abs(NR.Gene.Network[Lit.Obs.Gene[i],Lit.Obs.NR[i]]),Lit.Obs.Precision)
  is.Signif.Lit.Obs[i] ~ dinterval(Lit.Obs[i],Lit.Threshold)
 }

 for (i in 1:Num.Genes)
 {
  P2min[i] ~ dunif(0.0001,0.5)
  P3min[i] ~ dunif(0.0001,0.5)
 }

 ",file=filename,append=T)
 if (Num.NR > 1) cat("Network.Response <- NR.Gene.Network %*%
NR.cat.boolean",file=filename,append=T)
 else cat("Network.Response[,2] <- NR.Gene.Network",file=filename,append=T)
cat("\n
 P2 <- (Network.Response+abs(Network.Response))/2
 P3 <- (abs(Network.Response)-Network.Response)/2


# We have three behaviors we want to ensure:
# 1) P2 and P3 are always non-zero (assay noise or stimulus by other receptors
#    not modeled)
# 2) P1 does not go to zero when many NR's are active (assay noise again)
# 3) The stiffness of P1 -- is it switch like (immediately drops for any NR
#    activation) or more gradual?

# We also want to weight the data for the replicate plates, without having to
# replicate the data. Need to do a weird fix to the dcat distribution to do this.
# Basically, the density d for n replicates is d^n. In order to achieve this with
# a categorical distibution, we need a fourth category that soaks up the remaining
# probability, otherwise our correctin will get messed up when dcat renormalizes
# everything.

 for (i in 1:Num.Obs)
 {
  PW[i,1] <- 1 - P2min[Obs.Gene[i]] - P3min[Obs.Gene[i]]
  PW[i,2] <- P2min[Obs.Gene[i]]+P2[Obs.Gene[i],NR.state[Obs.Chem[i],Obs.Conc[i]]]
  PW[i,3] <- P3min[Obs.Gene[i]]+P3[Obs.Gene[i],NR.state[Obs.Chem[i],Obs.Conc[i]]]
  Obs.State[i] ~ dcat(PW[i,])
 }
}",file=filename,append=T)
}

create.toxcast.model <- function(filename,Num.NR)
{
```

```
  cat("#------------------------------------------------------------------------------
# Name:       ",filename,"
# Description: A JAGS model for drawing inference of Nuclear Receptor (NR)
#             activation from gene expression data
#
# Author:      John Wambaugh, based on code by Robert Pearce
#
# Created:    ",date(),"
#------------------------------------------------------------------------------

model {
  for (i in 1:Num.Chems) {
    for (j in 1:Num.NR) {
      NR.AC50.index[i,j] ~ dcat(NR.Chem.Prior[i,])
    }
  }

  for (i in 1:Num.Chems) {
    for (j in 1:Num.Concs[i]) {
    NR.state[i,j] <- 1",file=filename,sep="")
  for (i in 1:Num.NR) cat(" + ",2^(Num.NR-i),"*step(Concs.Tested[i,j]-Chem.Possible.AC50s[NR.AC50.index[i,",as.integer(i),"]])",file=filename,append=T,sep="")
  cat("\n    }
  }

  P.multi ~ dmnorm(P.multi.mu,P.multi.omega)

  # Read in the P2 and P3 min from the multivariate normal:
  for (i in 1:Num.Genes)
  {
  # Multivariate normal distribution doesn't allow truncation:
    P2min[i] <- max(0.0001,min(0.5,P.multi[i]))
    P3min[i] <- max(0.0001,min(0.5,P.multi[Num.Genes+i]))
  }

  NR.Network.0 ~ dmnorm(rep(0,length(NR.network.prior.mu)),NR.network.prior.omega)
  NR.Network <- NR.network.prior.mu+NR.Network.0%*%NR.network.prior.mu

  # Read in the non-zero weights from the multivariate normal:
  for (i in 1:Num.non.zero.weights)
  {
    NR.Gene.Network[non.zero.gene[i],non.zero.NR[i]] <- NR.Network[i]
  }

  Network.Response <- NR.Gene.Network %*% NR.cat.boolean
  P2 <- (Network.Response+abs(Network.Response))/2
  P3 <- (abs(Network.Response)-Network.Response)/2
```

```
# We have three behaviors we want to ensure:
# 1) P2 and P3 are always non-zero (assay noise or stimulus by other receptors
#     not modeled)
# 2) P1 does not go to zero when many NR's are active (assay noise again)
# 3) The stiffness of P1 -- is it switch like (immediately drops for any NR
#     activation) or more gradual?

# We also want to weight the data for the replicate plates, without having to
# replicate the data. Need to do a weird fix to the dcat distribution to do this.
# Basically, the density d for n replicates is d^n. In order to achieve this with
# a categorical distibution, we need a fourth category that soaks up the remaining
# probability, otherwise our correctin will get messed up when dcat renormalizes
# everything.

  for (i in 1:Num.Genes)
  {
    for (j in 1:",2^Num.NR,")
    {
      PW[i,j,1] <- 1 - P2min[i] - P3min[i]
      PW[i,j,2] <- P2min[i]+P2[i,j]
      PW[i,j,3] <- P3min[i]+P3[i,j]
    }
  }

  for (i in 1:Num.Obs)
  {
    Obs.State[i] ~ dcat(PW[Obs.Gene[i],NR.state[Obs.Chem[i],Obs.Conc[i]],])
  }

}\n",file=filename,append=T)
}
```

create-NR-prior-tables-033116.R

```
# We use this table as an argument to the function that builds the JAGS model:
NR.Chem.Prior.Mean <- array(NA,dim=c(Num.Chems,Num.NR))
rownames(NR.Chem.Prior.Mean) <-  Chem.Names
colnames(NR.Chem.Prior.Mean) <- NRs
NR.Chem.Prior.SD <- NR.Chem.Prior.Mean
NR.Chem.Prior.Prec <- NR.Chem.Prior.Mean
for (this.row in 1:dim(NR.chem.prior)[1])
{
  this.cas <- NR.chem.prior[this.row,"CAS"]
  this.ac50 <- NR.chem.prior[this.row,"AC50"]
  this.nr <- toupper(NR.chem.prior[this.row,"NR"])
  if (!(this.nr %in% toupper(NRs))) stop(paste(this.nr,"from Reference Chemicals worksheet not found on
Genes worksheet"))
  if (!(this.cas %in% rownames(NR.Chem.Prior.Mean))) warning(paste(this.cas,"from Reference Chemicals
worksheet not found in genedata.subset"))
  else {
    NR.Chem.Prior.Mean[this.cas,this.nr] <- this.ac50
    NR.Chem.Prior.SD[this.cas,this.nr] <- 20
    NR.Chem.Prior.Prec[this.cas,this.nr] <- NR.Chem.Prior.SD[this.cas,this.nr]^-2
  }
}

Chem.Possible.AC50s <- cbind(Concs.Tested,apply(Concs.Tested,1,function(x) min(x,na.rm=T))-
0.5,apply(Concs.Tested,1,function(x) max(x,na.rm=T))+0.5)
Chem.Possible.AC50s <- t(apply(Chem.Possible.AC50s,1,function(x)  sort(x,na.last=T)))
NR.Chem.Prior <-
array(rep(Chem.Possible.AC50s,Num.NR),dim=c(Num.Chems,dim(Chem.Possible.AC50s)[2],Num.NR),di
mnames=list(Chem.Names,NULL,NRs))

NR.Chem.Prior[] <- 0
for (this.chem in Chem.Names)
  for (this.NR in NRs)
  {
    if (!is.na(NR.Chem.Prior.Mean[this.chem,this.NR]))
    {
      mnc <- min(Concs.Tested[this.chem,],na.rm=T)
      mxc <- max(Concs.Tested[this.chem,],na.rm=T)
      if (NR.Chem.Prior.Mean[this.chem,this.NR]<200)
      {
        # Prior information that there is activity:
        if (log10(NR.Chem.Prior.Mean[this.chem,this.NR]) < mnc)
        {
          # Very potent activity (e.g.,testosterone):
          NR.Chem.Prior[this.chem,Chem.Possible.AC50s[this.chem,]<=(mnc+0.5),this.NR] <- 1
        } else NR.Chem.Prior[this.chem,Chem.Possible.AC50s[this.chem,]<=mxc,this.NR] <- 1
      } else if (this.chem == "57-30-7" & NR.Chem.Prior.Mean[this.chem,this.NR]<600) {
        #Phenobarb is a special case:
```

```
      NR.Chem.Prior[this.chem,Chem.Possible.AC50s[this.chem,]<=mxc,this.NR] <- 1
     } else {
      # Prior information that there is no potent activity:
      NR.Chem.Prior[this.chem,Chem.Possible.AC50s[this.chem,]>mxc,this.NR] <- 1
     }
    } else NR.Chem.Prior[this.chem,,this.NR] <- 1
# Empty indices should not be sampled:
    NR.Chem.Prior[this.chem,is.na(Chem.Possible.AC50s[this.chem,]),this.NR] <- 0
  }
Chem.Possible.AC50s[is.na(Chem.Possible.AC50s)]<-99
source("plot-NR-AC50-prior-112415.R")
```

create-refchem-figure-052218.R

```
library(ComplexHeatmap)
library(circlize)

setwd("L:/Lab/NCCT_ExpoCast/ExpoCast2018/heparg")
load("L:/Lab/NCCT_ExpoCast/ExpoCast2018/heparg/HepaRG-Ref-Step1-Jul17-2017-11-02-
preJAGS.RData")

#source("plot-NR-AC50-prior-112415.R")
#source("plot.data-062915.R")
#plot.data(hepdata,NR.genes,Chem.Names,Chem.Full.Names,file.stub=paste(FILENAME,"-Simplified-
LTEA-Data-",sep=""))

Num.Genes <- length(NR.genes)
Num.Chems <- length(Chem.Names)

max.conc <- max(hepdata$conc)

hep.heatmap <- matrix(0,ncol=Num.Genes,nrow=Num.Chems)
colnames(hep.heatmap) <- NR.genes
rownames(hep.heatmap) <- Chem.Full.Names
for (this.chem in Chem.Full.Names)
{
  print(this.chem)
  for (this.gene in NR.genes)
  {
    max.conc <- max(Concs.Tested[Chem.Full.Names==this.chem],na.rm=T)
    this.data <- subset(hepdata,gene==this.gene&casn==Chem.Names[Chem.Full.Names==this.chem])
    if (any(this.data$state==2))
    {
      ac50 <- subset(this.data,state==2)
      ac50 <- min(ac50$conc)
      hep.heatmap[this.chem,this.gene] <- max.conc-ac50
    } else if (any(this.data$state==3)) {
      ac50 <- subset(this.data,state==3)
      ac50 <- min(ac50$conc)
      hep.heatmap[this.chem,this.gene] <- ac50-max.conc
    }
  }
}
# Remove genes that showed no activity:
hep.heatmap <- hep.heatmap[,apply(hep.heatmap,2,function(x) !all(is.na(x)))]
hep.heatmap <- hep.heatmap[,apply(hep.heatmap,2,function(x) !all(x==0))]
```

```
NR.AC50.prior <- matrix(NA,nrow=Num.Chems,ncol=Num.NR)
rownames(NR.AC50.prior) <- Chem.Full.Names
colnames(NR.AC50.prior) <- NRs
for (this.chem in Chem.Full.Names)
{
  print(this.chem)
  max.conc <- max(Concs.Tested[Chem.Full.Names==this.chem],na.rm=T)
  print(max.conc)
  for (this.NR in NRs)
  {
    if (!is.na(NR.Chem.Prior.Mean[Chem.Names[Chem.Full.Names==this.chem],this.NR]))
    {
      if (log10(NR.Chem.Prior.Mean[Chem.Names[Chem.Full.Names==this.chem],this.NR]) < max.conc)
      {
        NR.AC50.prior[this.chem,this.NR] <- max.conc-
log10(NR.Chem.Prior.Mean[Chem.Names[Chem.Full.Names==this.chem],this.NR])
      } else {
        NR.AC50.prior[this.chem,this.NR] <- 0
      }
    }
  }
}

ht1 = Heatmap(hep.heatmap,
        name = "ht1",
        col = colorRamp2(c(-3.5, 0, 3.5), c("red", "white", "green")),
        row_title = "Reference Chemicals",
        column_title="Genes",
        column_names_gp = gpar(fontsize = 10),
        heatmap_legend_param = list(title = "Potency\n(Up/Down)", at = c(-3,-1, 0,1,3),labels =c("0.1 uM
(down)","10 uM (down)","> 100 uM","10 uM (up)","0.1 uM (up)"),
        color_bar = "discrete"))

ht2 = Heatmap(NR.AC50.prior, name = "ht2",
    show_row_names=F,
    col = colorRamp2(c(-3.5, 0, 3.5), c("red", "white", "green")),
    row_title = "Reference Chemicals",
    column_names_gp = gpar(fontsize = 10),
    column_title="Reference NR Potency",
    show_heatmap_legend =F)

ht2+ht1

write.csv(hep.heatmap,file=paste("RefChem-GeneActivity-",Sys.Date(),".txt",sep=""))
write.csv(NR.AC50.prior,file=paste("RefChem-NRPrior-",Sys.Date(),".txt",sep=""))
```

do.JAGS-090617.R

```
# Now we run JAGS:
do.JAGS <- function(this.NR)
{
 if(length(list.files(pattern=paste(FILENAME,"-",this.NR,"-2017*",sep="")))==0)
 {

 these.ref.chems <- subset(NR.chem.prior,AC50<100 & AC50!=1 & tolower(NR)==tolower(this.NR))$CAS
 if (this.NR == "CAR") these.ref.chems <- c(these.ref.chems,"57-30-7")
 these.ref.chems <- unique(these.ref.chems)
 temp.hepdata <- subset(hepdata,casn %in% these.ref.chems)
 temp.hepdata <- subset(temp.hepdata, conc<1.5 | casn=="57-30-7")
 temp.hepdata$gene <- as.character(temp.hepdata$gene)

 genelist <- NULL
 for (this.gene in unique(hepdata$gene))
 {
  if (any(subset(temp.hepdata,gene==this.gene)$state>1)) genelist <- c(genelist,this.gene)
 }
 cat(paste(this.NR,": ",genelist,"\n"))
 Num.Genes <- length(genelist)
 this.hepdata <- subset(hepdata,gene %in% genelist)
 this.hepdata$gene <- as.character(this.hepdata$gene)
 this.hepdata$gene.id <- sapply(this.hepdata$gene,function(x) which(genelist==x))

 Num.Obs <- dim(this.hepdata)[1]
 Num.NR <- 1

 # And we use this to build a table of which NR's are off (0) or on (1) for each state:
 NR.cat.boolean <- NULL

 for(i in 0:(2^Num.NR-1)) NR.cat.boolean <-
cbind(NR.cat.boolean,as.numeric(strsplit(to.binary(i,Num.NR),"")[[1]]))


 this.NR.gene.prior <- subset(NR.gene.prior.final,NR==this.NR & genesymbol%in%genelist)
 this.NR.gene.prior$gene.id <- sapply(this.NR.gene.prior$genesymbol,function(x) which(genelist==x))
 this.NR.gene.prior$NR.id <- 1
 Num.Lit.Obs <- dim(this.NR.gene.prior)[1]

#
save(RJAGS.MODEL.FILE,NUM.CHAINS,Num.NR,Num.Genes,NR.Chem.Prior.Mean,NR.Chem.Prior,this.N
R,Num.Lit.Obs.Up,Num.Lit.Obs.Down,genelist,these.ref.chems,Chem.Names,this.hepdata,file=paste(FIL
ENAME,"-",this.NR,"-PREJAGS-",Sys.Date(),".RData",sep=""))

 # We need intial conditions for the censored (literature) data:
 inits <- list()
 for (chain in 1:NUM.CHAINS)
```

```
 {
   NR.Gene.Network                              <-                              matrix(sample(c(rep(0,9),1,-
1),Num.Genes,replace=T),nrow=Num.Genes,ncol=Num.NR)
   NR.AC50.index <- NR.Chem.Prior.Mean[,1]
   NR.AC50.index[] <- NA
   for (i in Chem.Names)
     if (length(which(NR.Chem.Prior[i,,this.NR]==1))==1)
     {
       NR.AC50.index[i] <- which(NR.Chem.Prior[i,,this.NR]==1)
     } else {
       NR.AC50.index[i] <- sample(which(NR.Chem.Prior[i,,this.NR]==1),1)
     }
 #   if  (any(NR.AC50  >  NR.Chem.Prior.Max[,this.NR])  |  any(NR.AC50  <  NR.Chem.Prior.Min[,this.NR]))
browser()
   inits[[paste("inits",chain,sep="")]] <- list(
     .RNG.seed=as.numeric(paste(rep(chain,6),sep="",collapse="")),
     .RNG.name="base::Super-Duper",
     Lit.Obs=rep(0.2,Num.Lit.Obs),
     log.Lit.Obs.Precision=runif(1,-6,-3),
     NR.Gene.Network=NR.Gene.Network,
     NR.AC50.index=matrix(NR.AC50.index,nrow=Num.Chems,ncol=1),
   #  P.lowest=10^runif(1,-2,-1),
     P2min=runif(Num.Genes,0.1,0.2),
     P3min=runif(Num.Genes,0.1,0.2),
     Lit.Threshold=0.1)
 }

  this.data <- list('Obs.State' = this.hepdata[,"state"],
                    'Num.Obs' = Num.Obs,
                    'Num.Genes' = Num.Genes,
                    'Num.Concs' = Num.Concs,
                    'Num.Chems' = Num.Chems,
                    'Num.NR' = Num.NR,
                    "Network.Response" = as.matrix(cbind(rep(0,Num.Genes),rep(NA,Num.Genes))),
                    'NR.Chem.Prior'                                                              =
array(NR.Chem.Prior[Chem.Names,,this.NR],dim=c(dim(NR.Chem.Prior)[1:2],1)),
#                     'NR.cat.boolean' = NR.cat.boolean,
                    'Concs.Tested' = Concs.Tested,
                    'Chem.Possible.AC50s' = Chem.Possible.AC50s,
                    'Obs.Chem' = this.hepdata[,"chem.id"],
                    'Obs.Conc' = this.hepdata[,"conc.id"],
                    'Obs.Gene' = this.hepdata[,"gene.id"],
                    'Num.Lit.Obs' = Num.Lit.Obs,
                    'Lit.Obs'= rep(NA,Num.Lit.Obs),
                    'is.Signif.Lit.Obs' = rep(1,Num.Lit.Obs),
                    'Lit.Obs.Gene'= this.NR.gene.prior[,"gene.id"],
                    'Lit.Obs.NR'= rep(1,Num.Lit.Obs))
```

```
save(inits,this.data,RJAGS.MODEL.FILE,NUM.CHAINS,this.NR,genelist,these.ref.chems,Chem.Names,file=
paste(FILENAME,"-",this.NR,"-PREJAGS-",Sys.Date(),".RData",sep=""))

  cat(paste("\n\nBeginning a Bayesian run with ",Num.Obs," observations of gene expression for
",Num.Chems," chemicals.\nInferring an interaction matrix of ",Num.Genes," genes driven by ",Num.NR,"
nuclear receptors.\nPrior information on NR-gene interactions taken from ",Num.Lit.Obs," journal
articles.\n\n\n",sep=""))

  coda.out <- autorun.jags(RJAGS.MODEL.FILE,
            n.chains = NUM.CHAINS,
            method="parallel", method.options=list(cl=cl2),
            summarise=T,
            inits = inits,
            startsample=4000,
            thin.sample=T,
            adapt=200000,
            startburnin=100000,
            psrf.target = 1.20,
            data = this.data,
            max.time="2w",
            monitor = c('NR.Gene.Network'))
  save(coda.out,genelist,Chem.Names,file=paste(FILENAME,"-",this.NR,"-",Sys.Date(),".RData",sep=""))

  return(list(genelist=genelist,coda.out=coda.out))
  }
}
```

get-NRs-and-RefChems-070715.R

```
# Now read in the chemical-specific NR interaction prior information (Jill/John):
NR.chem.prior <- read.xls(NR.CHEM.PRIOR.TABLE,stringsAsFactors=F)
# Make sure the AC50's are numeric (not text)
NR.chem.prior$AC50 <- as.numeric(NR.chem.prior$AC50)
# Get rid of the entries that are NA:
NR.chem.prior <- subset(NR.chem.prior,!is.na(AC50))
# The reference chemicals are the ones in this table
Ref.chems <- unique(NR.chem.prior$CAS)
# The NRs are also set by the chemical prior table:
NRs <- sort(unique(toupper(NR.chem.prior$NR)))
Num.NR <- length(NRs)
```

heatmap.3.r

```
heatmap.3 <- function(x,
                Rowv = TRUE, Colv = if (symm) "Rowv" else TRUE,
                distfun = dist,
                hclustfun = hclust,
                dendrogram = c("both","row", "column", "none"),
                symm = FALSE,
                scale = c("none","row", "column"),
                na.rm = TRUE,
                revC = identical(Colv,"Rowv"),
                add.expr,
                breaks,
                symbreaks = max(x < 0, na.rm = TRUE) || scale != "none",
                col = "heat.colors",
                colsep,
                rowsep,
                sepcolor = "white",
                sepwidth = c(0.05, 0.05),
                cellnote,
                notecex = 1,
                notecol = "cyan",
                na.color = par("bg"),
                trace = c("none", "column","row", "both"),
                tracecol = "cyan",
                hline = median(breaks),
                vline = median(breaks),
                linecol = tracecol,
                margins = c(5,5),
                ColSideColors,
                RowSideColors,
                side.height.fraction=0.3,
                cexRow = 0.2 + 1/log10(nr),
                cexCol = 0.2 + 1/log10(nc),
                labRow = NULL,
                labCol = NULL,
                key = TRUE,
                keysize = 1.5,
                density.info = c("none", "histogram", "density"),
                denscol = tracecol,
                symkey = max(x < 0, na.rm = TRUE) || symbreaks,
                densadj = 0.25,
                main = NULL,
                xlab = NULL,
                ylab = NULL,
                lmat = NULL,
                lhei = NULL,
                lwid = NULL,
                ColSideColorsSize = 1,
```

```
              RowSideColorsSize = 1,
              KeyValueName="Value",...){

  invalid <- function (x) {
   if (missing(x) || is.null(x) || length(x) == 0)
      return(TRUE)
   if (is.list(x))
      return(all(sapply(x, invalid)))
   else if (is.vector(x))
      return(all(is.na(x)))
   else return(FALSE)
  }

  x <- as.matrix(x)
  scale01 <- function(x, low = min(x), high = max(x)) {
    x <- (x - low)/(high - low)
    x
  }
  retval <- list()
  scale <- if (symm && missing(scale))
    "none"
  else match.arg(scale)
  dendrogram <- match.arg(dendrogram)
  trace <- match.arg(trace)
  density.info <- match.arg(density.info)
  if (length(col) == 1 && is.character(col))
    col <- get(col, mode = "function")
  if (!missing(breaks) && (scale != "none"))
    warning("Using scale=\"row\" or scale=\"column\" when breaks are",
      "specified can produce unpredictable results.", "Please consider using only one or the other.")
  if (is.null(Rowv) || is.na(Rowv))
    Rowv <- FALSE
  if (is.null(Colv) || is.na(Colv))
    Colv <- FALSE
  else if (Colv == "Rowv" && !isTRUE(Rowv))
    Colv <- FALSE
  if (length(di <- dim(x)) != 2 || !is.numeric(x))
    stop("`x' must be a numeric matrix")
  nr <- di[1]
  nc <- di[2]
  if (nr <= 1 || nc <= 1)
    stop("`x' must have at least 2 rows and 2 columns")
  if (!is.numeric(margins) || length(margins) != 2)
    stop("`margins' must be a numeric vector of length 2")
  if (missing(cellnote))
    cellnote <- matrix("", ncol = ncol(x), nrow = nrow(x))
  if (!inherits(Rowv, "dendrogram")) {
    if (((!isTRUE(Rowv)) || (is.null(Rowv))) && (dendrogram %in%
```

```
            c("both", "row"))) {
            if (is.logical(Colv) && (Colv))
                dendrogram <- "column"
            else dedrogram <- "none"
            warning("Discrepancy: Rowv is FALSE, while dendrogram is `",
                dendrogram, "'. Omitting row dendogram.")
        }
    }
    if (!inherits(Colv, "dendrogram")) {
        if (((!isTRUE(Colv)) || (is.null(Colv))) && (dendrogram %in%
            c("both", "column"))) {
            if (is.logical(Rowv) && (Rowv))
                dendrogram <- "row"
            else dendrogram <- "none"
            warning("Discrepancy: Colv is FALSE, while dendrogram is `",
                dendrogram, "'. Omitting column dendogram.")
        }
    }
    if (inherits(Rowv, "dendrogram")) {
        ddr <- Rowv
        rowInd <- order.dendrogram(ddr)
    }
    else if (is.integer(Rowv)) {
        hcr <- hclustfun(distfun(x))
        ddr <- as.dendrogram(hcr)
        ddr <- reorder(ddr, Rowv)
        rowInd <- order.dendrogram(ddr)
        if (nr != length(rowInd))
            stop("row dendrogram ordering gave index of wrong length")
    }
    else if (isTRUE(Rowv)) {
        Rowv <- rowMeans(x, na.rm = na.rm)
        hcr <- hclustfun(distfun(x))
        ddr <- as.dendrogram(hcr)
        ddr <- reorder(ddr, Rowv)
        rowInd <- order.dendrogram(ddr)
        if (nr != length(rowInd))
            stop("row dendrogram ordering gave index of wrong length")
    }
    else {
        rowInd <- nr:1
    }
    if (inherits(Colv, "dendrogram")) {
        ddc <- Colv
        colInd <- order.dendrogram(ddc)
    }
    else if (identical(Colv, "Rowv")) {
        if (nr != nc)
```

```
            stop("Colv = \"Rowv\" but nrow(x) != ncol(x)")
        if (exists("ddr")) {
            ddc <- ddr
            colInd <- order.dendrogram(ddc)
        }
        else colInd <- rowInd
    }
    else if (is.integer(Colv)) {
        hcc <- hclustfun(distfun(if (symm)
            x
        else t(x)))
        ddc <- as.dendrogram(hcc)
        ddc <- reorder(ddc, Colv)
        colInd <- order.dendrogram(ddc)
        if (nc != length(colInd))
            stop("column dendrogram ordering gave index of wrong length")
    }
    else if (isTRUE(Colv)) {
        Colv <- colMeans(x, na.rm = na.rm)
        hcc <- hclustfun(distfun(if (symm)
            x
        else t(x)))
        ddc <- as.dendrogram(hcc)
        ddc <- reorder(ddc, Colv)
        colInd <- order.dendrogram(ddc)
        if (nc != length(colInd))
            stop("column dendrogram ordering gave index of wrong length")
    }
    else {
        colInd <- 1:nc
    }
    retval$rowInd <- rowInd
    retval$colInd <- colInd
    retval$call <- match.call()
    x <- x[rowInd, colInd]
    x.unscaled <- x
    cellnote <- cellnote[rowInd, colInd]
    if (is.null(labRow))
        labRow <- if (is.null(rownames(x)))
            (1:nr)[rowInd]
        else rownames(x)
    else labRow <- labRow[rowInd]
    if (is.null(labCol))
        labCol <- if (is.null(colnames(x)))
            (1:nc)[colInd]
        else colnames(x)
    else labCol <- labCol[colInd]
    if (scale == "row") {
```

```
    retval$rowMeans <- rm <- rowMeans(x, na.rm = na.rm)
    x <- sweep(x, 1, rm)
    retval$rowSDs <- sx <- apply(x, 1, sd, na.rm = na.rm)
    x <- sweep(x, 1, sx, "/")
  }
  else if (scale == "column") {
    retval$colMeans <- rm <- colMeans(x, na.rm = na.rm)
    x <- sweep(x, 2, rm)
    retval$colSDs <- sx <- apply(x, 2, sd, na.rm = na.rm)
    x <- sweep(x, 2, sx, "/")
  }
  if (missing(breaks) || is.null(breaks) || length(breaks) < 1) {
    if (missing(col) || is.function(col))
      breaks <- 16
    else breaks <- length(col) + 1
  }
  if (length(breaks) == 1) {
    if (!symbreaks)
      breaks <- seq(min(x, na.rm = na.rm), max(x, na.rm = na.rm),
        length = breaks)
    else {
      extreme <- max(abs(x), na.rm = TRUE)
      breaks <- seq(-extreme, extreme, length = breaks)
    }
  }
  nbr <- length(breaks)
  ncol <- length(breaks) - 1
  if (class(col) == "function")
    col <- col(ncol)
  min.breaks <- min(breaks)
  max.breaks <- max(breaks)
  x[x < min.breaks] <- min.breaks
  x[x > max.breaks] <- max.breaks
  if (missing(lhei) || is.null(lhei))
    lhei <- c(keysize, 4)
  if (missing(lwid) || is.null(lwid))
    lwid <- c(keysize, 4)
  if (missing(lmat) || is.null(lmat)) {
    lmat <- rbind(4:3, 2:1)

    if (!missing(ColSideColors)) {
      #if (!is.matrix(ColSideColors))
      #stop("'ColSideColors' must be a matrix")
      if (!is.character(ColSideColors) || nrow(ColSideColors) != nc)
        stop("'ColSideColors' must be a matrix of nrow(x) rows")
      lmat <- rbind(lmat[1, ] + 1, c(NA, 1), lmat[2, ] + 1)
      #lhei <- c(lhei[1], 0.2, lhei[2])
       lhei=c(lhei[1], side.height.fraction*ColSideColorsSize/2, lhei[2])
```

```
    }

    if (!missing(RowSideColors)) {
      #if (!is.matrix(RowSideColors))
      #stop("'RowSideColors' must be a matrix")
      if (!is.character(RowSideColors) || ncol(RowSideColors) != nr)
        stop("'RowSideColors' must be a matrix of ncol(x) columns")
      lmat <- cbind(lmat[, 1] + 1, c(rep(NA, nrow(lmat) - 1), 1), lmat[,2] + 1)
      #lwid <- c(lwid[1], 0.2, lwid[2])
      lwid <- c(lwid[1], side.height.fraction*RowSideColorsSize/2, lwid[2])
    }
    lmat[is.na(lmat)] <- 0
  }

  if (length(lhei) != nrow(lmat))
    stop("lhei must have length = nrow(lmat) = ", nrow(lmat))
  if (length(lwid) != ncol(lmat))
    stop("lwid must have length = ncol(lmat) =", ncol(lmat))
  op <- par(no.readonly = TRUE)
  on.exit(par(op))

  layout(lmat, widths = lwid, heights = lhei, respect = FALSE)

  if (!missing(RowSideColors)) {
    if (!is.matrix(RowSideColors)){
        par(mar = c(margins[1], 0, 0, 0.5))
        image(rbind(1:nr), col = RowSideColors[rowInd], axes = FALSE)
    } else {
      par(mar = c(margins[1], 0, 0, 0.5))
      rsc = t(RowSideColors[,rowInd, drop=F])
      rsc.colors = matrix()
      rsc.names = names(table(rsc))
      rsc.i = 1
      for (rsc.name in rsc.names) {
        rsc.colors[rsc.i] = rsc.name
        rsc[rsc == rsc.name] = rsc.i
        rsc.i = rsc.i + 1
      }
      rsc = matrix(as.numeric(rsc), nrow = dim(rsc)[1])
      image(t(rsc), col = as.vector(rsc.colors), axes = FALSE)
      if (length(rownames(RowSideColors)) > 0) {
        axis(1, 0:(dim(rsc)[2] - 1)/max(1,(dim(rsc)[2] - 1)), rownames(RowSideColors), las = 2, tick =
FALSE)
      }
    }
  }

  if (!missing(ColSideColors)) {
```

```
    if (!is.matrix(ColSideColors)){
      par(mar = c(0.5, 0, 0, margins[2]))
      image(cbind(1:nc), col = ColSideColors[colInd], axes = FALSE)
    } else {
      par(mar = c(0.5, 0, 0, margins[2]))
      csc = ColSideColors[colInd, , drop=F]
      csc.colors = matrix()
      csc.names = names(table(csc))
      csc.i = 1
      for (csc.name in csc.names) {
        csc.colors[csc.i] = csc.name
        csc[csc == csc.name] = csc.i
        csc.i = csc.i + 1
      }
      csc = matrix(as.numeric(csc), nrow = dim(csc)[1])
      image(csc, col = as.vector(csc.colors), axes = FALSE)
      if (length(colnames(ColSideColors)) > 0) {
        axis(2, 0:(dim(csc)[2] - 1)/max(1,(dim(csc)[2] - 1)), colnames(ColSideColors), las = 2, tick = FALSE)
      }
    }
  }

  par(mar = c(margins[1], 0, 0, margins[2]))
  x <- t(x)
  cellnote <- t(cellnote)
  if (revC) {
    iy <- nr:1
    if (exists("ddr"))
      ddr <- rev(ddr)
    x <- x[, iy]
    cellnote <- cellnote[, iy]
  }
  else iy <- 1:nr
  image(1:nc, 1:nr, x, xlim = 0.5 + c(0, nc), ylim = 0.5 + c(0, nr), axes = FALSE, xlab = "", ylab = "", col = col,
breaks = breaks, ...)
  retval$carpet <- x
  if (exists("ddr"))
    retval$rowDendrogram <- ddr
  if (exists("ddc"))
    retval$colDendrogram <- ddc
  retval$breaks <- breaks
  retval$col <- col
  if (!invalid(na.color) & any(is.na(x))) { # load library(gplots)
    mmat <- ifelse(is.na(x), 1, NA)
    image(1:nc, 1:nr, mmat, axes = FALSE, xlab = "", ylab = "",
      col = na.color, add = TRUE)
  }
```

```
  axis(1, 1:nc, labels = labCol, las = 2, line = -0.5, tick = 0,
    cex.axis = cexCol)
  if (!is.null(xlab))
    mtext(xlab, side = 1, line = margins[1] - 1.25)
  axis(4, iy, labels = labRow, las = 2, line = -0.5, tick = 0,
    cex.axis = cexRow)
  if (!is.null(ylab))
    mtext(ylab, side = 4, line = margins[2] - 1.25)
  if (!missing(add.expr))
    eval(substitute(add.expr))
  if (!missing(colsep))
    for (csep in colsep) rect(xleft = csep + 0.5, ybottom = rep(0, length(csep)), xright = csep + 0.5 + sepwidth[1], ytop = rep(ncol(x) + 1, csep), lty = 1, lwd = 1, col = sepcolor, border = sepcolor)
  if (!missing(rowsep))
    for (rsep in rowsep) rect(xleft = 0, ybottom = (ncol(x) + 1 - rsep) - 0.5, xright = nrow(x) + 1, ytop = (ncol(x) + 1 - rsep) - 0.5 - sepwidth[2], lty = 1, lwd = 1, col = sepcolor, border = sepcolor)
  min.scale <- min(breaks)
  max.scale <- max(breaks)
  x.scaled <- scale01(t(x), min.scale, max.scale)
  if (trace %in% c("both", "column")) {
    retval$vline <- vline
    vline.vals <- scale01(vline, min.scale, max.scale)
    for (i in colInd) {
      if (!is.null(vline)) {
        abline(v = i - 0.5 + vline.vals, col = linecol,
          lty = 2)
      }
      xv <- rep(i, nrow(x.scaled)) + x.scaled[, i] - 0.5
      xv <- c(xv[1], xv)
      yv <- 1:length(xv) - 0.5
      lines(x = xv, y = yv, lwd = 1, col = tracecol, type = "s")
    }
  }
  if (trace %in% c("both", "row")) {
    retval$hline <- hline
    hline.vals <- scale01(hline, min.scale, max.scale)
    for (i in rowInd) {
      if (!is.null(hline)) {
        abline(h = i + hline, col = linecol, lty = 2)
      }
      yv <- rep(i, ncol(x.scaled)) + x.scaled[i, ] - 0.5
      yv <- rev(c(yv[1], yv))
      xv <- length(yv):1 - 0.5
      lines(x = xv, y = yv, lwd = 1, col = tracecol, type = "s")
    }
  }
  if (!missing(cellnote))
    text(x = c(row(cellnote)), y = c(col(cellnote)), labels = c(cellnote),
```

```
        col = notecol, cex = notecex)
  par(mar = c(margins[1], 0, 0, 0))
  if (dendrogram %in% c("both", "row")) {
    plot(ddr, horiz = TRUE, axes = FALSE, yaxs = "i", leaflab = "none")
  }
  else plot.new()
  par(mar = c(0, 0, if (!is.null(main)) 5 else 0, margins[2]))
  if (dendrogram %in% c("both", "column")) {
    plot(ddc, axes = FALSE, xaxs = "i", leaflab = "none")
  }
  else plot.new()
  if (!is.null(main))
    title(main, cex.main = 1.5 * op[["cex.main"]])
  if (key) {
    par(mar = c(5, 4, 2, 1), cex = 0.75)
    tmpbreaks <- breaks
    if (symkey) {
      max.raw <- max(abs(c(x, breaks)), na.rm = TRUE)
      min.raw <- -max.raw
      tmpbreaks[1] <- -max(abs(x), na.rm = TRUE)
      tmpbreaks[length(tmpbreaks)] <- max(abs(x), na.rm = TRUE)
    }
    else {
      min.raw <- min(x, na.rm = TRUE)
      max.raw <- max(x, na.rm = TRUE)
    }

    z <- seq(min.raw, max.raw, length = length(col))
    image(z = matrix(z, ncol = 1), col = col, breaks = tmpbreaks,
      xaxt = "n", yaxt = "n")
    par(usr = c(0, 1, 0, 1))
    lv <- pretty(breaks)
    xv <- scale01(as.numeric(lv), min.raw, max.raw)
    axis(1, at = xv, labels = lv)
    if (scale == "row")
      mtext(side = 1, "Row Z-Score", line = 2)
    else if (scale == "column")
      mtext(side = 1, "Column Z-Score", line = 2)
    else mtext(side = 1, KeyValueName, line = 2)
    if (density.info == "density") {
      dens <- density(x, adjust = densadj, na.rm = TRUE)
      omit <- dens$x < min(breaks) | dens$x > max(breaks)
      dens$x <- dens$x[-omit]
      dens$y <- dens$y[-omit]
      dens$x <- scale01(dens$x, min.raw, max.raw)
      lines(dens$x, dens$y/max(dens$y) * 0.95, col = denscol,
        lwd = 1)
      axis(2, at = pretty(dens$y)/max(dens$y) * 0.95, pretty(dens$y))
```

```
        title("Color Key\nand Density Plot")
        par(cex = 0.5)
        mtext(side = 2, "Density", line = 2)
    }
    else if (density.info == "histogram") {
        h <- hist(x, plot = FALSE, breaks = breaks)
        hx <- scale01(breaks, min.raw, max.raw)
        hy <- c(h$counts, h$counts[length(h$counts)])
        lines(hx, hy/max(hy) * 0.95, lwd = 1, type = "s",
            col = denscol)
        axis(2, at = pretty(hy)/max(hy) * 0.95, pretty(hy))
        title("Color Key\nand Histogram")
        par(cex = 0.5)
        mtext(side = 2, "Count", line = 2)
    }
    else title("Color Key")
  }
  else plot.new()
  retval$colorTable <- data.frame(low = retval$breaks[-length(retval$breaks)],
    high = retval$breaks[-1], color = retval$col)
  invisible(retval)
}
```

myclust.R

```
#Define custom dist and hclust functions for use with heatmaps
myclust=function(c) {hclust(c,method="average")}
na.dist <- function(x,method="euclidian",...) {
 t.dist <- dist(x,...)
 t.dist <- as.matrix(t.dist)
 t.limit <- 1.1*max(t.dist,na.rm=T)
 t.dist[is.na(t.dist)] <- t.limit
 t.dist <- as.dist(t.dist)
 return(t.dist)
}
```

plot.data-062915.R

```
#Load latest version of heatmap.3 function:
source("heatmap3.R")

#Clustering function that handles NA's:
source("myclust.R")

plot.data  <-  function(hepdata,NR.genes,Chem.Names,Chem.Full.Names,file.stub="Simplified-LTEA-Ref-
Data-")
{
 Num.Genes <- length(NR.genes)
 Num.Chems <- length(Chem.Names)

 max.conc <- max(hepdata$conc)

 hep.heatmap <- matrix(NA,nrow=Num.Genes,ncol=Num.Chems)
 rownames(hep.heatmap) <- NR.genes
 colnames(hep.heatmap) <- Chem.Full.Names
 for (this.gene in NR.genes)
  for (this.chem in Chem.Full.Names)
  {
   this.data <- subset(hepdata,gene==this.gene&casn==Chem.Names[Chem.Full.Names==this.chem])
   if (any(this.data$state==2))
   {
    ac50 <- subset(this.data,state==2)
    ac50 <- min(ac50$conc)
    hep.heatmap[this.gene,this.chem] <- max.conc-ac50
   } else if (any(this.data$state==3)) {
    ac50 <- subset(this.data,state==3)
    ac50 <- min(ac50$conc)
    hep.heatmap[this.gene,this.chem] <- -(max.conc-ac50)
   }
  }

 pdf(file=paste(file.stub,Sys.Date(),".pdf",sep=""))
 main_title="LTEA Gene Expr. Changes"
 heatmap.3(hep.heatmap,
      hclustfun=myclust,
      distfun=na.dist,
      na.rm = TRUE,
      scale="none",
      dendrogram="both",
      margins=c(10,10),
      Rowv=TRUE,
      Colv=TRUE,
      cexCol=0.8,
      cexRow=0.5,
      symbreaks=FALSE,
```

```
        key=TRUE,
        symkey=FALSE,
        density.info="none",
        trace="none",
        main=main_title,
 #        labRow=F,
        col=colorRampPalette(c("red","white", "green"))(n = 10),
        KeyValueName="Activity")
 dev.off()
}
```

plot_NR_graph_posterior-070615.R

```
plot_NR_graph_posterior <- function(Network.Up,Network.Down,threshold=0.0,filename="NR-Initial-Full-Ref-Post-",layout.style=1,drop.vertices=F)
{
 edge.weights <-NULL
 NR.Gene.Post.graph <- graph.empty(,directed=T)
 NR.Gene.Post.graph <- NR.Gene.Post.graph + vertices(rownames(Network.Up),color="Blue",size=5)
 NR.Gene.Post.graph <- NR.Gene.Post.graph + vertices(colnames(Network.Up),color="Red",size=8)
 for (this.gene in rownames(Network.Up))
  for (this.NR in colnames(Network.Up))
  {
   if (Network.Up[this.gene,this.NR]>threshold)
   {
    NR.Gene.Post.graph <- NR.Gene.Post.graph + edges(c(this.NR,this.gene),color="Green")
    edge.weights[length(edge.weights)+1] <- round(Network.Up[this.gene,this.NR]*100)/10
   }
   else if (Network.Down[this.gene,this.NR]>threshold)
   {
    NR.Gene.Post.graph <- NR.Gene.Post.graph + edges(c(this.NR,this.gene),color="Red")
    edge.weights[length(edge.weights)+1] <- round(Network.Down[this.gene,this.NR]*100)/10
   }
  }

 edge.weights <- edge.weights/max(edge.weights)*4

 if (drop.vertices) NR.Gene.Post.graph <- delete.vertices(NR.Gene.Post.graph,names(degree(NR.Gene.Post.graph)[degree(NR.Gene.Post.graph)==0]))

 pdf(file=paste(filename,Sys.Date(),".pdf",sep=""))
 if (layout.style==1)
 {
plot(NR.Gene.Post.graph,vertex.label.dist=.5,vertex.label.color="Black",layout=layout.circle,edge.width=edge.weights)
 }
 else if (layout.style==2) {
  layout <- layout.reingold.tilford(NR.Gene.Post.graph, circular=T)
plot(NR.Gene.Post.graph,vertex.label.dist=.5,vertex.label.color="Black",layout=layout.fruchterman.reingold,edge.width=edge.weights)
 }
 dev.off()

 png(file=paste(filename,Sys.Date(),".png",sep=""),width = 960, height = 960)
 if (layout.style==1)
 {
```

```
plot(NR.Gene.Post.graph,vertex.label.dist=.5,vertex.label.color="Black",layout=layout.circle,edge.width=
edge.weights)
  }
  else if (layout.style==2) {
    layout <- layout.reingold.tilford(NR.Gene.Post.graph, circular=T)

plot(NR.Gene.Post.graph,vertex.label.dist=.5,vertex.label.color="Black",layout=layout.fruchterman.reing
old,edge.width=edge.weights)
  }
  dev.off()

  return(NR.Gene.Post.graph)
}
```

plot-NR-AC50-prior-112415.R

```
NR.AC50.prior <- matrix(NA,nrow=Num.Chems,ncol=Num.NR)
rownames(NR.AC50.prior) <- Chem.Full.Names
colnames(NR.AC50.prior) <- NRs
for (i in 1:Num.Chems)
  for (j in 1: Num.NR)
    if (!is.na(NR.Chem.Prior.Mean[i,j]))
      NR.AC50.prior[i,j] <- NR.Chem.Prior.Mean[i,j]


#Load latest version of heatmap.3 function:
source("heatmap3.R")

#Clustering function that handles NA's:
source("myclust.R")

#Create heatmap using custom heatmap.3 source code loaded above
pdf(file=paste(FILENAME,"-NR-AC50-prior-",Sys.Date(),".pdf",sep=""))
main_title="Ref. Chem. NR Activity Prior"
heatmap.3(-NR.AC50.prior,
        hclustfun=myclust,
        distfun=na.dist,
        na.rm = TRUE,
        scale="none",
        dendrogram="both",
        margins=c(8,10),
        cexRow=1,
        Rowv=TRUE,
        Colv=TRUE,
        symbreaks=FALSE,
        key=TRUE,
        symkey=FALSE,
        density.info="none",
        trace="none",
        main=main_title,
#       labRow=F,
        col=colorRampPalette(c("blue","red"))(n = 20),
        KeyValueName="Potency (-Log AC50)")
dev.off()
```

plot-NR-inferrence-043018.r

```
set.seed("12345678")

#Load latest version of heatmap.3 function
source("heatmap3.R")

Chem.Possible.AC50s <- jags.data$Chem.Possible.AC50s
Concs.Tested <- jags.data$Concs.Tested

#Define custom dist and hclust functions for use with heatmaps
myclust=function(c) {hclust(c,method="average")}
na.dist <- function(x,method="euclidian",...) {
 t.dist <- dist(x,...)
 t.dist <- as.matrix(t.dist)
 t.limit <- 1.1*max(t.dist,na.rm=T)
 t.dist[is.na(t.dist)] <- t.limit
 t.dist <- as.dist(t.dist)
 return(t.dist)
}


means <- apply(out.mcmc,2,mean)
medians <- apply(out.mcmc,2,median)



toxcast <- read.delim("TOXCAST.tsv",sep="\t",stringsAsFactors=F)
toxcast$CAS.RN <- sapply(toxcast$CAS.RN,function(x) strsplit(x,"=")[[1]][2])

rownames(toxcast) <- toxcast$CAS.RN
toxcast["1162-65-8","PREFERRED.NAME"] <- "Aflatoxin B1"
toxcast["NOCAS_47351","PREFERRED.NAME"] <- "SSR 240612"
toxcast["NOCAS_47364","PREFERRED.NAME"] <- "SSR 103800"
toxcast["NOCAS_47389","PREFERRED.NAME"] <- "SAR 150640"
toxcast <- toxcast[Chem.Names,]
Chem.Full.Names <- toxcast$PREFERRED.NAME



NR.response <- matrix(0,nrow=length(Chem.Full.Names),ncol=Num.NR)
rownames(NR.response) <- Chem.Full.Names
colnames(NR.response) <- NRs
for (i in 1:Num.Chems)
  for (j in 1: Num.NR)
  {
   if (medians[paste("NR.AC50.index[",i,",",j,"]",sep="")] > max(Concs.Tested[i,],na.rm=T))
NR.response[i,j] <- NA
```

```
    else NR.response[i,j] <- medians[paste("NR.AC50.index[",i,",",j,"]",sep="")]
  }

NR.probability <- matrix(0,nrow=length(Chem.Full.Names),ncol=Num.NR)
rownames(NR.probability) <- Chem.Full.Names
colnames(NR.probability) <- NRs
for (i in 1:Num.Chems)
  for (j in 1: Num.NR)
  {
    NR.probability[i,j]                                                          <-
sum(out.mcmc[,paste("NR.AC50.index[",i,",",j,"]",sep="")]<max(Concs.Tested[i,],na.rm=T))/dim(out.mc
mc)[1]
  }


write.csv(cbind(Chem.Names,NR.response),file=paste(FILENAME,"NR-Response-
",Sys.Date(),".txt",sep=""))
write.csv(cbind(Chem.Names,NR.probability),file=paste(FILENAME,"NR-Probs-",Sys.Date(),".txt",sep=""))



#Create heatmap using custom heatmap.3 source code loaded above
pdf(file=paste(FILENAME,"NR-AC50-Posterior-",Sys.Date(),".pdf",sep=""))
main_title="LTEA Inferred NR Activity"
heatmap.3(-NR.response,
          hclustfun=myclust,
          distfun=na.dist,
          na.rm = TRUE,
          scale="none",
          dendrogram="both",
          margins=c(8,10),
          cexRow=1,
          Rowv=TRUE,
          Colv=TRUE,
          symbreaks=FALSE,
          key=TRUE,
          symkey=FALSE,
          density.info="none",
          trace="none",
          main=main_title,
 #        labRow=F,
          col=colorRampPalette(c("grey","yellow","red"))(n = 20),
          KeyValueName="Potency (-Log AC50)")
dev.off()
```

posterior-analysis-043018.r

```
library(ComplexHeatmap)
library(circlize)


#setwd("~/Desktop/heparg")

load("HepaRG-ToxCast-Apr2018All-PREJAGS-1-2018-04-23.RData")

load("HepaRG-ToxCast-Apr2018All-2018-04-30-2.RData")

coda.out <- coda.out[[1]]
NUM.SAMPLES <- dim(coda.out$mcmc[[1]])[1]
NUM.SAMPLES.USED.PER.CHAIN <- 1000

Chem.Possible.AC50s <- jags.data$Chem.Possible.AC50s


out.mcmc <-
as.matrix(coda.out$mcmc[[1]][sample(1:NUM.SAMPLES,NUM.SAMPLES.USED.PER.CHAIN),])
for (i in 2:length(coda.out$mcmc)) out.mcmc <-
rbind(out.mcmc,as.matrix(coda.out$mcmc[[i]][sample(1:NUM.SAMPLES,NUM.SAMPLES.USED.PER.CHAI
N),]))

for (i in 1:Num.Chems)
for (j in 1:Num.NR)
{
  out.mcmc[,paste("NR.AC50.index[",i,",",j,"]",sep="")] <-
Chem.Possible.AC50s[out.mcmc[,paste("NR.AC50.index[",i,",",j,"]",sep="")]]
}



source("plot-NR-inferrence-043018.R")

source("build-posterior-network-043018.R")

source("plot_NR_graph_posterior-070615.R")
NR.post.full <- plot_NR_graph_posterior(Network.Up,Network.Down,filename=paste(FILENAME,"-
NRgraph-Raw-Posterior-",sep=""))
threshold <-0.5
NR.post.reduced <-
plot_NR_graph_posterior(Network.Up,Network.Down,threshold=threshold,layout.style=2,filename=pas
te(FILENAME,"-NRgraph-Threshold",threshold,"-Posterior-",sep=""))
NR.post.reduced <-
plot_NR_graph_posterior(Network.Up,Network.Down,threshold=threshold,layout.style=2,filename=pas
te(FILENAME,"-NRgraph-Threshold",threshold,"-Dropped-Posterior-",sep=""),drop.vertices=T)
```

```
save(NR.post.reduced,file=paste(FILENAME,"-NRgraph-Threshold",threshold,"-Dropped-Posterior-Graph-",Sys.Date(),".RData",sep=""))

load("HepaRG-ToxCast-Apr2018All-PREJAGS.RData")


hep.heatmap <- matrix(0,ncol=Num.Genes,nrow=length(Chem.Full.Names))
colnames(hep.heatmap) <- NR.genes
rownames(hep.heatmap) <- Chem.Full.Names
NR.heatmap <- matrix(0,nrow=length(Chem.Full.Names),ncol=Num.NR)
colnames(NR.heatmap) <- NRs
rownames(NR.heatmap) <- Chem.Full.Names

rownames(NR.response) <- Chem.Full.Names
for (this.chem in Chem.Full.Names)
{
print(this.chem)
  max.conc <- max(Concs.Tested[Chem.Full.Names==this.chem],na.rm=T)
  for (this.gene in NR.genes)
  {
    this.data <- subset(hepdata,gene==this.gene&casn==Chem.Names[Chem.Full.Names==this.chem])
    if (any(this.data$state==2))
    {
      ac50 <- subset(this.data,state==2)
      ac50 <- min(ac50$conc)
      hep.heatmap[this.chem,this.gene] <- -(ac50-max.conc)
    } else if (any(this.data$state==3)) {
      ac50 <- subset(this.data,state==3)
      ac50 <- min(ac50$conc)
      hep.heatmap[this.chem,this.gene] <- ac50-max.conc
    }
  }
  for (this.NR in 1:Num.NR)
  {
    if (!is.na(NR.response[this.chem,this.NR])) NR.heatmap[this.chem,this.NR] <-max.conc-NR.response[this.chem,this.NR]
  }

}



ht1 = Heatmap(hep.heatmap, name = "ht1",show_row_names=F,col = colorRamp2(c(-3.5, 0, 3.5), c("red", "white", "green")),row_title = "ToxCast Chemicals",column_title="Active Ref. Chem. Genes",heatmap_legend_param = list(title = "Potency\n(Up/Down)", at = c(-3,-1, 0,1,3),labels =c("0.1 uM (down)","10 uM (down)","> 100 uM","10 uM (up)","0.1 uM (up)"),color_bar = "discrete"))
```

```
ht2 = Heatmap(NR.heatmap, name = "ht2",show_row_names=F,col = colorRamp2(c(-3.5, 0, 3.5), c("red", "white", "green")),row_title = "ToxCast Chemicals",column_title="Inferred NR Activation",show_heatmap_legend =F)

ht2+ht1
```

prepare-JAGS-run-012916.R

```
library(runjags)
library(parallel)

load(paste(FILENAME,"-",Sys.Date(),"-preJAGS.RData",sep=""))

# We need a function to turn NR states into binary numbers:
to.binary <- function(x,bits=5)
{
  tmp <- paste(sapply(strsplit(paste(rev(intToBits(x))),""),`[[`,2),collapse="")
  return(substr(tmp,nchar(tmp)-bits+1,nchar(tmp)))
}

# And we use this to build a table of which NR's are off (0) or on (1) for each state:
NR.cat.boolean <- NULL
for(i in 0:(2^Num.NR-1)) NR.cat.boolean <-
cbind(NR.cat.boolean,as.numeric(strsplit(to.binary(i,Num.NR),"")[[1]]))

# Create a small cluster with a core for each chain in the analysis:
cl2 <- makeCluster(NUM.CHAINS)
```

read-literature-prior-072017.R

```
# Read table of literature NR-gene interactions with direction (Nancy Baker):
NR.prior.information <-
read.xls(LITERATURE.NR.GENE.INTERACTIONS,skip=1,stringsAsFactors=F,sheet=2)

# Store all the genes from the literature in a vector:
NR.prior.genes <- unique(NR.prior.information$genesymbol)

#source("plot-lit-NR-Network-Raw-070615.R")

# Annotate the NRs in the literature table so that we have consistent terms:
NR.prior.information[NR.prior.information$Nuclear.receptor=="constitutive androstane receptor","NR"]
<- "CAR"
NR.prior.information[NR.prior.information$Nuclear.receptor=="farnesoid X-activated receptor","NR"] <-
"FXR"
NR.prior.information[NR.prior.information$Nuclear.receptor=="pregnane X receptor","NR"] <- "PXR"
NR.prior.information[NR.prior.information$Nuclear.receptor=="Receptors, Aryl Hydrocarbon","NR"] <-
"AHR"
NR.prior.information[NR.prior.information$Nuclear.receptor=="Receptors, Androgen","NR"] <- "AR"
NR.prior.information[NR.prior.information$Nuclear.receptor=="PPAR alpha","NR"] <- "PPARA"

NR.gene.prior.final <- subset(NR.prior.information,genesymbol%in%NR.genes)
Num.Lit.Obs <- dim(NR.gene.prior.final)[1]
NR.gene.prior.final$gene.id <- sapply(NR.gene.prior.final$genesymbol,function(x) which(x==NR.genes))
NR.gene.prior.final$NR.id <- sapply(NR.gene.prior.final$NR,function(x) which(x==NRs))
# We only want the genes that remain in the trimmed list:
NR.prior.genes <- unique(NR.gene.prior.final$genesymbol)

#source("plot-lit-NR-Network-final-070615.R")
```

ToxCast-NRInferrence-figure-052218.R

```
library(ComplexHeatmap)
library(circlize)


#setwd("~/Desktop/heparg")
setwd("L:/Lab/NCCT_ExpoCast/ExpoCast2018/heparg")

load("HepaRG-ToxCast-Apr2018All-PREJAGS-1-2018-04-23.RData")

load("HepaRG-ToxCast-Apr2018All-2018-04-30-2.RData")

coda.out <- coda.out[[1]]
NUM.SAMPLES <- dim(coda.out$mcmc[[1]])[1]
NUM.SAMPLES.USED.PER.CHAIN <- 1000

Chem.Possible.AC50s <- jags.data$Chem.Possible.AC50s


out.mcmc <-
as.matrix(coda.out$mcmc[[1]][sample(1:NUM.SAMPLES,NUM.SAMPLES.USED.PER.CHAIN),])
for (i in 2:length(coda.out$mcmc)) out.mcmc <-
rbind(out.mcmc,as.matrix(coda.out$mcmc[[i]][sample(1:NUM.SAMPLES,NUM.SAMPLES.USED.PER.CHAI
N),]))

for (i in 1:Num.Chems)
for (j in 1:Num.NR)
{
  out.mcmc[,paste("NR.AC50.index[",i,",",j,"]",sep="")] <-
Chem.Possible.AC50s[out.mcmc[,paste("NR.AC50.index[",i,",",j,"]",sep="")]]
}



source("plot-NR-inferrence-043018.r")

source("build-posterior-network-043018.R")

source("plot_NR_graph_posterior-070615.R")
NR.post.full <- plot_NR_graph_posterior(Network.Up,Network.Down,filename=paste(FILENAME,"-
NRgraph-Raw-Posterior-",sep=""))
threshold <-0.5
NR.post.reduced <-
plot_NR_graph_posterior(Network.Up,Network.Down,threshold=threshold,layout.style=2,filename=pas
te(FILENAME,"-NRgraph-Threshold",threshold,"-Posterior-",sep=""))
NR.post.reduced <-
plot_NR_graph_posterior(Network.Up,Network.Down,threshold=threshold,layout.style=2,filename=pas
te(FILENAME,"-NRgraph-Threshold",threshold,"-Dropped-Posterior-",sep=""),drop.vertices=T)
```

```
save(NR.post.reduced,file=paste(FILENAME,"-NRgraph-Threshold",threshold,"-Dropped-Posterior-Graph-",Sys.Date(),".RData",sep=""))

load("HepaRG-ToxCast-Apr2018All-PREJAGS.RData")


hep.heatmap <- matrix(0,ncol=Num.Genes,nrow=length(Chem.Full.Names))
colnames(hep.heatmap) <- NR.genes
rownames(hep.heatmap) <- Chem.Full.Names
NR.heatmap <- matrix(0,nrow=length(Chem.Full.Names),ncol=Num.NR)
colnames(NR.heatmap) <- NRs
rownames(NR.heatmap) <- Chem.Full.Names

rownames(NR.response) <- Chem.Full.Names
for (this.chem in Chem.Full.Names)
{
print(this.chem)
  max.conc <- max(Concs.Tested[Chem.Full.Names==this.chem],na.rm=T)
  for (this.gene in NR.genes)
  {
    this.data <- subset(hepdata,gene==this.gene&casn==Chem.Names[Chem.Full.Names==this.chem])
    if (any(this.data$state==2))
    {
      ac50 <- subset(this.data,state==2)
      ac50 <- min(ac50$conc)
      hep.heatmap[this.chem,this.gene] <- max.conc-ac50
    } else if (any(this.data$state==3)) {
      ac50 <- subset(this.data,state==3)
      ac50 <- min(ac50$conc)
      hep.heatmap[this.chem,this.gene] <- ac50-max.conc
    }
  }
  for (this.NR in 1:Num.NR)
  {
    if (!is.na(NR.response[this.chem,this.NR])) NR.heatmap[this.chem,this.NR] <-max.conc-NR.response[this.chem,this.NR]
  }

}




ht1 = Heatmap(hep.heatmap, name = "ht1",show_row_names=F,col = colorRamp2(c(-3.5, 0, 3.5), c("red", "white", "green")),row_title = "ToxCast Chemicals",column_title="Active Ref. Chem. Genes",heatmap_legend_param = list(title = "Potency\n(Up/Down)", at = c(-3,-1, 0,1,3),labels =c("0.1 uM (down)","10 uM (down)","> 100 uM","10 uM (up)","0.1 uM (up)"),color_bar = "discrete"))
```

```
ht2 = Heatmap(NR.heatmap, name = "ht2",show_row_names=F,col = colorRamp2(c(-3.5, 0, 3.5), c("red", "white", "green")),row_title = "ToxCast Chemicals",column_title="Inferred NR Activation",show_heatmap_legend =F)

ht2+ht1

write.csv(hep.heatmap,file=paste("ToxCast-GeneActivity-",Sys.Date(),".txt",sep=""))
write.csv(NR.heatmap,file=paste("ToxCast-NRInferrence-",Sys.Date(),".txt",sep=""))
```

JAGS Code for Bayesian Analysis

HepaRG-Ref-Step1-Jul17-2017-07-20.jags

```
#------------------------------------------------------------------------------
# Name:       HepaRG-Ref-Step1-Jul17-2017-07-20.jags
# Description: A JAGS model for drawing inference of Nuclear Receptor (NR)
#             activation from gene expression data
#
# Author:     John Wambaugh, based on code by Robert Pearce
#
# Created:    Thu Nov  2 15:02:27 2017
#------------------------------------------------------------------------------

model {

  for (i in 1:Num.Chems) {
    for (j in 1:Num.NR) {
      NR.AC50.index[i,j] ~ dcat(NR.Chem.Prior[i,,j])
      NR.AC50[i,j] <- Chem.Possible.AC50s[i,NR.AC50.index[i,j]]
    }
  }

  for (i in 1:Num.Chems) {
    for (j in 1:Num.Concs[i]) {
    NR.state[i,j] <- 1 + 1*step(Concs.Tested[i,j]-NR.AC50[i,1])
    }
  }

  # From mclark:
  # Half-cauchy as in Gelman 2006
  # If scale parameter in cauchy is 5, precision of z = 1/5^2 = 0.04
  # sigma int
  #   sd_int <- zInt/sqrt(chSqInt) # prior for sigma; cauchy = normal/sqrt(chi^2)
  #   zInt ~ dnorm(0, .04)I(0,)
  #   chSqInt ~ dgamma(0.5, 0.5)   # chi^2 with 1 d.f.
  # Carvalho Horseshoe prior as coded by Allen Riddell:
  tau ~ dt(0, 1, 1)T(0,)

  for (i in 1:Num.Genes) {
    for (j in 1:Num.NR) {
      lambda[i,j] ~ dt(0, 1, 1)T(0,)
      NR.Gene.Network[i,j] ~ dnorm(0,lambda[i,j] * tau)T(-50,50)
    }
  }

  Lit.Threshold ~ dunif(0,50)
  log.Lit.Obs.Precision ~ dunif(-7,1)
  Lit.Obs.Precision <- 10^log.Lit.Obs.Precision
```

```
  for (i in 1:Num.Lit.Obs) {
    Lit.Obs[i] ~ dnorm(abs(NR.Gene.Network[Lit.Obs.Gene[i],Lit.Obs.NR[i]]),Lit.Obs.Precision)
    is.Censored.Lit.Obs[i] ~ dinterval(Lit.Obs[i],Lit.Threshold)
  }

  for (i in 1:Num.Genes)
  {
    P2min[i] ~ dunif(0.0001,0.5)
    P3min[i] ~ dunif(0.0001,0.5)
  }

  Network.Response[,2] <- NR.Gene.Network

  P2 <- (Network.Response+abs(Network.Response))/2
  P3 <- (abs(Network.Response)-Network.Response)/2


# We have three behaviors we want to ensure:
# 1) P2 and P3 are always non-zero (assay noise or stimulus by other receptors
#    not modeled)
# 2) P1 does not go to zero when many NR's are active (assay noise again)
# 3) The stiffness of P1 -- is it switch like (immediately drops for any NR
#    activation) or more gradual?

# We also want to weight the data for the replicate plates, without having to
# replicate the data. Need to do a weird fix to the dcat distribution to do this.
# Basically, the density d for n replicates is d^n. In order to achieve this with
# a categorical distibution, we need a fourth category that soaks up the remaining
# probability, otherwise our correctin will get messed up when dcat renormalizes
# everything.

  for (i in 1:Num.Obs)
  {
    PW[i,1] <- 1 - P2min[Obs.Gene[i]] - P3min[Obs.Gene[i]]
    PW[i,2] <- P2min[Obs.Gene[i]]+P2[Obs.Gene[i],NR.state[Obs.Chem[i],Obs.Conc[i]]]
    PW[i,3] <- P3min[Obs.Gene[i]]+P3[Obs.Gene[i],NR.state[Obs.Chem[i],Obs.Conc[i]]]
    Obs.State[i] ~ dcat(PW[i,])
  }
}
```

HepaRG-Ref-Step2-Nov2017-2017-11-30.jags

```
#------------------------------------------------------------------------------
# Name:       HepaRG-Ref-Step2-Nov2017-2017-11-30.jags
# Description: A JAGS model for drawing inference of Nuclear Receptor (NR)
#             activation from gene expression data
#
# Author:     John Wambaugh, based on code by Robert Pearce
#
# Created:    Mon Dec  4 09:38:22 2017
#------------------------------------------------------------------------------

model {

  for (i in 1:Num.Chems) {
    for (j in 1:Num.NR) {
      NR.AC50.index[i,j] ~ dcat(NR.Chem.Prior[i,,j])
      NR.AC50[i,j] <- Chem.Possible.AC50s[i,NR.AC50.index[i,j]]
    }
  }

  for (i in 1:Num.Chems) {
    for (j in 1:Num.Concs[i]) {
    NR.state[i,j] <- 1 + 32*step(Concs.Tested[i,j]-NR.AC50[i,1]) + 16*step(Concs.Tested[i,j]-NR.AC50[i,2]) +
8*step(Concs.Tested[i,j]-NR.AC50[i,3]) + 4*step(Concs.Tested[i,j]-NR.AC50[i,4]) +
2*step(Concs.Tested[i,j]-NR.AC50[i,5]) + 1*step(Concs.Tested[i,j]-NR.AC50[i,6])
    }
  }

  # From mclark:
  # Half-cauchy as in Gelman 2006
  # If scale parameter in cauchy is 5, precision of z = 1/5^2 = 0.04
  # sigma int
  #   sd_int <- zInt/sqrt(chSqInt) # prior for sigma; cauchy = normal/sqrt(chi^2)
  #   zInt ~ dnorm(0, .04)I(0,)
  #   chSqInt ~ dgamma(0.5, 0.5)   # chi^2 with 1 d.f.
  # Carvalho Horseshoe prior as coded by Allen Riddell:
  tau ~ dt(0, 1, 1)T(0,)

  for (i in 1:Num.Genes) {
    for (j in 1:Num.NR) {
      lambda[i,j] ~ dt(0, 1, 1)T(0,)
      NR.Gene.Network[i,j] ~ dnorm(0,lambda[i,j] * tau)T(-50,50)
    }
  }

  Lit.Threshold ~ dunif(0,50)
  log.Lit.Obs.Precision ~ dunif(-7,1)
  Lit.Obs.Precision <- 10^log.Lit.Obs.Precision
```

```
  for (i in 1:Num.Lit.Obs) {
    Lit.Obs[i] ~ dnorm(abs(NR.Gene.Network[Lit.Obs.Gene[i],Lit.Obs.NR[i]]),Lit.Obs.Precision)
    is.Signif.Lit.Obs[i] ~ dinterval(Lit.Obs[i],Lit.Threshold)
  }

  for (i in 1:Num.Genes)
  {
    P2min[i] ~ dunif(0.0001,0.5)
    P3min[i] ~ dunif(0.0001,0.5)
  }

  Network.Response <- NR.Gene.Network %*% NR.cat.boolean

  P2 <- (Network.Response+abs(Network.Response))/2
  P3 <- (abs(Network.Response)-Network.Response)/2


# We have three behaviors we want to ensure:
# 1) P2 and P3 are always non-zero (assay noise or stimulus by other receptors
#    not modeled)
# 2) P1 does not go to zero when many NR's are active (assay noise again)
# 3) The stiffness of P1 -- is it switch like (immediately drops for any NR
#    activation) or more gradual?

# We also want to weight the data for the replicate plates, without having to
# replicate the data. Need to do a weird fix to the dcat distribution to do this.
# Basically, the density d for n replicates is d^n. In order to achieve this with
# a categorical distibution, we need a fourth category that soaks up the remaining
# probability, otherwise our correctin will get messed up when dcat renormalizes
# everything.

  for (i in 1:Num.Obs)
  {
    PW[i,1] <- 1 - P2min[Obs.Gene[i]] - P3min[Obs.Gene[i]]
    PW[i,2] <- P2min[Obs.Gene[i]]+P2[Obs.Gene[i],NR.state[Obs.Chem[i],Obs.Conc[i]]]
    PW[i,3] <- P3min[Obs.Gene[i]]+P3[Obs.Gene[i],NR.state[Obs.Chem[i],Obs.Conc[i]]]
    Obs.State[i] ~ dcat(PW[i,])
  }
}
```

HepaRG-ToxCast-Apr2018All-2018-04-23.jags

```
#-----------------------------------------------------------------------------
# Name:       HepaRG-ToxCast-Apr2018All-2018-04-23.jags
# Description: A JAGS model for drawing inference of Nuclear Receptor (NR)
#             activation from gene expression data
#
# Author:     John Wambaugh, based on code by Robert Pearce
#
# Created:    Mon Apr 23 10:16:18 2018
#-----------------------------------------------------------------------------

model {
 for (i in 1:Num.Chems) {
  for (j in 1:Num.NR) {
   NR.AC50.index[i,j] ~ dcat(NR.Chem.Prior[i,])
  }
 }

 for (i in 1:Num.Chems) {
  for (j in 1:Num.Concs[i]) {
  NR.state[i,j] <- 1 + 32*step(Concs.Tested[i,j]-Chem.Possible.AC50s[NR.AC50.index[i,1]]) + 16*step(Concs.Tested[i,j]-Chem.Possible.AC50s[NR.AC50.index[i,2]]) + 8*step(Concs.Tested[i,j]-Chem.Possible.AC50s[NR.AC50.index[i,3]]) + 4*step(Concs.Tested[i,j]-Chem.Possible.AC50s[NR.AC50.index[i,4]]) + 2*step(Concs.Tested[i,j]-Chem.Possible.AC50s[NR.AC50.index[i,5]]) + 1*step(Concs.Tested[i,j]-Chem.Possible.AC50s[NR.AC50.index[i,6]])
  }
 }

 P.multi ~ dmnorm(P.multi.mu,P.multi.omega)

 # Read in the P2 and P3 min from the multivariate normal:
 for (i in 1:Num.Genes)
 {
 # Multivariate normal distribution doesn't allow truncation:
  P2min[i] <- max(0.0001,min(0.5,P.multi[i]))
  P3min[i] <- max(0.0001,min(0.5,P.multi[Num.Genes+i]))
 }

 NR.Network.0 ~ dmnorm(rep(0,length(NR.network.prior.mu)),NR.network.prior.omega)
 NR.Network <- NR.network.prior.mu+NR.Network.0%*%NR.network.prior.mu

 # Read in the non-zero weights from the multivariate normal:
 for (i in 1:Num.non.zero.weights)
 {
  NR.Gene.Network[non.zero.gene[i],non.zero.NR[i]] <- NR.Network[i]
 }
```

```
  Network.Response <- NR.Gene.Network %*% NR.cat.boolean
  P2 <- (Network.Response+abs(Network.Response))/2
  P3 <- (abs(Network.Response)-Network.Response)/2


# We have three behaviors we want to ensure:
# 1) P2 and P3 are always non-zero (assay noise or stimulus by other receptors
#    not modeled)
# 2) P1 does not go to zero when many NR's are active (assay noise again)
# 3) The stiffness of P1 -- is it switch like (immediately drops for any NR
#    activation) or more gradual?

# We also want to weight the data for the replicate plates, without having to
# replicate the data. Need to do a weird fix to the dcat distribution to do this.
# Basically, the density d for n replicates is d^n. In order to achieve this with
# a categorical distibution, we need a fourth category that soaks up the remaining
# probability, otherwise our correctin will get messed up when dcat renormalizes
# everything.

  for (i in 1:Num.Genes)
  {
    for (j in 1: 64 )
    {
      PW[i,j,1] <- 1 - P2min[i] - P3min[i]
      PW[i,j,2] <- P2min[i]+P2[i,j]
      PW[i,j,3] <- P3min[i]+P3[i,j]
    }
  }

  for (i in 1:Num.Obs)
  {
    Obs.State[i] ~ dcat(PW[Obs.Gene[i],NR.state[Obs.Chem[i],Obs.Conc[i]],])
  }

}
```
